# LncRNAs expression profiling in normal ovary, benign ovarian cyst and malignant epithelial ovarian cancer

**DOI:** 10.1038/srep38983

**Published:** 2016-12-12

**Authors:** Huan Wang, Ziyi Fu, Chencheng Dai, Jian Cao, Xiaoguang Liu, Juan Xu, Mingming Lv, Yun Gu, Jingmin Zhang, Xiangdong Hua, Genmei Jia, Sujuan Xu, Xuemei Jia, Pengfei Xu

**Affiliations:** 1 Nanjing Maternal and Child Health Institute, Nanjing Maternal and Child Health Care Hospital, Obstetrics and Gynecology Hospital Affiliated to Nanjing Medical University, Nanjing 210004, China; 2 Department of Gynecology, Nanjing Maternal and Child Health Care Hospital, Obstetrics and Gynecology Hospital Affiliated to Nanjing Medical University, Nanjing 210004, China; 3 The First Clinical Medical College of Nanjing Medical University, Nanjing 210029, China; 4 Department of Pathology, Nanjing Maternal and Child Health Hospital, Obstetrics and Gynecology Hospital Affiliated to Nanjing Medical University, Nanjing, 210004, China; 5 Department of Clinical Laboratory, Nanjing Maternal and Child Health Care Hospital, Obstetrics and Gynecology Hospital Affiliated to Nanjing Medical University, Nanjing, 210004, China

## Abstract

Long noncoding RNA (lncRNA) has been recognized as a regulator of gene expression, and the dysregulation of lncRNAs is involved in the progression of many types of cancer, including epithelial ovarian cancer (EOC). To explore the potential roles of lncRNAs in EOC, we performed lncRNA and mRNA microarray profiling in malignant EOC, benign ovarian cyst and healthy control tissues. In this study, 663 transcripts of lncRNAs were found to be differentially expressed in malignant EOC compared with benign and normal control tissues. We also selected 18 altered lncRNAs to confirm the validity of the microarray analysis using quantitative real-time PCR (qPCR). Pathway and Gene Ontology (GO) analyses demonstrated that these altered transcripts were involved in multiple biological processes, especially the cell cycle. Furthermore, Series Test of Cluster (STC) and lncRNA-mRNA co-expression network analyses were conducted to predict lncRNA expression trends and the potential target genes of lncRNAs. We also determined that two antisense lncRNAs (RP11-597D13.9 and ADAMTS9-AS1) were associated with their nearby coding genes (FAM198B, ADAMTS9), which participated in cancer progression. This study offers helpful information to understand the initiation and development mechanisms of EOC.

Ovarian cancer is the fourth most common gynecologic malignancy in women and is currently the most deadly tumor^1^. Every year, 22,500 women develop this disease, and an estimated 14,000 ovarian cancer-related deaths occur in the USA^2^. Epithelial ovarian cancer (EOC) accounts for ~90% of all ovarian cancer cases and is generally diagnosed at an advanced stage^3^. Despite the advances in surgical techniques and conventional chemotherapy, the prognosis of EOC has not improved significantly, and the long-term survival (5 years or more) for EOC patients does not exceed 30%^4^. The pathogenesis of EOC is a complicated biological process that involves genetic and epigenetic alterations^5^,^6^. Previous studies have shown that malignant EOCs have genome-wide analysis results and microRNA expression and methylation profiles that are distinct from those of benign ovarian cysts or normal ovaries^7^,^8^,^9^. Therefore, a better understanding of the molecular mechanisms underlying EOC progression will lead to the development of better diagnostic approaches and more effective treatments for EOC.

With the advent of the post-genome era, it has become increasingly clear that long non-coding RNAs (lncRNAs) are pervasively transcribed in the genome^10^, and lncRNA is regarded as a new regulator in numerous biological processes^11^,^12^. LncRNAs, for which the transcripts are longer than 200 nt, are a class of non-coding RNA molecules that do not encode proteins^13^. lncRNAs are typically divided into intergenic, bidirectional, antisense, overlapping and intronic lncRNAs according to their genomic localization in relation to nearby coding genes^14^. A large number of lncRNAs exhibit a tissue- or cell type-specific pattern^15^,^16^ and display weaker evolutionary constraints and lower expression levels than protein-coding genes^17^. A growing body of evidence suggests an important role of lncRNAs in cancer, including EOC. Notably, several lncRNAs, such as HOTAIR^18^, HOST2^19^, ANRIL^20^ and LSINCT5^21^, could serve as pivotal regulators in the biological processes of EOC. Therefore, lncRNA expression profiles in malignant EOC will help us to better understand EOC pathogenesis.

In this study, we compared malignant EOC with benign ovarian cyst and normal ovarian epithelial tissues via high-throughput microarray analyses to identify differentially expressed lncRNAs. Altogether, 182 and 481 lncRNAs were upregulated and downregulated in malignant EOC tissues compared with benign ovarian cysts and normal controls (absolute fold-change ≥5, false discovery rate (FDR) <0.05), respectively. Moreover, for candidate lncRNAs that may play potentially important roles in malignant EOC, we examined the Gene Ontology (GO) enrichment of their associated protein-coding genes and performed pathway analyses. Our study not only confirmed our hypothesis that lncRNAs serve as a new layer of gene regulation in EOC but also provided further insight into ovarian tumor therapy.

## Results

### Differentially expressed lncRNAs in malignant EOC tissues compared with benign cysts and normal ovarian tissues

**Figure 1:**
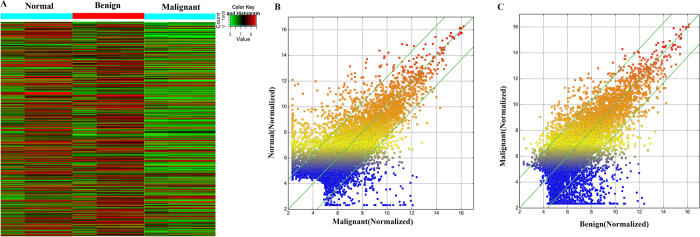
Differentially expressed lncRNAs in malignant EOC tissues compared with benign cysts and normal ovaries. (**A**) Differentially expressed lncRNAs among malignant EOC, benign ovarian cyst and normal control tissues were analyzed using hierarchical clustering; ‘red’ indicates high relative expression, and ‘green’ indicates low relative expression. (**B** and **C**) A scatter plot is used to assess lncRNA expression variations between malignant EOC and the normal ovary tissues (**B**) as well as between malignant EOC and benign ovarian cyst tissues (**C**). lncRNAs above the top green line and below the green line exhibited a greater than 5.0-fold change.

**Figure 2:**
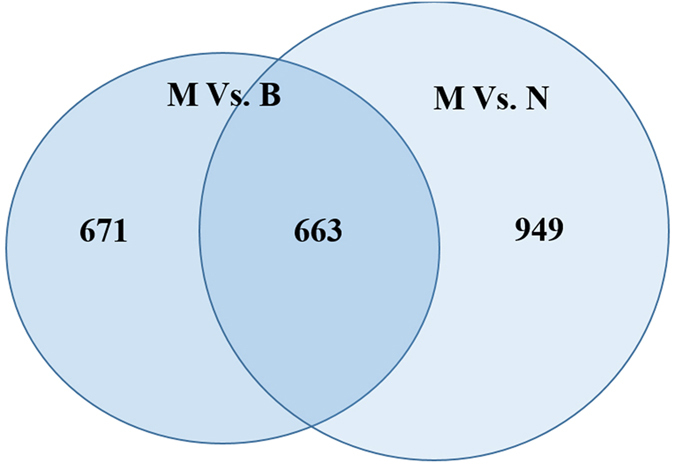
Flow diagram for the selection of lncRNAs for verification by qPCR. Number of differentially expressed lncRNAs (absolute fold change ≥5.0) in malignant EOC compared with benign and normal control samples identified by microarray profiling. Differentially expressed lncRNAs were defined using an FDR < 0.05.

Normal ovarian, benign ovarian cyst and malignant EOC tissues were obtained from Nanjing Maternal and Child Health Care Hospital (Supplementary Table S1) and identified by Hematoxylin and Eosin (HE) staining (Supplementary Figure S1). To explore the dysregulated lncRNAs in malignant EOC, we determined the lncRNA and mRNA expression profiles using microarray analyses of normal ovary, benign and malignant EOC tissues. Heatmaps and scatter-plots were used to assess the variation in lncRNA expression among normal ovarian, benign ovarian cyst and malignant EOC tissues (Fig. 1). We first selected those lncRNAs which were not only differentially expressed between malignant EOC and healthy ovarian but also altered between malignant EOC and benign ovarian cyst. Based on the microarray results, all lncRNAs and mRNAs with a signal altered by 5-fold and with FDR < 0.05 were identified as statistically altered. In total, 182 upregulated and 481 downreguated transcripts of lncRNAs were identified to be differently altered in malignant EOC compared with the benign cyst as well as the normal ovary groups (Fig. 2). The list of the all up- and down-regulated lncRNAs (absolute fold change ≥5) identified in the microarray analyses is presented in Supplementary Tables S2 and S3.

### Validation of the microarray data using Quantitative Real-time PCR (qPCR)

**Figure 3:**
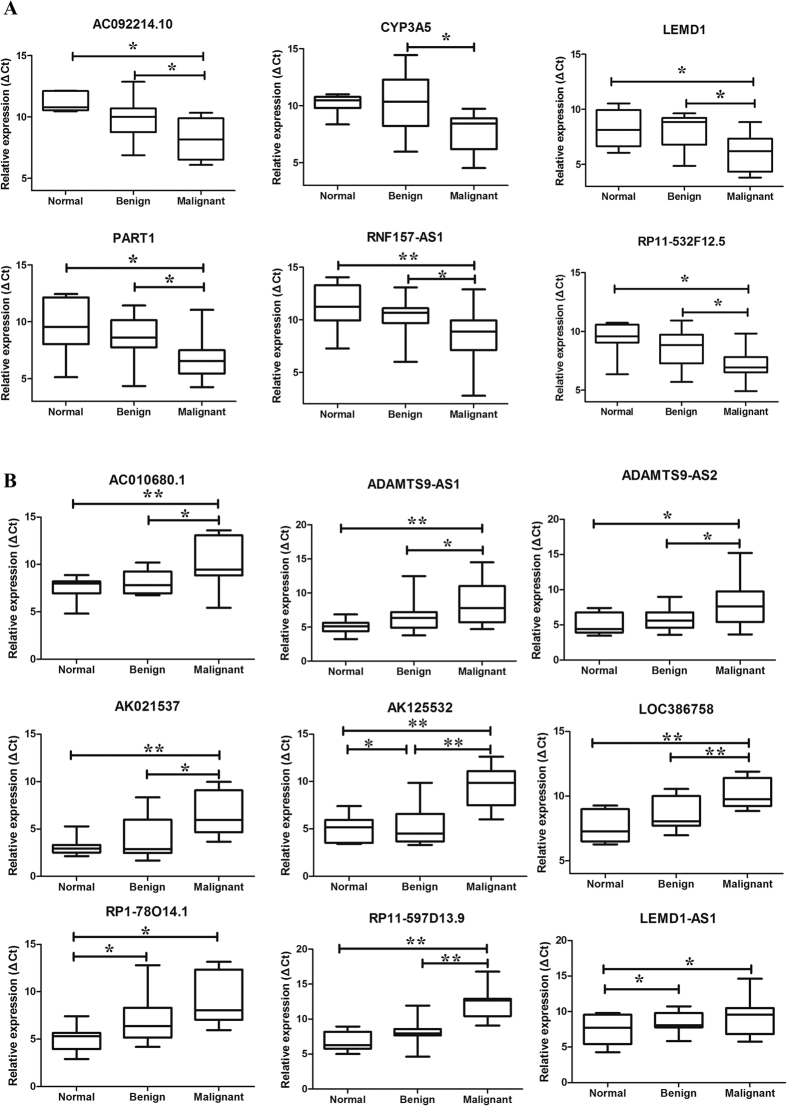
Validation by qPCR of candidate lncRNAs among normal control tissues, benign ovarian cyst and malignant EOC. The relative expression levels of candidate lncRNAs were detected by qPCR in 8 normal ovaries, 17 benign cysts and 15 malignant EOC samples. The ΔCt values of the lncRNAs were determined by subtracting the GAPDH ΔCt value. A smaller ΔCt value indicates higher expression levels. The data are presented as the relative expression level in tumor tissues (shown as ΔCt). (**A**) Unregulated lncRNAs in malignant EOC compared with benign and normal control tissues. (**B**) lncRNAs downregulated in malignant EOC compared with benign and normal control tissues. All *p* < 0.05.

To verify the reliability of the microarray results, a qPCR assay was used to detect the expression levels of randomly selected lncRNAs in 8 normal ovaries, 17 benign ovarian cysts and 15 malignant EOC samples. Finally, 6 upregulated lncRNAs and 12 downregulated lncRNAs were selected from 663 dysregulated lncRNAs transcripts. All of the primers are presented in Supplementary Table S4. The qPCR results indicated that AC092214.10, CYP3A5, LEMD1, PART1, RNF157-AS1 and RP11-532F12.5 levels increased, whereas AC010680.1, ADAMTS9-AS1, ADAMTS9-AS2, AK021537, AK125532, GRTP1-AS1, LEMD1-AS1, LOC386758, LOC729970, RP1-7814.1, RP11-597D13.9 and LEMD1-AS1 levels decreased (Fig. 3A,B). The qPCR results were consistent with the expression trends of the microarray data.

### Bioinformatic analysis of differentially expressed lncRNAs

**Figure 4:**
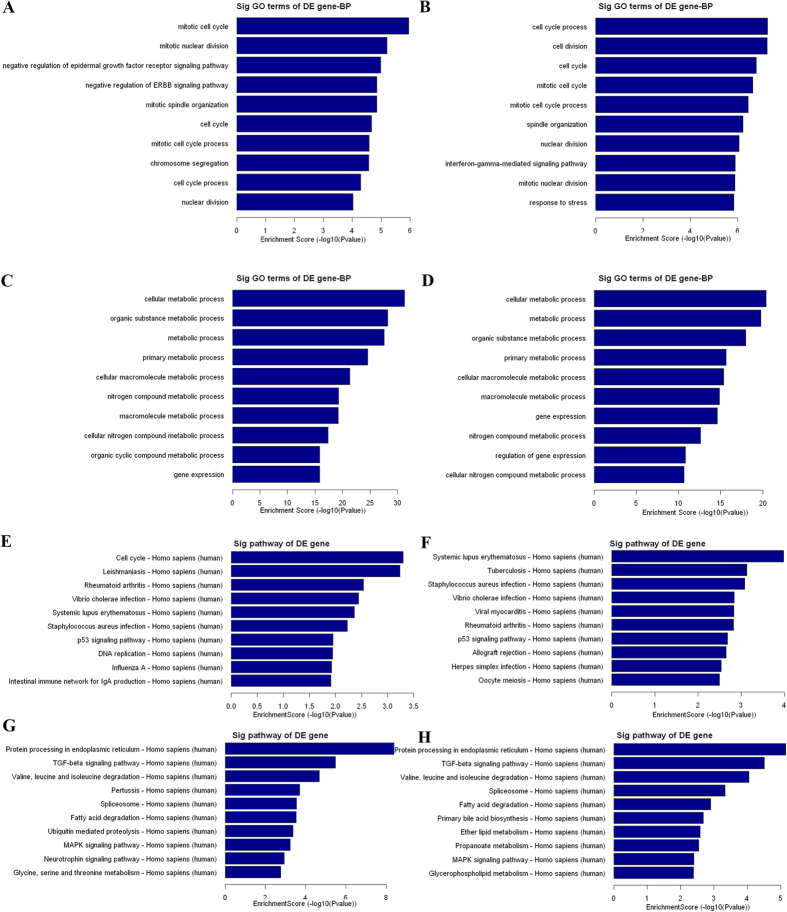
GO analysis and pathway analysis of the differentially expressed lncRNAs. (**A** and **B**) Top ten GO terms of biological processes for lncRNAs upregulated between malignant EOC and benign ovarian cyst and between malignant EOC and normal control tissues. (**C** and **D**) Top ten GO terms of biological processes for lncRNAs downregulated between malignant EOC and benign ovarian cyst and between malignant EOC and normal control samples. (**E** and **F**) Top ten pathways corresponding to the lncRNAs upregulated between malignant EOC and benign ovarian cyst and between malignant EOC and normal control tissues. (**G** and **H**) Top ten pathways corresponding to the lncRNAs downregulated between malignant EOC and benign ovarian cyst and between malignant EOC and normal control tissues.

To preliminarily explore the potential functions and mechanism of these dysregulated lncRNAs in malignant EOC, we performed GO and pathway analyses for the target genes of the differentially expressed lncRNAs. Previous studies have indicated that lncRNA is preferentially located near genes with developmental functions^14^. The GO project provides a controlled vocabulary to describe gene and gene product attributes in any organism and covers three domains: biological process (Fig. 4), cellular component (data not shown) and molecular function (data not shown). In our survey of biological processes, the neighboring coding gene functions of upregulated lncRNAs (M vs. N and M vs. B) were primarily involved in the mitotic cell cycle, mitotic nuclear division, and the cell cycle (Fig. 4A,B). In addition, the neighboring coding gene function of the downregulated lncRNAs primarily included cell metabolic process, organic substance metabolic process, and gene expression (Fig. 4C,D). Next, the pathway analysis revealed that these gene products participated in several signaling pathways. Regarding the upregulated transcripts (M vs. N and M vs. B), the main pathways involved systemic lupus erythematosus, *Staphylococcus aureus i*nfection and the p53 signaling pathway (Fig. 4E,F). We then observed that the most enriched network that corresponded to the downregulated transcripts in malignant EOC was “protein process in endoplasmic reticulum”. The other main pathways included the TGF-beta signaling pathway, valine, leucine and isoleucine degradation, the spliceosome, fatty acid degradation and the MAPK signaling pathway (Fig. 4G,H).

### LncRNA model profile analysis with Series Test of Cluster (STC)

**Figure 5:**
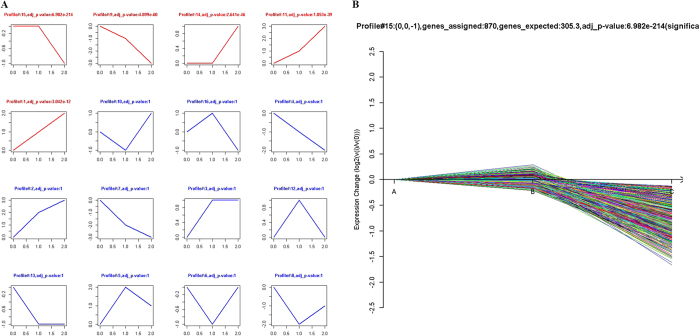
STC analysis of the differentially expressed lncRNAs related to malignant EOC. (**A**) The patterns of differentially expressed lncRNAs were analyzed, and sixteen model profiles were summarized. Each box represents a model expression profile. The upper number is the mode profile number which is used to summarize the different gene expression patterns. In total, 5 expression patterns of genes exhibited significant *p*-values (*p* < 0.05). Clusters are ordered based on the number of genes assigned. The number of transcripts assigned to each model profile was used as the estimate of the number of co-expressed lncRNAs, which was computed by the clustering method. (**B**) The most significant pattern of profile No. 15 is presented. A in the horizontal axis represents normal ovarian tissues, B represents benign ovarian cyst, and C denotes malignant EOC tissues. The vertical axis displays the time series of gene expression levels for the gene after Log2 normalized transformation.

To further assess the trend of differentially expressed lncRNAs among the normal ovary, benign ovarian cyst and malignant EOC samples, STC was used to reveal the corresponding changes in certain important lncRNA functional categories that were activated during each phase of EOC transformation. Each profile contains a cluster of multiple lncRNAs with similar expression patterns. As shown in Fig. 5A, sixteen model profiles were used to summarize the expression pattern of lncRNAs. Each box represents a model profile. Among the 16 patterns, five expression patterns, including profile Nos 15, 9, 14, 11 and 1, exhibited significant *p*-values (*p* < 0.05). Among the five significant profiles, profile No. 15 had the largest number of differentially expressed lncRNAs according to the *p*-values with 807 lncRNAs (Fig. 5B). Moreover, the lncRNA expression levels for these three clusters gradually increased (profile Nos 11 and 1) or gradually declined (profile No. 9) from normal ovary, benign cyst to malignant EOC (Supplementary Figure S2). The results of the STC analysis indicated that benign cyst may be the intermediate step from normal ovary to malignant EOC, and certain lncRNAs may play important roles during the development of EOC.

### Construction of the lncRNA-mRNA co-expression network

**Figure 6:**
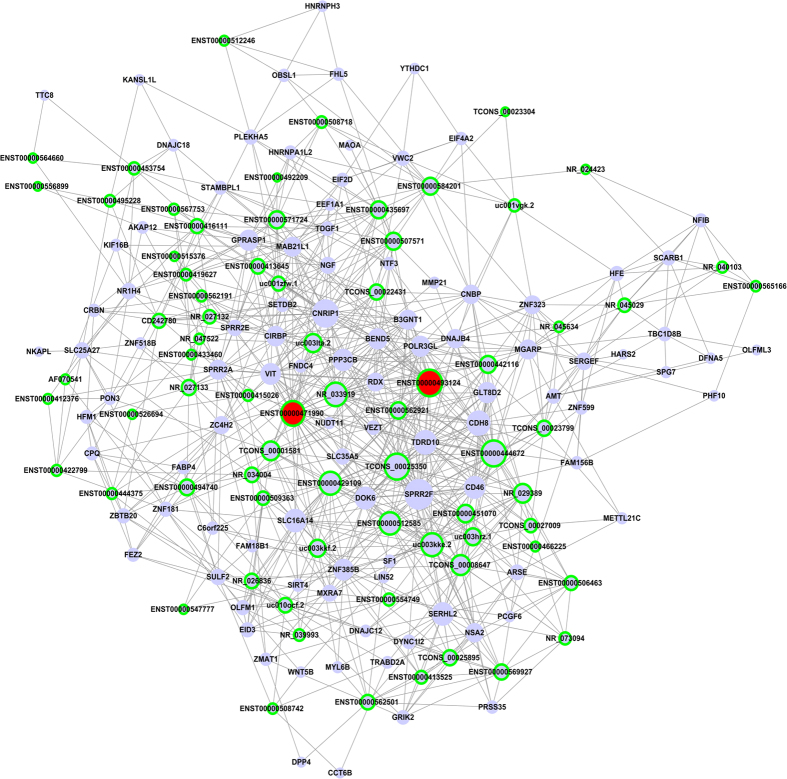
lncRNA-mRNA co-expression network in malignant EOC. The co-expression network of profile No. 15, which is the most significant pattern, was generated. In total, 72 lncRNAs and 100 protein-coding genes were identified in this pattern. In the network, blue ellipses represent mRNA, and green edges represent lncRNA. The size of nodes represents the power of the interrelation among the nodes, and edges between two nodes represent interactions between genes. The more edges on a gene, the more genes that connect to it and the more central a role the gene has within the network.

To further ascertain the lncRNAs that directly regulate the expression of target mRNAs and to identify the potential pivotal lncRNA, lncRNA-mRNA co-expression networks were generated. LncRNAs in profile 15 from the STC profiles were firstly analyzed and identified. Pearson correlation coefficients between all the aberrant lncRNAs and mRNAs were calculated. As shown in Fig. 6, the related gene co-expression networks extracted from profile No. 15 indicate that 72 lncRNAs and 100 protein-coding genes were identified as relevant (Supplementary Table S5A). In particular, the two transcripts of ADAMTS9-AS1 (ENST0000493124 and ENST00000471990) exhibited high scores (32 and 29, respectively) and high clustering coefficients (Supplementary Table S5B), suggesting that ADAMTS9-AS1 may play a pivotal role in malignant EOC. We also conducted the co-expression network of profile No. 9 (Supplementary Figure S3), and the relevant 19 lncRNAs and 48 mRNAs are presented in Supplementary Table S6.

### The correlation of lncRNAs and nearby coding genes

**Figure 7:**
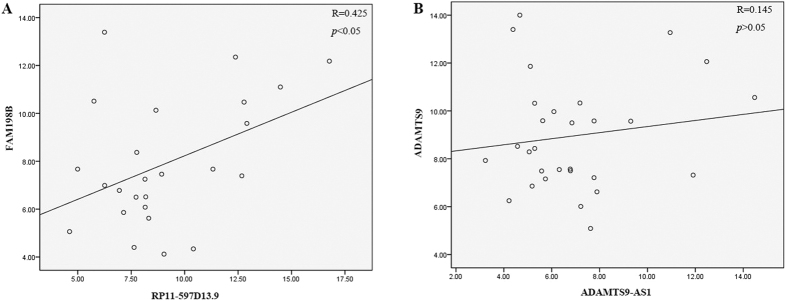
Candidate lncRNAs are specifically linked to neighboring protein-coding genes of malignant EOC transcriptional regulators. The co-expression of two lncRNAs with their neighboring protein-coding genes as analyzed by Spearman’s coefficients. The expression of RP11-597D13.9 was correlated with that of FAM198B (r = 0.425), but ADAMTS9-AS1 was not correlated with ADAMTS9 (r = 0.145).

Previous studies indicating that lncRNAs can affect the expression of coding gene in their proximity. We chose two lncRNAs (RP11-597D13.9 and ADAMTS9-AS1) to further explore the association between lncRNA expression and neighboring protein-coding genes. Based on UCSC database (http://genome.ucsc.edu/), the results of microarray have provided the nearby coding gene (Associated_gene in Supplementary Tables S2 and S3). RP11-597D13.9 is found to be located around FAM198B and ADAMTS9-AS1 is located near ADAMTS9 gene. To further confirm these co-expression relationships, we next detected the expression of FAM198B and ADAMTS9 with qPCR assays. Results revealed that the expression of FAM198B and RP11-597D13.9 were positively correlated (p < 0.05), and the correlation index was 0.425 (Fig. 7A). However, the expression of ADAMTS9 and ADAMTS9-AS1 had no correlation (*p* > 0.05, and the correlation index was equal to 0.125) (Fig. 7B).

## Discussion

Mammalian genomes encode thousands of lncRNAs. Accumulating evidence indicates that lncRNAs are emerging as important factors in gene regulatory networks and are involved in a wide range of biological processes^22^,^23^,^24^. To date, an increasing number of studies link dysregulation of lncRNAs to diverse human tumors, including EOC^25^. In our study, to select lncRNAs differentially expressed in malignant EOC using a more precise method, we detected the differentially expressed lncRNAs in patients with malignant EOC compared with those with benign ovarian cyst or healthy control subjects. To the best of our knowledge, this is the first report on lncRNA expression profiling in the normal ovary, benign cysts and malignant EOC.

Studies have revealed a critical role of lncRNA dysregulation in gene expression regulation and found that it contributed to oncogenesis and tumor progression^26^. Compared with protein-coding genes, lncRNAs exhibit greater tissue-specific, disease-specific and developmental stage-specific expression, and lncRNA expression is more closely associated with biological function and tumor status^27^,^28^, making lncRNAs attractive emerging molecular biomarkers and therapeutic targets for cancer diagnosis and therapeutics. Indeed, several dysregulated lncRNAs, such as HOTAIR^29^, FAL1^30^ and HOST2^19^, are associated with malignant EOC. In our study, 182 and 481 lncRNAs were upregulated and downregulated, respectively, in malignant EOC compared with benign and normal control tissues.

To select lncRNAs that play important roles in malignant EOC, we chose lncRNAs from those that were differentially expressed between malignant EOC tissues and healthy ovarian tissues and between malignant EOC tissues and benign ovarian cyst tissues to confirm the validity of the microarray results with qPCR assays. From the qPCR results, we found that most of the lncRNAs dysregulated in malignant EOC exhibited no significant difference from those in normal and benign ovarian tissues. This result was also supported by the STC results of the lncRNA expression patterns in profiles No. 15 and No. 14. These results of the STC analysis and qPCR assays consistently revealed that benign ovarian cyst may serve as controls for malignant EOC. Our results are also consistent with those of Patterson E *et al*.^31^ and Meyer-Rochow *et al*.^32^.

Notably, from the qPCR results, we also found that some lncRNAs, such as AK125532 and LEMD1-AS1, exhibit a substantial decreasing trend. To identify the differential expression patterns of lncRNAs among normal, benign and malignant tissues, an STC analysis was conducted. The lncRNAs in profile Nos 9, 11, and 1 exhibited a gradual increasing or decreasing trend from normal to benign to malignant EOC in the STC analysis. This finding may suggest that benign cyst transform to malignant EOC in a manner similar to that reported by Lim D *et al*., who observed that a cyst adenoma developed in a step-wise progression to serous carcinoma^33^. Moreover, studies by Powell D E *et al*. indicated that some genes, such as p53 and HER-2/neu, played a pivotal role in benign to malignant transformation^34^. Waldemarson S also reported that some proteins play important roles during the transition from benign to malignant ovarian cancer^35^. In our results, the lncRNAs in profile Nos 9, 11, and 1 may play important roles in the transformation of benign cyst to malignant EOC.

A large number of lncRNAs have been identified in tumors; however, the functions of lncRNAs remain poorly characterized. To infer the possible functional roles of the lncRNAs in malignant EOC, GO and pathway analyses were firstly used for the functional analysis of the lncRNA target gene pool. The GO analysis revealed that differentially expressed lncRNAs are most highly enriched in “cell cycle”, “cell division” and “cellular metabolic process”, which are closely associated with malignant EOC. Moreover, the pathway analysis indicated that “cell cycle”, the “p53 signaling pathway”, “protein processing in endoplasmic reticulum”, the “TGF-beta signaling pathway”, and the “MAPK signaling pathway” are involved in the pathogenic process of malignant EOC. Therefore, it is a reasonable inference that these dysregulated lncRNAs in malignant EOC act mainly by regulating the cell cycle and cell division, which then affect cancer cell proliferation. Qiu J *et al*. found that HOTAIR regulates the cell cycle and promotes ovarian cancer proliferation^18^, and Zhang E *et al*. reported that TUG1 affects lung cancer cell proliferation by altering cell cycle progression^36^. It has also been found that FAL1 regulates cell cycle progression by suppressing p21 expression in cancer^30^. Therefore, cell cycle regulation by lncRNAs may play a critical role in cancer, and our results indicated that differentially expressed lncRNAs perhaps primarily regulate the cell cycle and affect EOC progression.

To discern the key lncRNA associated with malignant EOC, we used a computational method to integrate lncRNA and mRNA co-expression networks. In total, 72 lncRNAs and 100 mRNAs were identified as having crucial regulatory roles in the STC profile No. 15. ADAMTS9-AS1, an antisense lncRNA, was identified as an important lncRNA with a higher score and higher clustering coefficient compared with other genes in the co-expression network. Accumulating studies have indicated that a number of antisense lncRNAs, such as PCNA-AS1^37^ and ZEB1-AS1^38^, play important roles in the complicated and accurate gene-networks of tumors. Therefore, we next chose two antisense lncRNAs, RP11-597D13.9 and ADAMTS9-AS1, to verify the function and mechanism of action of these lncRNAs. In contrast to miRNAs, there is currently no uniform mechanism to predict the function of lncRNAs. However, recent studies have found that many lncRNAs, especially antisense lncRNAs, can affect the expression of neighboring or overlapping coding genes. To further identify the potential mechanism of the nearby protein-coding gene, our qPCR assay results showed that RP11-597D13.9 has a positive correlation with its nearby gene, FAM198B. However, the expression levels of ADAMTS9 and ADAMTS9-AS1 were not correlated. It is possible that the lncRNA ADAMTS9-AS1 has other modes of function in malignant EOC.

In conclusion, our study used Affymetrix microarray analysis to show, for the first time, that lncRNAs were aberrantly expressed among normal ovary control, benign ovarian cyst and malignant EOC samples. In total, 663 transcripts of lncRNAs were found to be differentially expressed between malignant EOC and benign ovarian cyst tissues and between malignant EOC and the normal ovary control tissues (absolute fold changes ≥5, FDR < 0.05). We further selected six upregulated lncRNAs and twelve downregulated lncRNAs to verify consistency with the microarray result using a qPCR assay. We also applied an integrative approach to analyze the function of the lncRNAs. Thus, our data not only provide a comprehensive analysis of lncRNAs but also lay a good foundation for addressing the functions and mechanisms of lncRNAs.

## Materials and Methods

### Patient sample preparation

Tissue samples including malignant EOC, benign ovarian cyst and normal ovary tissues were obtained from surgical specimens at Nanjing Maternity and Child Health Care Hospital (Nanjing, China). Informed consent was obtained from all subjects. All of these specimens were snap-frozen in liquid nitrogen after excision. The histology of the tissue specimens was confirmed by a pathologist using hematoxylin and eosin staining. Of these samples, 9 samples were used for the lncRNA microarray analysis, and the remaining samples were assessed by qPCR for further validation. The experimental protocols were approved by the Ethics Committee of the Nanjing Maternity and Child Health Care Hospital. The methods were performed in accordance with the approved guidelines by the Ethics Committee of the Nanjing Maternity and Child Health Care Hospital. The clinical characteristics of the EOC patients are presented in Supplementary Table S1.

### RNA extraction

Total RNA was extracted from the frozen tissue block using Trizol reagent (Invitrogen, Carlsbad, CA, USA) according to the manufacturer’s protocol. The quantity and purity of the extracted RNA were detected using NanoDrop technology (Agilent, Santa Clara, CA, USA). The OD 260/280 absorbance ratios were between 1.8 and 2.0 for all the samples. RNA integrity was assessed using standard denaturing agarose gel electrophoresis. Final RNA preparations were resuspended in RNase-free water and stored at −80 °C.

### Microarray assay

Arraystar Human lncRNA Microarray V3.0 is designed for the global profiling of human lncRNAs and protein-coding transcripts, which is updated from the previous Microarray V2.0. Approximately 30,586 lncRNAs and 26,109 coding transcripts can be detected with this third-generation lncRNA microarray. The lncRNAs are carefully collected from the most authoritative databases, such as RefSeq, UCSC Knowngenes, and Ensembl, and many related studies.

Sample labeling and array hybridization were performed according to the Agilent One-Color Microarray-Based Gene Expression Analysis protocol (Agilent Technology) with minor modifications. Briefly, RNA was purified from total RNA after the removal of rRNA (mRNA-ONLY™ Eukaryotic mRNA Isolation Kit, Epicentre). Then, each sample was amplified and transcribed into fluorescent cRNA along the entire length of the transcripts without 3′ bias utilizing a random priming method (Arraystar Flash RNA Labeling Kit, Arraystar). The labeled cRNAs were purified using the RNeasy Mini Kit (Qiagen). The concentration and specific activity of the labeled cRNAs (pmol Cy3/μg cRNA) were measured by NanoDrop ND-1000. A total of 50 μl of hybridization solution was dispensed into the gasket slide and assembled with the lncRNA expression microarray slide. The slides were incubated for 17 hours at 65 °C in an Agilent Hybridization Oven. The hybridized arrays were washed, fixed and scanned using the Agilent DNA Microarray Scanner (part number G2505C). Agilent feature extraction software was used to analyze the acquired array images. Quantile normalization and subsequent data processing were performed using the GeneSpring GX v12.1 software package (Agilent Technologies). Fold-change (malignant vs. benign, malignant vs. normal) and *p*-values were calculated from the normalized expression levels.

### qPCR

Purified total RNA (1 μg) was reverse transcribed using the PrimeScript RT reagent kit (Takara, Japan), and qPCR was performed with an ABI VII7 PCR System (Applied Biosystems, USA) using Power SYBR Green PCR Master Mix (2X Applied Biosystems) following the manufacturer’s guidelines. Briefly, the mixture of samples was incubated at 95 °C for 10 min for an initial denaturation, followed by 40 PCR cycles of incubation at 95 °C for 15 s, 60 °C for 30 s, and then 72 °C for 30 s. The specific primer sequences for qPCR are listed in Supplementary Table 4. The expression levels of RNA were normalized to internal control GAPDH, and then calculated with the ΔCT method.

### GO and pathway analyses

Pathway analysis and GO analysis were applied to determine the potential roles of differentially expressed lncRNAs in biological pathways or GO terms. The predicted target genes of the differentially expressed lncRNAs were mapped to GO terms in the database for annotation, visualization, and integrated discovery (DAVID) (http://david.abcc.ncifcrf.gov/). The Gene Ontology is a controlled vocabulary composed of >38,000 precise defined phrases called GO terms that describe the molecular actions of gene products, the biological processes in which those actions occur and the cellular locations where they are present^39^. Fisher’s exact test is used to find if there have true difference between groups. In addition, we used the Kyoto Encyclopedia of Genes and Genomes (KEGG) (http://www.kegg.jp/) to confirm the pathway enrichment analysis. Pathway analysis usually was used to gain insight into the underlying biology of differentially expressed gene. The *p*-value denotes the significance of the pathway correlated to the conditions. Lower the *p*-value, more significant is the pathway^40^.

### STC analysis

This analysis were implemented according to previous research^41^. We first set normal, benign and malignant EOC as different point and then select a set of distinct and representative temporal expression profiles. These model profiles corresponded to possible profiles of lncRNA expression changes from normal, benign to malignant EOC samples. After the data logarithmic standardization, each lncRNA was assigned to the model profile that most closely matched the lncRNA’s expression profile as determined by the correlation coefficient. Since the model profiles were selected independent of the data, the algorithm could then determine which profiles have a statistically significant higher number of lncRNAs assigned using a permutation test. For example, we let the number of time points is n, every gene have *n*! permutation, for every permutation, we assigns each gene to the model profile that most closely matches the gene’s expression profile. 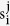 denote the number of gene that is assigned in *i* model profile in *j* permutation. We let 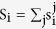. If the data is generated under the null hypothesis, 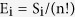 the predicted number of genes in the model profile. Note different model profiles have different number of genes, in general 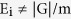. We assume the number of genes in the model profile obey the binomial distribution whose parameters are |G| and E_i_/|G|. We let t(m_i_) is the number of genes in the m_i_ th model profile. The *p-*value is p(X ≥ t(m_i_)), X~Bin(|G|, E_i_/|G|), so we can obtain the significant level of single model profile. Significant model profiles could either be analyzed independently or grouped together based on similarity to form clusters of significant profiles.

### LncRNA-mRNA co-expression network

The lncRNA-mRNA co-expression network was constructed based on the correlation between the differentially expressed lncRNAs and mRNAs. The algorithm utilized was from a previously described report^42^. We firstly selected lncRNAs from most significant profiles, named profile 15, to conducted lncRNA-mRNA co-expression network. In the network, blue ellipses represent mRNA, green edges represent lncRNA and the line between cycle nodes represent interactions between lncRNA and mRNA. Pearson correlation coefficients between all aberrant lncRNAs and mRNAs were calculated based on the *p*-value < 0.001 and absolute value of the correlation coefficient ≥0.90. The larger the value of the interaction, the stronger the likelihood that the two genes are co-expressed. The degree values represent the number of genes with which the gene can interact. The higher the degree, the more centrally the lncRNA or mRNA occurs within the network.

### Statistical analysis

All the statistical analyses were performed using the two-tailed Student’s *t*-test, ANOVA, and the Mann-Whitney test. Spearman correlation was used to examine the relationship between lncRNAs and their target coding genes. The data were presented as the means ± standard deviation. A value of FDR or *p*-value less than 0.05 was considered statistically significant. Computer-based calculations were conducted using SPSS version 20.0 (SPSS Inc., Chicago, IL, USA). The threshold value we used to screen differentially expressed lncRNA and mRNA was an absolute fold change ≥5.

## Additional Information

**How to cite this article**: Wang, H. *et al*. LncRNAs expression profiling in normal ovary, benign ovarian cyst and malignant epithelial ovarian cancer. *Sci. Rep.* **6**, 38983; doi: 10.1038/srep38983 (2016).

**Publisher's note:** Springer Nature remains neutral with regard to jurisdictional claims in published maps and institutional affiliations.

## Supplementary Material

Supplementary Dataset
