# Supplementary material for: LncRNAs expression profiling in normal ovary, benign ovarian cyst and malignant epithelial ovarian cancer
Source: Sci Rep. 2016 Dec 12;6:38983. doi: 10.1038/srep38983 (PMC5150236; doi:10.1038/srep38983)
Supplement: Supplementary Dataset [file srep38983-s1.pdf]

LncRNAs expression prolifing in normal ovary, benign ovarian cysts and malignant epithelial ovarian cancer

Huan Wang, Ziyi Fu, Chencheng Dai, Jian Cao, Xiaoguang Liu, Juan Xu, Mingming Lv, Yun Gu, Jingmin Zhang, Xiangdong Hua, Genmei Jia, Sujuan Xu, Xuemei Jia* and Pengfei Xu*

Supplementary Figures and Tables:

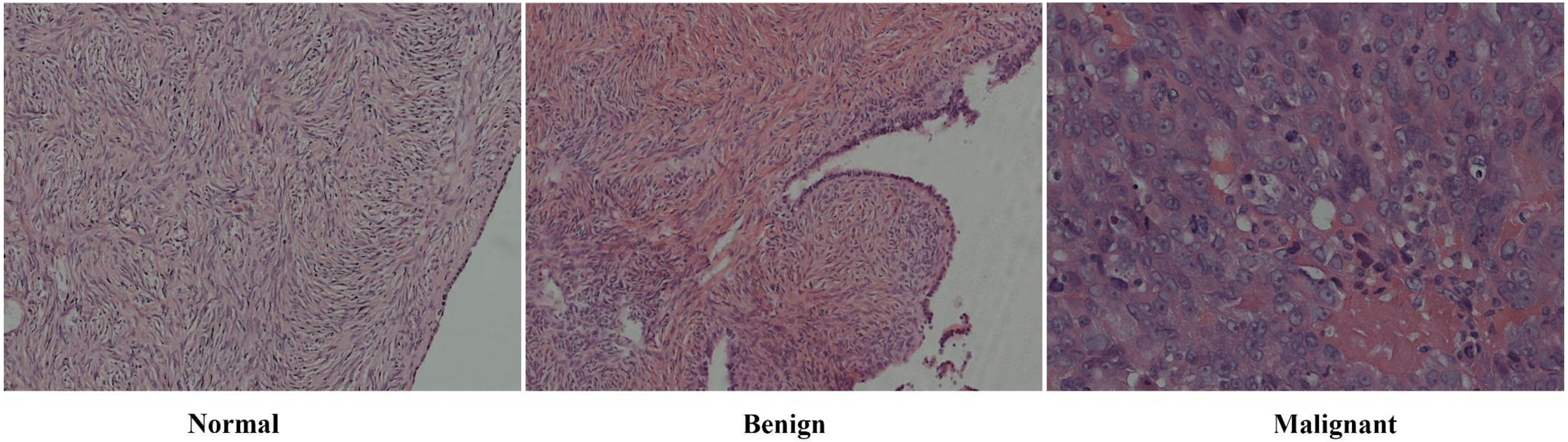

Figure S1. Normal, benign and malignant EOC tissues were identified by HE staining.

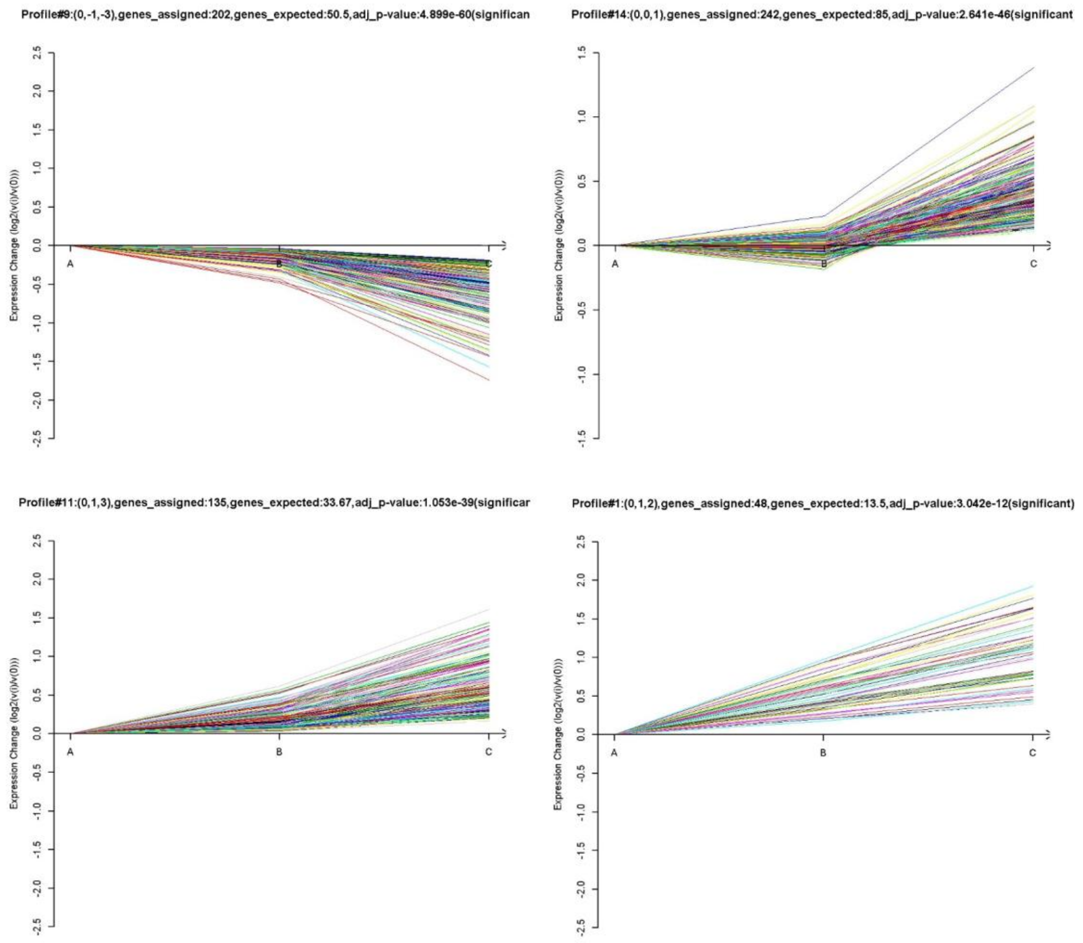

Figure S2. The significant pattern of profile NO.9, 14, 11 and 1. A in the horizontal axis means normal ovary tissues and B represent benign ovarian cyst and C denotes malignant EOC tissues. The vertical axis shows the time series of gene expression levels for the gene after Log2 normalized transformation.

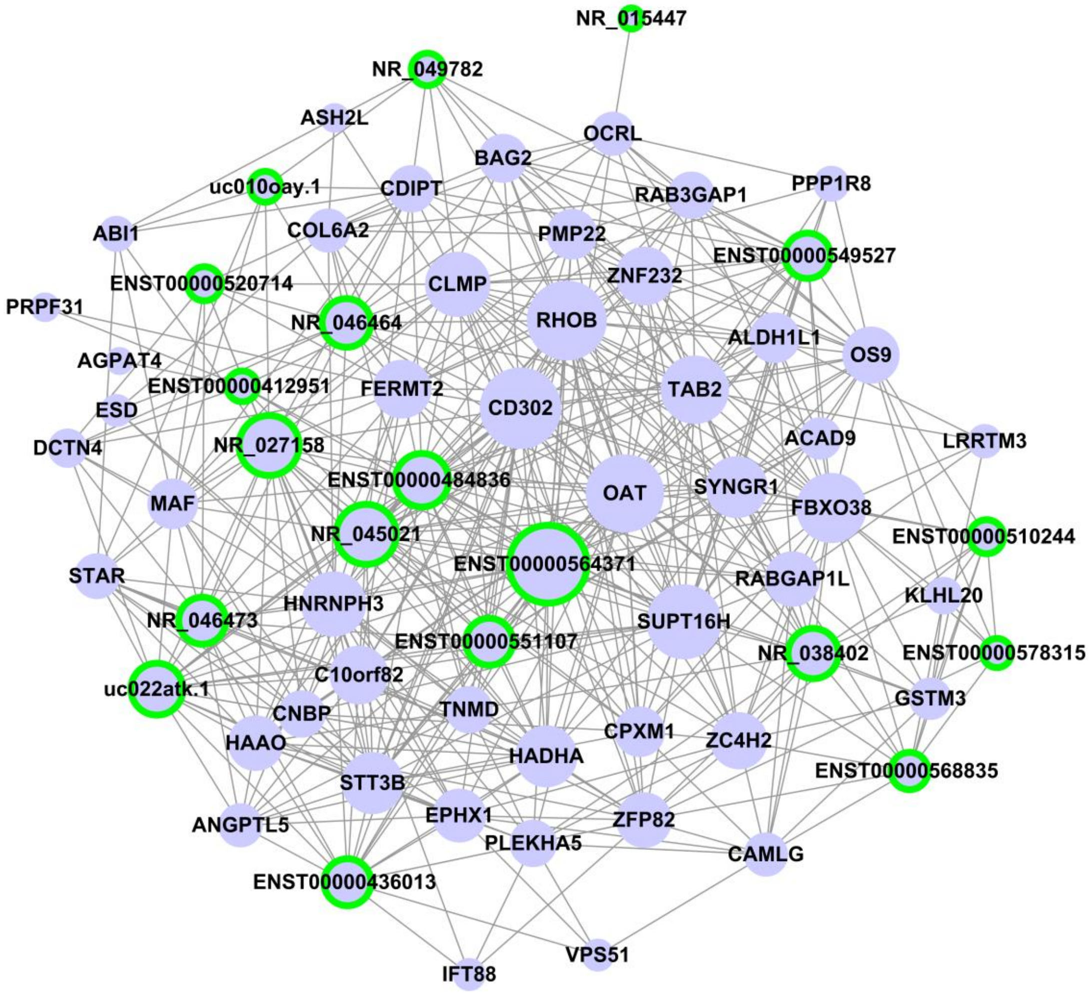

Figure S3. LncRNAs-mRNA co-expression network in profile NO.9. The co-expression network of profile NO.9. In the network, Blue ellipse represents as mRNA, green edge represents as lncRNA. The size of nodes represents the power of the interrelation among the nodes, and edges between two nodes represent interactions between genes. The more edges on a gene, the more genes connect to it, and the more central role it has within the network.

Table S1. Characteristics of patients

Patients	Ages （years）	Sample Type	Histologic Type	FIGO	Sample Use
1	43	normal			Array
2	35	normal			Array
3	48	normal			Array
4	35	benign	serous		Array
5	51	benign	serous		Array
6	40	benign	serous		Array
7	53	malignant	serous	IIIc	Array
8	45	malignant	serous	IIIc	Array
9	39	malignant	serous	IIc	Array
10	35	normal			qPCR
11	24	normal			qPCR
12	39	normal			qPCR
13	28	normal			qPCR
14	43	normal			qPCR
15	24	normal			qPCR
16	43	normal			qPCR
17	48	normal			qPCR
18	37	benign	serous		qPCR
19	39	benign	mucinous		qPCR
20	40	benign	serous		qPCR
21	25	benign	serous		qPCR
22	33	benign	mucinous		qPCR
23	25	benign	mucinous		qPCR
24	32	benign	mucinous		qPCR
25	51	benign	mucinous		qPCR
26	26	benign	serous		qPCR
27	43	benign	serous		qPCR
28	32	benign	mucinous		qPCR
29	25	benign	serous		qPCR
30	32	benign	serous		qPCR
31	27	benign	mucinous		qPCR
32	29	benign	serous		qPCR
33	39	benign	mucinous		qPCR

34	60	benign	serous		qPCR
35	49	malignant	endometrioid	IV	qPCR
36	49	malignant	serous	IIIc	qPCR
37	44	malignant	Germ Cell	IIIc	qPCR
38	65	malignant	mucinous	IV	qPCR
39	36	malignant	mucinous	IIc	qPCR
40	49	malignant	serous	IIIc	qPCR
41	47	malignant	serous	IIc	qPCR
42	41	malignant	serous	IIc	qPCR
43	30	malignant	mucinous	Ic	qPCR
44	46	malignant	serous	IIIc	qPCR
45	56	malignant	serous	IIc	qPCR
46	22	malignant	serous	IIIc	qPCR
47	25	malignant	serous	Ic	qPCR
48	73	malignant	serous	IIIc	qPCR
49	52	malignant	clear cell	IIIc	qPCR

Table S2. Up-regulated lncRNAs in malignant EOC compared with normal ovary and benign ovarian cyst.

GeneSymbol	Seqname	FDR M Vs. B	Fold Change M Vs. B	FDR M Vs. N	Fold Change M Vs. N	Source	Relationship	Associated_gene
SLC25A3P1	ENST00000563752	0.018	32.139	0.004	852.031	GENCODE	intergenic	
RNF157-AS1	NR_040017	0.013	28.366	0.004	611.092	RefSeq	natural antisense	FOXJ1
AC019117.1	ENST00000419463	0.018	16.686	0.004	557.098	GENCODE	intergenic	
XLOC_007235	TCONS_00015180	0.017	46.515	0.005	494.590	LincRNAs identified by Cabili et al	intergenic	
XLOC_008826	TCONS_00018520	0.016	29.093	0.003	452.068	LincRNAs identified by Cabili et al	intergenic	
RP4-797C5.2	ENST00000450480	0.014	25.395	0.003	451.846	GENCODE	intronic antisense	KCND2
BC034684	uc001gzl.3	0.006	23.851	0.002	427.464	UCSC_knowngene	exon sense-overlapping	CHI3L1
CP	NR_046371	0.026	30.047	0.006	341.051	RefSeq	exon sense-overlapping	CP
PSORS1C3	ENST00000412143	0.009	22.222	0.002	286.971	GENCODE	intron sense-overlapping	POU5F1
RP11-44F21.5	ENST00000567197	0.009	18.260	0.002	240.294	GENCODE	intergenic	
PRSS21	NR_073012	0.010	24.876	0.005	235.610	RefSeq	exon sense-overlapping	PRSS21
UCA1	uc002nbr.3	0.007	38.416	0.008	227.407	UCSC_knowngene	intergenic	
PART1	NR_028508	0.015	27.303	0.002	209.628	RefSeq	natural antisense	PDE4D
RMST	ENST00000547946	0.024	16.984	0.015	199.043	GENCODE	intergenic	
U47924.27	ENST00000537269	0.021	36.014	0.005	197.227	GENCODE	intergenic	
XLOC_011944	TCONS_00024657	0.016	31.147	0.003	186.702	LincRNAs identified by Cabili et al	intergenic	
RP11-7K24.3	ENST00000562471	0.012	20.382	0.008	173.191	GENCODE	intergenic	
LA16c-83F12.6	ENST00000453395	0.025	19.879	0.004	168.039	GENCODE	intergenic	
KIF25-AS1	ENST00000456585	0.014	26.622	0.004	163.354	GENCODE	intergenic	
LEMD1	NR_037583	0.014	18.914	0.008	157.942	RefSeq	exon sense-overlapping	LEMD1
RP11-483H20.4	ENST00000455074	0.006	16.060	0.006	129.131	GENCODE	natural antisense	ASB6
LOC100288181	NR_038925	0.042	8.032	0.007	104.910	RefSeq	natural antisense	LY6K
KIAA1324	NR_049773	0.016	30.266	0.009	100.702	RefSeq	exon sense-overlapping	KIAA1324
RP11-531A24.3	ENST00000564832	0.014	15.866	0.003	100.415	GENCODE	intergenic	
RP11-532F12.5	ENST00000568525	0.014	19.609	0.004	69.230	GENCODE	natural antisense	SPINT1
RP11-283G6.4	ENST00000540392	0.014	15.704	0.002	65.517	GENCODE	exon sense-overlapping	ITPR2
BC053669	uc003ttf.3	0.014	18.500	0.006	63.500	UCSC_knowngene	intron sense-overlapping	ZNF107
RP11-408H20.1	ENST00000568243	0.028	14.860	0.005	58.435	GENCODE	intergenic	

RP11-44K6.2	ENST00000520185	0.046	8.420	0.005	57.256	GENCODE	intron sense-overlapping	IDO1
RP11-369C8.1	ENST00000554810	0.019	14.048	0.006	55.750	GENCODE	intergenic	
LOC389641	NR_033928	0.018	20.003	0.009	53.383	RefSeq	bidirectional	TNFRSF10A
XLOC_012317	TCONS_00025507	0.015	18.990	0.004	48.291	LincRNAs identified by Cabili et al	intergenic	
XLOC_006742	TCONS_00014652	0.021	18.776	0.006	46.492	LincRNAs identified by Cabili et al	intergenic	
TUBA4B	ENST00000490341	0.018	12.057	0.004	45.652	GENCODE	intronic antisense	TUBA4A
AC073257.2	ENST00000413991	0.012	11.931	0.003	42.275	GENCODE	intergenic	
XLOC_012317	TCONS_00025506	0.018	16.973	0.002	41.317	LincRNAs identified by Cabili et al	intergenic	
RP11-95P2.3	ENST00000573220	0.024	10.964	0.006	39.838	GENCODE	intergenic	
BC043570	uc001zcp.1	0.015	5.850	0.004	39.739	UCSC_knowngene	intergenic	
RP11-283G6.5	ENST00000540625	0.033	7.326	0.005	37.188	GENCODE	natural antisense	SSPN
LOC285629	NR_027111	0.014	7.805	0.005	36.623	RefSeq	intergenic	
RP11-680F8.1	ENST00000560994	0.022	5.110	0.008	35.417	GENCODE	intergenic	
HOXD-AS1	ENST00000456876	0.016	25.153	0.009	34.816	GENCODE	intergenic	
ALOX12P2	ENST00000575787	0.037	11.477	0.006	34.552	GENCODE	intronic antisense	MIR497HG
RPS10P7	ENST00000441932	0.016	6.748	0.010	34.316	GENCODE	intergenic	
RP11-800A3.4	ENST00000565433	0.014	14.336	0.006	33.353	GENCODE	intergenic	
RP3-340N1.5	ENST00000442226	0.021	7.882	0.005	32.909	GENCODE	intronic antisense	UBXN10
RP11-532F12.5	ENST00000568419	0.014	16.286	0.004	32.196	GENCODE	natural antisense	SPINT1
3C12-49244600F4	ENST00000545775	0.024	7.977	0.004	31.461	GENCODE	intergenic	
RP11-283G6.5	ENST00000537525	0.006	8.475	0.004	31.329	GENCODE	natural antisense	SSPN
HOXD-AS1	NR_033979	0.010	15.623	0.007	31.274	RefSeq	natural antisense	HOXD1
RP11-532F12.5	ENST00000564302	0.015	11.036	0.003	30.756	GENCODE	natural antisense	SPINT1
RP11-319E16.2	ENST00000544842	0.021	12.949	0.009	30.108	GENCODE	intergenic	
RP11-1149O23.3	ENST00000522600	0.028	8.080	0.010	29.298	GENCODE	bidirectional	TNFRSF10A
RP11-445H22.4	ENST00000419931	0.028	6.341	0.004	27.316	GENCODE	intergenic	
CARM1P1	ENST00000497195	0.016	5.069	0.007	27.045	GENCODE	intergenic	
AK092541	uc003ncv.1	0.022	13.559	0.006	23.183	UCSC_knowngene	intergenic	
AL109706	uc021sxj.1	0.025	8.293	0.011	23.107	UCSC_knowngene	intergenic	
RP11-65J3.1	ENST00000444125	0.020	10.990	0.007	22.666	GENCODE	intergenic	
XLOC_002573	TCONS_00006781	0.036	5.388	0.003	21.885	LincRNAs identified by Cabili et al	intergenic	
LOC100652791	NR_045217	0.017	17.004	0.007	21.454	RefSeq	natural antisense	XKR5

RP1-223E5.4	ENST00000566170	0.017	9.605	0.005	21.444	GENCODE	natural antisense	SIRT5
HP08874	uc021onu.1	0.031	10.133	0.008	21.195	UCSC_knowngene	natural antisense	DHCR24
KRT18P55	ENST00000577198	0.014	13.162	0.005	20.983	GENCODE	intergenic	
TCAM1P	uc002jcf.3	0.021	17.255	0.011	20.936	UCSC_knowngene	intergenic	
RP11-307C12.11	ENST00000452962	0.026	16.876	0.005	20.634	GENCODE	natural antisense	DCST1
RP11-375B1.3	ENST00000512115	0.037	6.544	0.008	19.886	GENCODE	intergenic	
RP11-96D1.11	ENST00000571197	0.018	7.011	0.005	19.601	GENCODE	exon sense-overlapping	ESRP2
XLOC_014270	TCONS_00029615	0.033	6.575	0.028	19.520	LincRNAs identified by Cabili et al	intergenic	
OR7E14P	ENST00000526721	0.021	8.549	0.015	19.273	pseudogene		
AX746690	uc002xid.1	0.025	6.196	0.010	19.227	UCSC_knowngene	intergenic	
RP4-616B8.4	ENST00000570096	0.021	9.019	0.010	18.379	GENCODE	bidirectional	DHX35
BC005081	uc021pbg.1	0.013	18.485	0.009	18.331	UCSC_knowngene	natural antisense	BCAN
XLOC_004677	TCONS_00010209	0.015	11.534	0.002	18.285	LincRNAs identified by Cabili et al	intergenic	
OR7E91P	uc010fdz.3	0.018	7.507	0.004	18.211	UCSC_knowngene	intergenic	
HOXD-AS1	ENST00000546798	0.023	5.946	0.005	18.171	GENCODE	intergenic	
3C12-49244600F4	ENST00000535078	0.019	9.208	0.004	17.803	GENCODE	intergenic	
RP11-263K19.4	ENST00000453136	0.010	9.924	0.003	17.607	GENCODE	natural antisense	THBS3
DGUOK-AS1	ENST00000413452	0.036	10.854	0.011	17.384	GENCODE	natural antisense	DGUOK
RP11-10A14.5	ENST00000417333	0.034	12.535	0.009	16.929	GENCODE	intergenic	
RP11-473M20.11	ENST00000570700	0.024	6.528	0.005	15.097	GENCODE	intergenic	
AC004540.4	ENST00000451368	0.022	11.090	0.006	14.765	GENCODE	natural antisense	SNX10
BC069792	uc001jtm.3	0.022	6.347	0.008	14.671	UCSC_knowngene	natural antisense	NUDT13
RP11-629O1.2	ENST00000561761	0.021	7.310	0.009	14.599	GENCODE	bidirectional	ST3GAL1
LOC100289092	NR_046290	0.021	12.240	0.008	14.279	RefSeq	natural antisense	ATP2A1
RP11-513I15.6	ENST00000429998	0.038	7.413	0.016	14.172	GENCODE	intergenic	
RP11-363E7.4	ENST00000563205	0.027	7.605	0.005	13.957	GENCODE	intergenic	
RP11-387D10.2	ENST00000558105	0.020	7.837	0.011	13.721	GENCODE	bidirectional	BLM
RP11-10A14.5	ENST00000523747	0.018	15.720	0.009	13.692	GENCODE	intergenic	
CTD-3074O7.5	ENST00000527274	0.042	9.630	0.006	13.562	GENCODE	natural antisense	DPP3
CYP3A5	NR_033810	0.014	35.370	0.008	13.454	RefSeq	exon sense-overlapping	CYP3A5
AC004540.4	ENST00000451264	0.034	9.919	0.011	13.345	GENCODE	natural antisense	SNX10
RP11-423H2.1	ENST00000358442	0.018	5.357	0.004	13.307	GENCODE	intergenic	

CTD-2026G22.1	ENST00000529081	0.030	10.606	0.018	13.214	GENCODE	intergenic	
RP11-134G8.8	ENST00000430471	0.040	6.459	0.005	13.138	GENCODE	intergenic	
RP11-764K9.1	ENST00000417843	0.029	5.044	0.006	12.774	GENCODE	intergenic	
RP5-1125A11.1	ENST00000440595	0.029	6.996	0.005	12.645	GENCODE	intronic antisense	RALY
RP1-290I10.6	ENST00000420777	0.017	10.215	0.017	12.226	GENCODE	intronic antisense	TFAP2A
RP4-635E18.6	ENST00000435388	0.012	10.975	0.006	12.144	GENCODE	natural antisense	EXOSC10
RP11-44N21.1	ENST00000546968	0.022	6.169	0.003	11.793	GENCODE	intergenic	
CASC2	NR_026941	0.020	5.119	0.006	11.710	RefSeq	bidirectional	RAB11FIP2
RP3-380B8.4	ENST00000454882	0.021	8.101	0.014	11.240	GENCODE	intergenic	
AL833181	uc002sag.3	0.022	5.570	0.009	11.081	UCSC_knowngene	intron sense-overlapping	BCL11A
AK094859	uc001kyd.1	0.021	9.628	0.008	10.981	UCSC_knowngene	intergenic	
UBE2V1	NR_047553	0.019	6.120	0.004	10.635	RefSeq	exon sense-overlapping	UBE2V1
RP11-1L12.3	ENST00000531363	0.032	5.313	0.023	10.361	GENCODE	intergenic	
XLOC_008233	TCONS_00017369	0.018	7.182	0.007	10.296	LincRNAs identified by Cabili et al	intergenic	
RPL32P3	uc003emd.1	0.010	8.497	0.004	9.854	UCSC_knowngene	intergenic	
LINC00310	ENST00000428914	0.025	6.716	0.027	9.837	GENCODE	intergenic	
RP5-901A4.1	ENST00000532296	0.026	13.655	0.007	9.709	GENCODE	intronic antisense	ALDH3B1
RP11-383C5.5	ENST00000430970	0.033	5.998	0.016	9.516	GENCODE	bidirectional	C10orf137
DQ577420	uc002zdj.1	0.026	5.434	0.011	9.414	UCSC_knowngene	intronic antisense	HSF2BP
RP3-512B11.3	ENST00000561592	0.013	10.595	0.004	9.387	GENCODE	bidirectional	DSP
AP000344.4	ENST00000454268	0.005	8.026	0.006	9.214	GENCODE	intergenic	
RP11-314A20.2	ENST00000497885	0.022	5.060	0.007	9.100	GENCODE	natural antisense	PELP1
BC039551	uc003ine.3	0.024	14.621	0.009	9.046	UCSC_knowngene	intronic antisense	FHDC1
XLOC_011789	TCONS_00024467	0.044	5.348	0.017	9.026	LincRNAs identified by Cabili et al	intergenic	
RP11-314N13.3	ENST00000412497	0.037	6.049	0.005	8.922	GENCODE	intergenic	
RP11-147L13.2	ENST00000577698	0.017	5.972	0.009	8.886	GENCODE	natural antisense	AMZ2
AP000695.4	ENST00000454980	0.022	10.009	0.004	8.599	GENCODE	natural antisense	CLDN14
Z83851.4	ENST00000420096	0.011	5.596	0.006	8.376	GENCODE	intronic antisense	TCF20
RP11-322M19.1	ENST00000413961	0.021	9.201	0.011	8.294	GENCODE	intergenic	
RP11-334C17.5	ENST00000570309	0.027	5.059	0.013	8.290	GENCODE	natural antisense	CARD14
HCG4	NR_002139	0.038	6.582	0.004	8.286	RefSeq	natural antisense	LOC554223
RP11-277L2.3	ENST00000447883	0.021	7.863	0.007	8.171	GENCODE	intergenic	

EPR-1	uc021uec.1	0.036	39.328	0.006	8.037	UCSC_knowngene	natural antisense	BIRC5
RP11-53B5.1	ENST00000443771	0.010	10.055	0.004	7.995	GENCODE	intergenic	
AC007392.3	ENST00000433396	0.023	6.725	0.009	7.949	GENCODE	intergenic	
AK123617	uc002tji.1	0.020	5.808	0.009	7.784	UCSC_knowngene	intergenic	
XLOC_009185	TCONS_00019358	0.047	5.260	0.015	7.782	LincRNAs identified by Cabili et al	intergenic	
XLOC_004128	TCONS_00007884	0.014	5.347	0.004	7.770	LincRNAs identified by Cabili et al	intergenic	
HEXIM2	uc002iik.1	0.032	9.322	0.008	7.564	UCSC_knowngene	natural antisense	HEXIM2
XLOC_003422	TCONS_00008738	0.012	5.270	0.005	7.558	LincRNAs identified by Cabili et al	intergenic	
CTD-3074O7.5	ENST00000533502	0.012	9.824	0.005	7.505	GENCODE	intronic antisense	PELI3
FUT8-AS1	NR_024334	0.032	5.305	0.006	7.483	RefSeq	natural antisense	FUT8
XLOC_014147	TCONS_00029754	0.031	11.706	0.010	7.381	LincRNAs identified by Cabili et al	intergenic	
AC009336.24	ENST00000426615	0.023	12.468	0.004	7.316	GENCODE	intergenic	
RP3-406A7.7	ENST00000564248	0.017	6.050	0.008	7.314	GENCODE	intergenic	
DLEU2	ENST00000425586	0.035	10.251	0.018	7.250	GENCODE	intronic antisense	DLEU1
DLEU2	uc001vdy.3	0.029	10.901	0.012	7.121	UCSC_knowngene	bidirectional	DLEU1
DLEU2	ENST00000438752	0.015	10.559	0.008	7.083	GENCODE	bidirectional	DLEU1
LINC00612	NR_034140	0.016	5.952	0.010	7.060	RefSeq	intergenic	
XLOC_003422	TCONS_00008739	0.025	6.291	0.010	7.038	LincRNAs identified by Cabili et al	intergenic	
XLOC_010235	TCONS_00020965	0.014	9.966	0.007	7.000	LincRNAs identified by Cabili et al	intronic antisense	ZNF664-FAM101A
RP11-157P1.4	ENST00000448371	0.014	8.912	0.006	6.997	GENCODE	intronic antisense	ADRM1
CTD-3065J16.9	ENST00000524499	0.025	5.098	0.011	6.906	GENCODE	natural antisense	EXOSC4
RP11-473M20.7	ENST00000573130	0.014	7.156	0.009	6.897	GENCODE	intronic antisense	MMP25
AC109826.2	ENST00000448595	0.042	5.188	0.013	6.797	GENCODE	intergenic	
AC012146.7	ENST00000413077	0.027	5.919	0.014	6.762	GENCODE	natural antisense	ZNF232
RP11-353N14.2	ENST00000576963	0.044	12.872	0.011	6.633	GENCODE	intergenic	
LINC00173	ENST00000477702	0.018	8.235	0.009	6.609	GENCODE	intergenic	
RP11-160C18.2	ENST00000564933	0.031	5.336	0.031	6.592	pseudogene		
AC017076.5	ENST00000414795	0.030	6.090	0.009	6.535	GENCODE	intergenic	
BC017910	uc003tvu.3	0.036	6.690	0.005	6.419	UCSC_knowngene	intergenic	
	DB503534	0.015	6.578	0.009	6.378	UCR	exon sense-overlapping	ZNF536
RP11-295G20.2	ENST00000450783	0.031	6.426	0.016	6.290	GENCODE	bidirectional	TSNAX
LOC100507564	NR_038953	0.029	5.430	0.011	6.259	RefSeq	natural antisense	LRP8

LOC284889	NR_038911	0.021	8.966	0.014	6.130	RefSeq	natural antisense	MIF
XLOC_014147	TCONS_00029753	0.033	9.216	0.006	6.128	LincRNAs identified by Cabili et al	intergenic	
AC003075.4	ENST00000433005	0.015	5.115	0.002	6.068	GENCODE	natural antisense	AHR
KB-1083B1.1	ENST00000521625	0.044	6.865	0.022	5.800	GENCODE	intronic antisense	SNX31
LOC100128531	NR_038941	0.017	6.631	0.003	5.695	RefSeq	intronic antisense	KIAA1671
RP5-1148A21.3	ENST00000584934	0.038	7.666	0.021	5.694	GENCODE	natural antisense	PTP4A1
FAM86EP	ENST00000510565	0.032	5.763	0.018	5.684	GENCODE	intergenic	
GGT3P	ENST00000453783	0.035	6.036	0.014	5.545	pseudogene		
RP11-381K20.2	ENST00000508281	0.016	6.219	0.006	5.520	GENCODE	natural antisense	MYOT
DCAF8	NR_028105	0.021	8.663	0.004	5.465	RefSeq	exon sense-overlapping	DCAF8
RP4-555L14.5	ENST00000466677	0.031	5.823	0.021	5.434	GENCODE	intronic antisense	WDR86
CTD-2015H6.3	ENST00000504300	0.025	6.380	0.012	5.429	GENCODE	natural antisense	FAM151B
RP11-44N21.1	ENST00000548361	0.047	8.729	0.009	5.419	GENCODE	intergenic	
RP11-245J24.1	ENST00000431695	0.032	7.017	0.004	5.414	GENCODE	intergenic	
RP11-423H2.1	ENST00000504756	0.042	6.105	0.023	5.409	GENCODE	intergenic	
RP11-856B14.1	ENST00000567594	0.021	6.351	0.005	5.308	GENCODE	bidirectional	SSSCA1
BC094873	uc004bxu.3	0.021	5.695	0.021	5.247	UCSC_knowngene	intergenic	
FCGR1B	NR_045213	0.018	5.528	0.004	5.230	RefSeq	exon sense-overlapping	FCGR1B
RP11-267L5.1	ENST00000523682	0.025	5.998	0.010	5.225	GENCODE	intergenic	
LINC00260	NR_046213	0.024	5.045	0.013	5.220	RefSeq	intron sense-overlapping	ATP2B4
RP11-578F21.12	ENST00000563144	0.038	6.410	0.011	5.172	GENCODE	intergenic	
XLOC_002275	TCONS_00004392	0.022	9.107	0.007	5.131	LincRNAs identified by Cabili et al	intergenic	
CTD-3232M19.2	ENST00000528668	0.016	7.828	0.009	5.091	pseudogene		
RP11-62H7.2	ENST00000520582	0.016	8.585	0.004	5.074	GENCODE	intergenic	
RP11-44N21.1	ENST00000548104	0.039	7.622	0.010	5.043	GENCODE	intergenic	
U73167.7	ENST00000421735	0.014	6.345	0.007	5.004	GENCODE	bidirectional	SEMA3B

Table S3. Down-regulated lncRNAs in malignant EOC compared with normal ovary and benign ovarian cyst.

GeneSymbol	Seqname	FDR M Vs. B	Fold Change M Vs. B	FDR M Vs. N	Fold Change M Vs. N	Source	Relationship	Associated_gene
AK094462	uc003jdi.1	0.006	1063.875	0.002	870.191	UCSC_knowngene	intergenic	
AK125532	uc003dmi.1	0.013	636.918	0.006	557.580	UCSC_knowngene	natural antisense	ADAMTS9
XLOC_010769	TCONS_00022431	0.022	680.977	0.012	510.602	LincRNAs identified by Cabili et al	intergenic	
RP11-548O1.3	ENST00000495287	0.011	227.635	0.002	309.756	GENCODE	intergenic	
BC071789	uc021xaa.1	0.016	383.276	0.008	226.659	UCSC_knowngene	intronic antisense	ADAMTS9
ADAMTS9-AS1	ENST00000466225	0.009	352.042	0.002	220.960	GENCODE	natural antisense	ADAMTS9
AC004383.4	ENST00000457876	0.032	48.992	0.014	215.841	GENCODE	intergenic	
MEG3	NR_046466	0.005	41.396	0.002	172.412	RefSeq	intergenic	
XLOC_011177	TCONS_00023867	0.007	188.581	0.002	139.299	LincRNAs identified by Cabili et al	intergenic	
MEG3	ENST00000520714	0.023	33.121	0.010	133.391	GENCODE	intergenic	
ADAMTS9-AS1	ENST00000471990	0.014	180.045	0.003	132.651	GENCODE	intronic antisense	ADAMTS9
RP3-323P13.2	ENST00000419627	0.009	99.855	0.002	121.716	GENCODE	intergenic	
RP11-471J12.1	ENST00000509640	0.018	138.008	0.004	118.482	GENCODE	bidirectional	HAND2
ADAMTS9-AS1	ENST00000493124	0.006	147.583	0.002	112.911	GENCODE	natural antisense	ADAMTS9
RP11-327J17.3	ENST00000560010	0.015	103.105	0.002	111.693	GENCODE	intronic antisense	NR2F2
AL132709.5	ENST00000554369	0.010	37.551	0.002	106.359	GENCODE	intergenic	
MIMT1	NR_024059	0.007	164.450	0.002	105.338	RefSeq	bidirectional	PEG3
RP1-78O14.1	ENST00000550268	0.005	46.148	0.002	102.257	GENCODE	intronic antisense	SYT1
H19	ENST00000417089	0.007	10.734	0.003	100.139	GENCODE	intergenic	
AFAP1-AS1	NR_026892	0.012	331.109	0.004	98.042	RefSeq	natural antisense	AFAP1
GRTP1-AS1	ENST00000423246	0.007	100.786	0.003	93.637	GENCODE	intronic antisense	GRTP1
XLOC_011173	TCONS_00023858	0.014	5.187	0.002	85.263	LincRNAs identified by Cabili et al	intergenic	
RP11-389G6.3	ENST00000562191	0.021	64.381	0.008	82.172	GENCODE	intergenic	
RFPL1-AS1	NR_002727	0.048	152.747	0.038	81.170	RefSeq	natural antisense	RFPL1
NBLA00301	NR_003679	0.019	71.946	0.009	81.111	RefSeq	bidirectional	HAND2
AC092155.4	ENST00000416111	0.023	91.736	0.006	78.352	GENCODE	intergenic	
MEG3	NR_046473	0.006	23.630	0.002	76.960	RefSeq	intergenic	
	DB338868	0.007	60.739	0.002	71.487	LincRNAs identified by Khalil et al	intergenic	
CTC-454M9.1	ENST00000514571	0.031	128.242	0.022	70.668	GENCODE	intergenic	
RP11-471J12.1	ENST00000502941	0.013	47.734	0.005	69.836	GENCODE	bidirectional	HAND2
AL132709.5	ENST00000443252	0.016	14.716	0.004	67.581	GENCODE	intergenic	
TRHDE-AS1	NR_026836	0.012	328.442	0.002	67.128	RefSeq	natural antisense	TRHDE
BX571672.2	ENST00000437267	0.012	12.759	0.002	64.675	GENCODE	intergenic	

RP11-627G18.3	ENST00000583490	0.029	80.191	0.018	62.996	GENCODE	bidirectional	GATA6
MEG3	NR_046464	0.014	15.606	0.002	60.337	RefSeq	intergenic	
RP6-24A23.3	ENST00000436013	0.019	21.163	0.003	57.581	GENCODE	bidirectional	IRS4
	AL049990	0.036	91.355	0.026	54.977	NRED	intergenic	
RP11-471J12.1	ENST00000510339	0.047	29.513	0.025	53.706	GENCODE	bidirectional	HAND2
RP11-548O1.3	ENST00000477059	0.043	54.723	0.028	53.332	GENCODE	intergenic	
RP11-327J17.3	ENST00000560800	0.021	53.335	0.003	53.062	GENCODE	intronic antisense	NR2F2
BC042385	uc001pqc.3	0.043	11.138	0.002	51.134	UCSC_knowngene	intron sense-overlapping	SIK3
RP11-277P12.20	ENST00000500682	0.028	14.321	0.004	50.348	GENCODE	natural antisense	KLRC4-KLRK1
AK123878	uc001yif.1	0.025	19.405	0.002	49.587	UCSC_knowngene	intergenic	
ADAMTS9-AS2	ENST00000474768	0.018	47.778	0.009	47.835	GENCODE	intronic antisense	ADAMTS9
RP11-87H9.3	ENST00000412376	0.017	154.871	0.007	47.066	GENCODE	intergenic	
LINC00607	ENST00000419922	0.024	14.418	0.009	46.326	GENCODE	intergenic	
	AK055244	0.009	71.854	0.005	45.348	NRED	intergenic	
XLOC_011180	TCONS_00023311	0.017	114.106	0.010	45.078	LincRNAs identified by Cabili et al	intergenic	
BC042378	uc003ihb.3	0.007	82.956	0.004	44.971	UCSC_knowngene	intergenic	
BX640700	uc003lqr.3	0.012	15.220	0.004	43.315	UCSC_knowngene	intergenic	
CARKD	NR_040103	0.006	21.731	0.003	42.099	RefSeq	exon sense-overlapping	CARKD
AX747860	uc003qek.1	0.021	22.612	0.003	42.025	UCSC_knowngene	exon sense-overlapping	TCF21
CTC-454M9.1	ENST00000508718	0.021	28.573	0.002	41.954	GENCODE	intronic antisense	MEF2C
H19	ENST00000442037	0.033	6.831	0.011	40.839	GENCODE	intergenic	
HM358988	uc021ybu.1	0.029	9.722	0.008	40.817	UCSC_knowngene	intergenic	
MEG3	ENST00000522618	0.032	6.445	0.010	40.666	GENCODE	intergenic	
LINC00607	NR_037195	0.017	17.880	0.006	39.351	RefSeq	intergenic	
AP000473.5	ENST00000413645	0.028	44.835	0.017	38.342	GENCODE	intergenic	
AL132709.5	ENST00000427085	0.012	19.413	0.004	38.244	GENCODE	intergenic	
ADAMTS9-AS1	ENST00000492209	0.019	29.764	0.009	38.213	GENCODE	natural antisense	ADAMTS9
CTD-2536I1.1	ENST00000556899	0.018	54.670	0.006	38.027	GENCODE	intergenic	
RP11-701H24.4	ENST00000567527	0.016	13.437	0.003	37.880	GENCODE	intergenic	
RP3-332B22.1	ENST00000456896	0.013	37.500	0.004	37.354	GENCODE	intergenic	
XLOC_008182	TCONS_00017068	0.013	42.534	0.004	37.121	LincRNAs identified by Cabili et al	intergenic	
RP1-78O14.1	ENST00000549527	0.009	10.485	0.004	35.910	GENCODE	intronic antisense	SYT1
DQ580140	uc022blc.1	0.005	7.472	0.002	35.304	UCSC_knowngene	intergenic	
LINC00478	ENST00000458468	0.016	32.199	0.004	34.796	GENCODE	intergenic	
XLOC_000281	TCONS_00001016	0.042	56.927	0.032	34.315	LincRNAs identified by Cabili et al	intergenic	
	AF070541	0.007	75.739	0.004	33.709	NRED	intergenic	
MGC16121	NR_024607	0.016	9.848	0.006	32.858	RefSeq	intergenic	
XLOC_011592	TCONS_00023804	0.013	45.793	0.006	32.367	LincRNAs identified by Cabili et al	intergenic	

HM358977	uc021yat.1	0.018	8.623	0.006	31.938	UCSC_knowngene	intronic antisense	TBCA
ZNF300P1	uc003lta.2	0.029	36.289	0.019	31.729	UCSC_knowngene	intergenic	
LINC00340	uc011djj.1	0.015	18.826	0.003	30.754	UCSC_knowngene	intergenic	
LINC00478	ENST00000400178	0.029	31.700	0.016	30.505	GENCODE	intergenic	
FRMD6-AS2	ENST00000557625	0.036	9.112	0.014	29.969	GENCODE	intronic antisense	FRMD6
EDNRA	NR_045958	0.039	41.017	0.028	29.468	RefSeq	exon sense-overlapping	EDNRA
XLOC_006291	TCONS_00013628	0.025	34.139	0.015	27.628	LincRNAs identified by Cabili et al	intergenic	
MAPRE2	NR_046177	0.025	8.959	0.009	27.371	RefSeq	exon sense-overlapping	MAPRE2
FLJ42709	uc010jbb.1	0.038	62.311	0.023	27.314	UCSC_knowngene	intergenic	
RP11-1055B8.4	ENST00000571724	0.022	23.213	0.011	27.294	GENCODE	intergenic	
SOX2-OT	ENST00000476964	0.015	56.047	0.004	27.156	GENCODE	intron sense-overlapping	SOX2
LINC00340	uc003ndk.1	0.019	26.413	0.007	27.118	UCSC_knowngene	intergenic	
AL132709.8	ENST00000442197	0.021	9.908	0.005	27.084	GENCODE	intergenic	
ZNF300P1	ENST00000520773	0.046	30.709	0.032	27.031	GENCODE	intergenic	
PCOLCE-AS1	NR_038910	0.033	45.020	0.023	26.969	RefSeq	natural antisense	FBXO24
RP11-627G18.3	ENST00000584201	0.006	17.975	0.002	26.731	GENCODE	intergenic	
LINC00478	ENST00000418813	0.021	28.466	0.010	26.443	GENCODE	intergenic	
RP11-1101H11.1	ENST00000509363	0.007	77.367	0.002	26.404	GENCODE	intergenic	
C17orf76-AS1	ENST00000578380	0.017	11.283	0.005	26.155	GENCODE	intergenic	
AC018647.3	ENST00000458087	0.017	12.897	0.008	25.709	GENCODE	intergenic	
CTC-454M9.1	ENST00000508742	0.021	25.325	0.011	25.568	GENCODE	intergenic	
RP11-296E23.2	ENST00000584331	0.033	21.323	0.018	25.440	GENCODE	intron sense-overlapping	GREB1L
RP11-627G18.3	ENST00000584373	0.018	21.412	0.006	25.281	GENCODE	intergenic	
AK025321	uc003tdv.1	0.038	41.998	0.029	25.048	UCSC_knowngene	intergenic	
LINC00478	ENST00000453910	0.027	17.584	0.006	24.551	GENCODE	intergenic	
CTD-3076O17.1	ENST00000528696	0.022	44.447	0.015	24.124	GENCODE	natural antisense	ADAMTS17
RP1-146A15.1	ENST00000424251	0.031	64.006	0.011	24.032	GENCODE	intron sense-overlapping	IL1RAPL1
RP11-471J12.1	ENST00000515376	0.016	39.346	0.002	24.009	GENCODE	bidirectional	HAND2
CTC-454M9.1	ENST00000511876	0.036	23.229	0.021	23.964	GENCODE	natural antisense	MEF2C
FLJ42709	uc003kkg.2	0.032	34.922	0.022	23.943	UCSC_knowngene	intergenic	
CTD-2104P17.1	ENST00000452153	0.022	11.477	0.007	23.897	GENCODE	intergenic	
RP11-212D3.3	ENST00000431331	0.017	74.895	0.008	23.810	GENCODE	intergenic	
RP11-693N9.2	ENST00000532510	0.021	59.419	0.004	23.564	GENCODE	intergenic	
RP11-475B2.1	ENST00000561655	0.007	20.863	0.003	23.513	GENCODE	intergenic	
RP11-74M11.2	ENST00000506195	0.028	9.426	0.004	23.335	GENCODE	intergenic	
RP11-999E24.3	ENST00000563647	0.020	24.489	0.005	22.982	GENCODE	intergenic	
RP11-522B15.3	ENST00000558382	0.031	33.977	0.021	22.785	GENCODE	intergenic	
RP11-548O1.3	ENST00000483650	0.009	27.890	0.004	22.764	GENCODE	intergenic	

XLOC_013547	TCONS_00028196	0.016	7.431	0.005	22.575	LincRNAs identified by Cabili et al	intergenic	
AX746867	uc003yjz.1	0.020	6.292	0.002	22.490	UCSC_knowngene	intron sense-overlapping	YWHAZ
MEG3	ENST00000398460	0.022	7.794	0.003	22.428	GENCODE	intergenic	
LOC100132741	NR_034004	0.030	38.337	0.023	21.265	RefSeq	intergenic	
AC107982.4	ENST00000507171	0.020	47.715	0.002	21.026	pseudogene		
RP11-597D13.9	ENST00000503611	0.025	28.870	0.017	20.515	GENCODE	natural antisense	FAM198B
LEMD1-AS1	NR_038425	0.015	42.130	0.007	20.514	RefSeq	intronic antisense	LEMD1
LOC100507387	NR_038402	0.019	9.833	0.005	20.295	RefSeq	intergenic	
XLOC_012171	TCONS_00025893	0.019	23.673	0.003	20.185	LincRNAs identified by Cabili et al	exon sense-overlapping	TMEM98
	CB112975	0.023	5.807	0.004	20.147	LincRNAs identified by Khalil et al	intergenic	
GTF2IRD2P1	ENST00000425256	0.017	12.365	0.008	20.135	GENCODE	intergenic	
LOC645434	NR_033919	0.014	20.844	0.004	19.835	RefSeq	intergenic	
BC037304	uc001djh.1	0.019	13.163	0.004	19.664	UCSC_knowngene	exon sense-overlapping	PRKACB
LOC386758	NR_037159	0.009	38.618	0.004	19.612	RefSeq	bidirectional	ZNF582
RP11-689P11.2	ENST00000382488	0.016	10.074	0.003	19.347	GENCODE	intergenic	
AK021977	uc001uxg.3	0.009	26.174	0.010	19.287	UCSC_knowngene	intron sense-overlapping	LHFP
LOC100302640	uc003dwf.4	0.036	21.629	0.024	19.151	UCSC_knowngene	intergenic	
RP11-524C21.2	ENST00000567753	0.006	18.482	0.002	19.116	GENCODE	intergenic	
MAGI2-AS3	NR_038343	0.014	63.751	0.004	19.039	RefSeq	bidirectional	MAGI2
KGFLP1	uc011lrd.2	0.012	28.357	0.007	19.018	UCSC_knowngene	intergenic	
RP11-471J12.1	ENST00000507571	0.016	11.568	0.004	18.978	GENCODE	bidirectional	HAND2
XLOC_002344	TCONS_00004467	0.023	16.938	0.012	18.924	LincRNAs identified by Cabili et al	intergenic	
RP11-296A18.6	ENST00000422306	0.022	12.372	0.003	18.868	GENCODE	intron sense-overlapping	RNF11
AK027091	uc010wia.1	0.016	10.124	0.006	18.816	UCSC_knowngene	intergenic	
CRB1	NR_047564	0.006	12.292	0.008	18.586	RefSeq	exon sense-overlapping	CRB1
MEG3	ENST00000521256	0.014	7.230	0.004	18.548	GENCODE	intergenic	
CTD-2576F9.2	ENST00000565166	0.031	10.734	0.015	18.497	GENCODE	intergenic	
RP11-471J12.1	ENST00000505817	0.010	17.126	0.003	18.338	GENCODE	bidirectional	HAND2
AX748340	uc002vja.1	0.012	33.152	0.003	18.121	UCSC_knowngene	natural antisense	PRKAG3
RP11-389O22.1	ENST00000451149	0.028	13.705	0.011	18.046	GENCODE	intergenic	
LINC00311	ENST00000366314	0.038	9.334	0.018	17.986	GENCODE	intergenic	
BC015383	uc002nrq.3	0.012	86.123	0.002	17.893	UCSC_knowngene	intergenic	
DNM3OS	NR_038397	0.032	12.792	0.011	17.878	RefSeq	intronic antisense	DNM3
FLJ42709	uc003kkf.2	0.013	25.207	0.004	17.874	UCSC_knowngene	intergenic	
LOC643733	uc001phz.3	0.021	29.762	0.012	17.802	UCSC_knowngene	intergenic	
C17orf76-AS1	NR_045021	0.012	9.852	0.004	17.797	RefSeq	natural antisense	FAM211A
AC084117.3	ENST00000496975	0.023	7.913	0.009	17.756	GENCODE	intron sense-overlapping	LDHA
RP11-1299A16.3	ENST00000569927	0.007	29.959	0.003	17.749	GENCODE	bidirectional	TSPAN5

RP5-1007M22.2	ENST00000526694	0.047	16.723	0.025	17.506	GENCODE	bidirectional	LRRC8C
CTC-462L7.1	ENST00000565472	0.024	40.054	0.018	17.492	GENCODE	intergenic	
RP11-497E19.1	ENST00000380722	0.017	24.967	0.008	17.380	GENCODE	natural antisense	RP11-497E19.2
RP11-13L2.4	ENST00000564660	0.016	17.346	0.006	17.302	GENCODE	intergenic	
AF116693	uc003xdr.1	0.024	8.825	0.009	17.258	UCSC_knowngene	exon sense-overlapping	SLC25A37
	BQ950928	0.021	14.402	0.002	17.219	LincRNAs identified by Khalil et al	intergenic	
XLOC_010128	TCONS_00020843	0.022	34.713	0.016	17.196	LincRNAs identified by Cabili et al	intergenic	
LINC00478	ENST00000435697	0.016	13.448	0.006	17.190	GENCODE	intergenic	
RP11-486A14.2	ENST00000549806	0.014	35.038	0.004	17.039	GENCODE	bidirectional	NUDT4
XLOC_002368	TCONS_00005259	0.013	16.936	0.006	16.978	LincRNAs identified by Cabili et al	intergenic	
RP1-251M9.2	ENST00000420825	0.023	11.223	0.004	16.976	GENCODE	intergenic	
RP11-122C5.1	ENST00000506463	0.026	20.879	0.012	16.813	GENCODE	intergenic	
TP73-AS1	NR_033711	0.035	9.013	0.017	16.474	RefSeq	natural antisense	TP73
C17orf76-AS1	NR_027158	0.011	10.662	0.003	16.468	RefSeq	natural antisense	FAM211A
XLOC_004271	TCONS_00009859	0.017	14.630	0.008	16.365	LincRNAs identified by Cabili et al	intergenic	
C17orf76-AS1	NR_027162	0.022	9.783	0.004	16.347	RefSeq	natural antisense	FAM211A
AL132709.8	ENST00000554693	0.021	7.187	0.003	16.147	GENCODE	intergenic	
CEP170P1	ENST00000502249	0.017	5.809	0.003	16.056	pseudogene		
RP11-399E6.4	ENST00000429109	0.018	25.534	0.002	15.897	GENCODE	intronic antisense	FOXO6
RP11-556O5.5	ENST00000519726	0.050	10.670	0.025	15.868	GENCODE	intergenic	
RP1-283K11.3	ENST00000412954	0.033	15.416	0.014	15.840	GENCODE	intronic antisense	EYA4
XLOC_002368	TCONS_00005258	0.032	14.807	0.002	15.805	LincRNAs identified by Cabili et al	intergenic	
XLOC_010933	TCONS_00022632	0.030	8.330	0.004	15.581	LincRNAs identified by Cabili et al	intergenic	
LOC255167	NR_024423	0.019	8.436	0.004	15.538	RefSeq	intergenic	
SNHG5	uc003plb.4	0.013	13.053	0.004	15.394	UCSC_knowngene	intergenic	
RP11-826N14.4	ENST00000510029	0.016	14.743	0.006	15.126	GENCODE	intergenic	
LINC00478	ENST00000419952	0.017	10.135	0.004	15.100	GENCODE	intergenic	
RP11-75C9.1	ENST00000447950	0.006	15.591	0.006	15.052	GENCODE	intronic antisense	PTPRD
RP11-471J12.1	ENST00000512246	0.014	7.136	0.004	15.029	GENCODE	bidirectional	HAND2
LOC145820	NR_027133	0.014	13.564	0.004	15.004	RefSeq	intergenic	
RP11-65F13.2	ENST00000513055	0.019	25.745	0.012	14.914	GENCODE	natural antisense	NR2F1
LINC00607	ENST00000413563	0.016	6.816	0.005	14.883	GENCODE	intergenic	
XLOC_000057	TCONS_00000842	0.025	25.362	0.014	14.880	LincRNAs identified by Cabili et al	intergenic	
LOC100506474	NR_038434	0.021	16.691	0.012	14.848	RefSeq	intergenic	
UBE2Q2P1	ENST00000561403	0.029	6.060	0.011	14.841	GENCODE	intergenic	
LOC100505854	uc003ugj.1	0.015	13.667	0.002	14.834	UCSC_knowngene	natural antisense	RSBN1L
XLOC_005101	TCONS_00010578	0.021	21.837	0.004	14.817	LincRNAs identified by Cabili et al	intergenic	
RP11-446H18.3	ENST00000463143	0.033	12.040	0.018	14.733	GENCODE	intergenic	

AP001468.58	ENST00000415026	0.031	14.080	0.019	14.716	GENCODE	natural antisense	LSS
RP11-88L24.4	ENST00000582038	0.033	71.521	0.030	14.535	GENCODE	natural antisense	TTN
RP11-284N8.3	ENST00000566942	0.015	19.148	0.002	14.491	GENCODE	intergenic	
RP11-471J12.1	ENST00000503309	0.012	9.025	0.004	14.485	GENCODE	bidirectional	HAND2
RP11-53O19.1	ENST00000503452	0.013	18.956	0.018	14.454	GENCODE	bidirectional	MRPS30
LOC145820	NR_027132	0.007	14.042	0.003	14.404	RefSeq	intergenic	
CTC-459I6.1	ENST00000505694	0.029	66.738	0.028	14.273	GENCODE	natural antisense	RASGRF2
RP11-471J12.1	ENST00000508887	0.043	9.707	0.003	14.241	GENCODE	bidirectional	HAND2
RP11-471J12.1	ENST00000507636	0.017	31.604	0.006	14.033	GENCODE	bidirectional	HAND2
MEG8	ENST00000556475	0.050	9.787	0.002	14.007	GENCODE	intergenic	
PWRN1	ENST00000565512	0.007	103.648	0.007	14.003	GENCODE	intergenic	
PWRN1	ENST00000568019	0.012	75.605	0.009	13.992	GENCODE	intergenic	
XLOC_011182	TCONS_00023877	0.006	35.343	0.005	13.990	LincRNAs identified by Cabili et al	intergenic	
RP4-794H19.4	ENST00000447329	0.018	44.840	0.013	13.970	GENCODE	intergenic	
JF824130	uc021xxo.1	0.019	23.632	0.002	13.960	UCSC_knowngene	bidirectional	GDNF
DNM3OS	ENST00000417354	0.032	25.213	0.017	13.956	GENCODE	intronic antisense	DNM3
BC044655	uc002gwy.3	0.014	49.858	0.010	13.920	UCSC_knowngene	intergenic	
DKFZp666K117	uc001utv.3	0.036	21.857	0.026	13.899	UCSC_knowngene	intergenic	
DAPK1-IT1	ENST00000431813	0.035	9.140	0.014	13.888	GENCODE	intron sense-overlapping	DAPK1
MAGI2-AS3	ENST00000442765	0.012	50.630	0.007	13.885	GENCODE	intergenic	
RP11-752D24.2	ENST00000504048	0.040	5.315	0.003	13.858	GENCODE	intronic antisense	SCFD2
AC087886.1	ENST00000550334	0.023	23.448	0.018	13.842	GENCODE	bidirectional	TRHDE
RP11-264F23.3	ENST00000539135	0.010	52.257	0.004	13.812	GENCODE	natural antisense	CCND2
AX748314	uc002frx.1	0.043	6.882	0.002	13.803	UCSC_knowngene	intergenic	
AL078621.4	ENST00000433106	0.014	14.977	0.002	13.733	GENCODE	intergenic	
C17orf76-AS1	ENST00000484836	0.013	9.371	0.005	13.693	GENCODE	intergenic	
LINC00622	NR_036540	0.042	18.980	0.031	13.690	RefSeq	intergenic	
GDNF-AS1	ENST00000510986	0.010	14.131	0.002	13.605	GENCODE	intergenic	
FLJ42709	uc003kke.2	0.018	20.122	0.002	13.553	UCSC_knowngene	intergenic	
RP11-597D13.9	ENST00000509463	0.012	20.869	0.004	13.539	GENCODE	natural antisense	FAM198B
LINC00340	NR_015410	0.016	9.924	0.002	13.532	RefSeq	intergenic	
RP11-82O18.2	ENST00000568317	0.021	13.368	0.011	13.511	GENCODE	intergenic	
LOC148824	NR_027309	0.007	219.738	0.004	13.436	RefSeq	exon sense-overlapping	OR2C3
SNHG5	ENST00000420199	0.018	10.903	0.008	13.189	GENCODE	intergenic	
RP5-1050E16.2	ENST00000421297	0.018	41.281	0.012	13.118	GENCODE	intergenic	
C17orf76-AS1	ENST00000483140	0.010	8.094	0.004	13.042	GENCODE	natural antisense	FAM211A
RP11-396F22.1	ENST00000551152	0.032	17.194	0.022	12.999	GENCODE	intronic antisense	CPNE8
PWRN1	ENST00000562501	0.005	30.915	0.007	12.820	GENCODE	intergenic	

XLOC_006719	TCONS_00015224	0.025	5.277	0.009	12.814	LincRNAs identified by Cabili et al	intergenic	
RP11-345M22.1	ENST00000561510	0.022	14.288	0.004	12.530	GENCODE	intergenic	
MIR143HG	NR_027180	0.021	15.821	0.006	12.527	RefSeq	intergenic	
C17orf76-AS1	NR_045025	0.010	8.293	0.004	12.452	RefSeq	intronic antisense	FAM211A
USP3	NR_046341	0.022	9.489	0.007	12.227	RefSeq	exon sense-overlapping	USP3
XLOC_012171	TCONS_00025350	0.016	14.506	0.003	12.215	LincRNAs identified by Cabili et al	intergenic	
MAGI2-AS3	ENST00000448195	0.036	20.454	0.025	12.165	GENCODE	bidirectional	MAGI2
AK130904	uc004ags.1	0.018	29.777	0.009	12.132	UCSC_knowngene	natural antisense	TMEM252
AC123886.2	ENST00000458252	0.012	30.900	0.006	12.118	GENCODE	intergenic	
RP11-493L12.4	ENST00000547777	0.019	32.613	0.005	12.056	GENCODE	intergenic	
RP11-446H18.3	ENST00000495228	0.020	13.230	0.009	12.041	GENCODE	intergenic	
TUBA3FP	NR_003608	0.025	25.741	0.018	11.943	RefSeq	bidirectional	P2RX6
RP1-28O10.1	ENST00000445272	0.022	8.690	0.011	11.863	GENCODE	bidirectional	G0S2
MEG8	ENST00000553465	0.007	7.658	0.007	11.846	GENCODE	intergenic	
C17orf76-AS1	NR_027171	0.017	6.474	0.004	11.739	RefSeq	intronic antisense	FAM211A
DQ598910	uc002djt.2	0.023	7.341	0.003	11.680	UCSC_knowngene	exon sense-overlapping	RP11-645C24.1
GUSBP11	uc002zxh.4	0.031	8.026	0.006	11.617	UCSC_knowngene	natural antisense	RGL4
ARMCX4	NR_045864	0.020	11.187	0.002	11.555	RefSeq	exon sense-overlapping	ARMCX4
XLOC_012171	TCONS_00025894	0.042	10.279	0.024	11.524	LincRNAs identified by Cabili et al	exon sense-overlapping	TMEM98
AC018865.8	ENST00000456785	0.040	29.399	0.039	11.466	GENCODE	intergenic	
XLOC_011172	TCONS_00023857	0.017	48.931	0.007	11.452	LincRNAs identified by Cabili et al	intergenic	
LOC375196	NR_028386	0.029	18.984	0.018	11.412	RefSeq	natural antisense	ARHGEF33
RPL23AP53	uc010lrb.3	0.036	5.728	0.016	11.383	UCSC_knowngene	natural antisense	ZNF596
MAGI2-AS3	ENST00000452320	0.025	14.164	0.016	11.052	GENCODE	intergenic	
LOC100134317	NR_029389	0.018	12.145	0.009	11.028	RefSeq	intergenic	
BC035125	uc002sem.1	0.017	17.933	0.007	11.013	UCSC_knowngene	exon sense-overlapping	CNRIP1
FAM13A-AS1	uc003hrz.1	0.007	18.357	0.007	10.907	UCSC_knowngene	natural antisense	FAM13A
RP11-770E5.3	ENST00000521660	0.014	16.584	0.015	10.782	GENCODE	intergenic	
PRH1-PRR4	NR_037918	0.017	9.789	0.002	10.648	RefSeq	exon sense-overlapping	PRR4
ASH2L	NR_049736	0.029	7.915	0.015	10.647	RefSeq	exon sense-overlapping	ASH2L
RP11-291L15.2	ENST00000503066	0.045	5.052	0.018	10.642	GENCODE	bidirectional	HHIP
RP11-347H15.5	ENST00000531666	0.026	11.898	0.013	10.547	pseudogene		
NT5C3L	NR_033465	0.023	6.883	0.007	10.473	RefSeq	exon sense-overlapping	NT5C3L
RP11-275N1.1	ENST00000433460	0.022	12.801	0.004	10.409	GENCODE	intergenic	
DKFZP434L187	NR_026771	0.014	21.647	0.004	10.373	RefSeq	intergenic	
CTD-3092A11.1	ENST00000568690	0.016	15.120	0.004	10.275	GENCODE	intergenic	
LINC00478	ENST00000428669	0.025	7.028	0.008	10.203	GENCODE	intergenic	
U50535	uc001uum.3	0.043	8.233	0.025	9.892	UCSC_knowngene	exon sense-overlapping	N4BP2L2

LMAN2L	NR_024519	0.028	9.828	0.018	9.880	RefSeq	exon sense-overlapping	LMAN2L
CTD-2195M18.1	ENST00000507628	0.022	6.153	0.009	9.832	GENCODE	intergenic	
AL592284.1	ENST00000414344	0.023	13.203	0.013	9.752	GENCODE	natural antisense	AL592284.2
RP11-553L6.5	ENST00000570269	0.013	11.086	0.007	9.725	GENCODE	intergenic	
UBE2Q2P1	ENST00000339094	0.021	10.271	0.008	9.719	GENCODE	intergenic	
PLEKHA8P1	ENST00000336399	0.032	11.606	0.005	9.673	pseudogene		
XLOC_013054	TCONS_00027009	0.011	12.009	0.007	9.607	LincRNAs identified by Cabili et al	intergenic	
MAGI2-AS3	NR_038346	0.043	9.109	0.023	9.574	RefSeq	natural antisense	MAGI2
FHL1	NR_027621	0.009	10.178	0.004	9.558	RefSeq	exon sense-overlapping	FHL1
XLOC_004165	TCONS_00008647	0.009	15.090	0.004	9.557	LincRNAs identified by Cabili et al	intergenic	
AC016910.1	ENST00000422799	0.017	23.415	0.014	9.508	GENCODE	intronic antisense	GTDC1
C17orf76-AS1	ENST00000480811	0.020	5.381	0.009	9.504	GENCODE	intergenic	
RP11-471J12.1	ENST00000507322	0.016	7.182	0.010	9.497	GENCODE	bidirectional	HAND2
XLOC_010061	TCONS_00021307	0.020	13.440	0.011	9.492	LincRNAs identified by Cabili et al	intergenic	
AC067956.1	ENST00000414512	0.030	7.425	0.003	9.482	GENCODE	intergenic	
	BU594464	0.037	7.787	0.021	9.477	LincRNAs identified by Khalil et al	exon sense-overlapping	ANXA5
AC092667.2	ENST00000434301	0.046	16.223	0.039	9.468	GENCODE	natural antisense	AFF3
RP11-325I22.2	ENST00000504989	0.019	22.030	0.011	9.441	GENCODE	intergenic	
RP11-71E19.2	ENST00000514281	0.015	7.692	0.009	9.421	GENCODE	intergenic	
C17orf76-AS1	NR_027165	0.025	5.145	0.006	9.358	RefSeq	natural antisense	FAM211A
RP11-429G19.2	ENST00000458228	0.012	7.512	0.003	9.322	GENCODE	intronic antisense	CCNJ
CTD-2350C19.1	ENST00000583956	0.018	14.251	0.004	9.270	GENCODE	intronic antisense	SLC46A1
ARMCX4	NR_045861	0.018	9.587	0.007	9.257	RefSeq	exon sense-overlapping	ARMCX4
C17orf76-AS1	ENST00000472293	0.021	7.884	0.008	9.257	GENCODE	natural antisense	FAM211A
RP11-38P22.2	ENST00000460407	0.037	20.481	0.005	9.132	GENCODE	intergenic	
AC092155.4	ENST00000444672	0.011	8.876	0.004	9.129	GENCODE	intergenic	
MAGI2-AS3	ENST00000435749	0.021	6.771	0.004	9.099	GENCODE	intergenic	
CTD-2576F9.2	ENST00000564371	0.032	5.276	0.012	9.099	GENCODE	intergenic	
RP11-693N9.2	ENST00000527425	0.014	22.929	0.005	9.060	GENCODE	intergenic	
RP11-99L13.2	ENST00000557506	0.013	70.003	0.006	9.052	GENCODE	intergenic	
IGL@	uc022cjh.1	0.033	9.369	0.015	9.033	UCSC_knowngene	intergenic	
AK124130	uc010ixm.1	0.032	7.000	0.006	8.957	UCSC_knowngene	intergenic	
ATF2	NR_045769	0.045	5.471	0.002	8.953	RefSeq	exon sense-overlapping	ATF2
AK096159	uc004afb.1	0.025	19.069	0.019	8.946	UCSC_knowngene	intergenic	
RP11-319G6.1	ENST00000515247	0.013	22.380	0.002	8.906	GENCODE	intronic antisense	RBP1
LOC100505783	NR_038338	0.025	10.223	0.013	8.901	RefSeq	bidirectional	C20orf111
RP11-101P17.11	ENST00000453309	0.037	5.225	0.002	8.825	GENCODE	intergenic	
RP4-717I23.3	ENST00000445076	0.037	6.022	0.015	8.776	GENCODE	bidirectional	DR1

FLJ42709	uc010jbc.2	0.027	7.525	0.008	8.773	UCSC_knowngene	intergenic	
LINC00340	uc010jpp.1	0.010	10.036	0.010	8.752	UCSC_knowngene	intergenic	
CES5AP1	ENST00000431237	0.026	31.339	0.025	8.744	GENCODE	intergenic	
RP4-561L24.3	ENST00000565336	0.026	7.168	0.012	8.721	GENCODE	bidirectional	BCAR3
MIR181A2HG	ENST00000429139	0.014	19.215	0.009	8.697	GENCODE	intronic antisense	NR6A1
XLOC_012169	TCONS_00025349	0.019	8.176	0.007	8.633	LincRNAs identified by Cabili et al	intergenic	
AC091878.1	ENST00000538380	0.040	11.310	0.025	8.595	GENCODE	bidirectional	BASP1
TP73-AS1	NR_033710	0.025	8.373	0.016	8.594	RefSeq	natural antisense	TP73
AC058791.2	ENST00000435523	0.020	10.939	0.011	8.591	GENCODE	intergenic	
FLJ43663	NR_024153	0.040	8.398	0.016	8.583	RefSeq	intergenic	
RP11-642D21.1	ENST00000519805	0.028	6.142	0.011	8.524	GENCODE	intron sense-overlapping	ZFPM2
XLOC_000915	TCONS_00001581	0.018	11.876	0.010	8.451	LincRNAs identified by Cabili et al	intergenic	
RP4-584D14.7	ENST00000563946	0.041	5.486	0.021	8.424	GENCODE	bidirectional	RARRES2
RP11-305L7.6	ENST00000437389	0.035	23.309	0.034	8.387	GENCODE	intergenic	
LA16c-444G7.2	ENST00000569362	0.016	33.222	0.010	8.342	GENCODE	intergenic	
RP11-204M4.2	ENST00000454645	0.013	5.863	0.006	8.328	GENCODE	intergenic	
RP11-261C10.3	ENST00000431528	0.029	7.953	0.006	8.281	GENCODE	intergenic	
LINC00484	ENST00000423719	0.021	12.390	0.014	8.275	GENCODE	intergenic	
LOC643837	NR_047520	0.032	7.665	0.018	8.264	RefSeq	intergenic	
BC067268	uc021vhp.1	0.024	8.505	0.006	8.253	UCSC_knowngene	exon sense-overlapping	SPTBN1
LOC339524	NR_026987	0.028	7.386	0.016	8.153	RefSeq	exon sense-overlapping	HS2ST1
TPT1-AS1	ENST00000517509	0.012	6.258	0.004	8.133	GENCODE	natural antisense	TPT1
LOC147976	ENST00000443870	0.015	15.516	0.003	8.093	GENCODE	intergenic	
COX10-AS1	NR_049718	0.036	8.517	0.003	8.008	RefSeq	natural antisense	COX10
BBS4	NR_045565	0.007	9.803	0.006	7.976	RefSeq	exon sense-overlapping	BBS4
UG0898H09	NR_027378	0.016	17.515	0.013	7.966	RefSeq	intron sense-overlapping	NKAIN3
RP11-74M13.4	ENST00000557452	0.025	18.553	0.020	7.900	GENCODE	intergenic	
AL390170	uc004bdv.3	0.016	17.634	0.007	7.870	UCSC_knowngene	intergenic	
RP11-280O1.2	ENST00000415000	0.045	22.415	0.009	7.868	GENCODE	intergenic	
AC156455.1	ENST00000535337	0.026	14.487	0.020	7.828	GENCODE	intergenic	
RP11-151D14.1	ENST00000449235	0.044	5.320	0.016	7.787	GENCODE	intergenic	
CTC-454M9.1	ENST00000509179	0.022	10.349	0.015	7.756	GENCODE	intronic antisense	MEF2C
SNHG12	ENST00000464115	0.007	6.395	0.004	7.700	GENCODE	intergenic	
RP11-1008C21.2	ENST00000559232	0.024	13.184	0.009	7.692	GENCODE	intergenic	
RP4-798C17.6	ENST00000475089	0.042	9.958	0.023	7.674	GENCODE	intronic antisense	OR2A42
AC156455.1	ENST00000538790	0.031	10.681	0.019	7.640	GENCODE	intergenic	
AL592284.1	ENST00000429496	0.016	5.752	0.008	7.638	GENCODE	intronic antisense	AL592284.2
THSD1P1	uc001vgk.2	0.032	6.170	0.005	7.594	UCSC_knowngene	intergenic	

RP11-61A14.1	ENST00000551187	0.012	11.329	0.006	7.592	GENCODE	natural antisense	CA7
RP11-125B21.2	ENST00000416826	0.014	22.805	0.004	7.591	GENCODE	bidirectional	VLDLR
DQ580140	uc022bdg.1	0.020	8.387	0.011	7.591	UCSC_knowngene	intron sense-overlapping	KDM4C
RP11-30L4.5	ENST00000434312	0.042	6.941	0.026	7.556	GENCODE	intron sense-overlapping	FAM120A
RP11-304F15.7	ENST00000574365	0.026	9.992	0.017	7.532	GENCODE	intergenic	
AX721199	uc001yab.3	0.010	16.249	0.004	7.525	UCSC_knowngene	intergenic	
GOLGA8A	NR_027409	0.020	7.949	0.007	7.520	RefSeq	exon sense-overlapping	GOLGA8A
ID2B	uc011bfh.2	0.024	24.077	0.004	7.519	UCSC_knowngene	intronic antisense	PTPRG
ADAMTS9-AS2	ENST00000460833	0.021	10.059	0.002	7.504	GENCODE	intronic antisense	ADAMTS9
UBE2Q2P1	ENST00000559010	0.022	5.139	0.002	7.435	GENCODE	intergenic	
BC022047	uc003pyw.1	0.029	9.095	0.002	7.428	UCSC_knowngene	intergenic	
RP11-119D9.1	ENST00000533670	0.014	15.293	0.004	7.417	GENCODE	intergenic	
NSUN2	NR_037947	0.043	5.529	0.022	7.404	RefSeq	exon sense-overlapping	NSUN2
RP11-75C9.1	ENST00000430766	0.016	7.694	0.003	7.401	GENCODE	intronic antisense	PTPRD
XLOC_012171	TCONS_00025895	0.033	9.981	0.007	7.398	LincRNAs identified by Cabili et al	intergenic	
LOC643837	NR_047522	0.046	7.116	0.027	7.379	RefSeq	intergenic	
AC010090.1	ENST00000413525	0.007	11.863	0.008	7.350	GENCODE	intergenic	
RP11-426C22.5	ENST00000562902	0.047	12.199	0.041	7.343	GENCODE	intergenic	
RP11-99L13.2	ENST00000557721	0.021	62.594	0.025	7.322	GENCODE	intergenic	
TRAF3IP2-AS1	NR_034108	0.013	9.346	0.007	7.307	RefSeq	intronic antisense	TRAF3IP2
CTC-454M9.1	ENST00000512585	0.014	10.443	0.006	7.290	GENCODE	intronic antisense	MEF2C
RP11-464O2.2	ENST00000432699	0.017	20.787	0.002	7.290	GENCODE	intronic antisense	FAM53B
XLOC_011753	TCONS_00024436	0.006	15.917	0.012	7.249	LincRNAs identified by Cabili et al	intergenic	
LOC646626	NR_045484	0.014	7.336	0.004	7.227	RefSeq	natural antisense	BCL10
XLOC_003200	TCONS_00007221	0.016	10.065	0.005	7.206	LincRNAs identified by Cabili et al	intergenic	
RP11-250B2.5	ENST00000569267	0.007	14.515	0.007	7.165	GENCODE	intergenic	
RP11-3N2.1	ENST00000430271	0.018	42.271	0.011	7.147	GENCODE	intergenic	
	AK095276	0.021	12.910	0.014	7.144	LincRNAs identified by Khalil et al	intergenic	
RP11-274H2.5	ENST00000567714	0.030	7.269	0.006	7.132	GENCODE	intergenic	
CTD-3092A11.1	ENST00000399971	0.021	5.272	0.007	7.115	pseudogene		
LGALS3	NR_003225	0.014	15.874	0.023	7.088	RefSeq	exon sense-overlapping	LGALS3
SKINTL	ENST00000452503	0.013	13.777	0.007	7.088	GENCODE	intergenic	
AF268386	uc001gch.1	0.050	6.478	0.006	7.071	UCSC_knowngene	intergenic	
AC058791.2	ENST00000429901	0.021	7.407	0.008	7.070	GENCODE	intergenic	
LINC00629	NR_038998	0.012	7.521	0.006	7.057	RefSeq	intergenic	
XLOC_002710	TCONS_00006894	0.021	5.433	0.008	7.033	LincRNAs identified by Cabili et al	intergenic	
TPT1-AS1	ENST00000379050	0.025	7.219	0.015	6.941	GENCODE	intergenic	
XLOC_011112	TCONS_00022845	0.031	10.449	0.020	6.940	LincRNAs identified by Cabili et al	intergenic	

PI4KAP1	uc010gsf.1	0.027	7.227	0.017	6.915	UCSC_knowngene	intergenic	
XLOC_009734	TCONS_00020406	0.032	12.353	0.027	6.908	LincRNAs identified by Cabili et al	intergenic	
RP11-704J17.5	ENST00000451017	0.039	6.327	0.023	6.903	GENCODE	natural antisense	EYA4
RP4-756H11.3	ENST00000439360	0.022	5.915	0.007	6.877	GENCODE	intergenic	
RP11-327J17.3	ENST00000561402	0.008	13.371	0.011	6.871	GENCODE	intergenic	
PCDHB18	ENST00000526308	0.029	8.230	0.019	6.834	GENCODE	intergenic	
RP11-736K20.5	ENST00000528660	0.024	8.054	0.015	6.809	GENCODE	intronic antisense	PRSS23
TP73-AS1	NR_033708	0.006	5.719	0.007	6.807	RefSeq	natural antisense	TP73
CCDC144C	ENST00000580574	0.021	18.859	0.016	6.807	GENCODE	intergenic	
RP11-788M5.3	ENST00000526107	0.010	6.209	0.006	6.801	pseudogene		
AF157115	uc003lvl.3	0.033	6.289	0.004	6.785	UCSC_knowngene	exon sense-overlapping	SAP30L
RP11-815I9.4	ENST00000578214	0.037	5.422	0.019	6.769	GENCODE	intron sense-overlapping	ZNF286B
RP5-919F19.5	ENST00000423747	0.012	5.456	0.004	6.757	GENCODE	natural antisense	AKD1
WDFY3-AS2	NR_015359	0.048	5.058	0.008	6.734	RefSeq	bidirectional	WDFY3
LOC654342	uc002sts.4	0.019	11.155	0.011	6.726	UCSC_knowngene	intergenic	
LOC386758	NR_037160	0.012	30.846	0.020	6.705	RefSeq	bidirectional	ZNF582
LINC00266-4P	ENST00000427373	0.021	9.870	0.006	6.637	GENCODE	intergenic	
RP11-597D13.9	ENST00000508752	0.037	6.298	0.023	6.618	GENCODE	intergenic	
LOC387647	uc001iup.3	0.012	11.761	0.006	6.573	UCSC_knowngene	natural antisense	SVIL
LOC100190939	uc010tfr.2	0.008	8.306	0.004	6.573	UCSC_knowngene	natural antisense	TPT1
RP11-495P10.2	ENST00000417554	0.041	8.297	0.008	6.556	GENCODE	intergenic	
RP11-182I10.3	ENST00000415842	0.024	6.491	0.003	6.551	GENCODE	intergenic	
XLOC_011176	TCONS_00023866	0.006	62.623	0.008	6.529	LincRNAs identified by Cabili et al	intergenic	
BX284650.1	ENST00000440862	0.022	10.864	0.011	6.524	GENCODE	intergenic	
AY343891	uc002tjx.4	0.021	11.079	0.012	6.499	UCSC_knowngene	intergenic	
BX284650.1	ENST00000429388	0.007	10.492	0.004	6.453	GENCODE	intergenic	
XLOC_008379	TCONS_00018118	0.030	17.319	0.027	6.450	LincRNAs identified by Cabili et al	intergenic	
RP11-305O6.3	ENST00000545709	0.036	10.808	0.027	6.449	GENCODE	intron sense-overlapping	MSRB3
GSTM4	NR_024538	0.020	15.448	0.014	6.411	RefSeq	exon sense-overlapping	GSTM4
RP11-844P9.1	ENST00000504629	0.033	17.853	0.027	6.355	GENCODE	intergenic	
DQ571357	uc010lcl.3	0.025	7.299	0.008	6.343	UCSC_knowngene	intergenic	
CCDC144B	ENST00000445752	0.014	13.465	0.008	6.326	GENCODE	intergenic	
AP006222.2	ENST00000442116	0.009	5.537	0.009	6.319	GENCODE	intergenic	
XLOC_011753	TCONS_00024861	0.021	11.208	0.014	6.291	LincRNAs identified by Cabili et al	intergenic	
TPT1-AS1	ENST00000522673	0.031	7.674	0.011	6.235	GENCODE	intergenic	
CTD-2329K10.1	ENST00000561324	0.041	5.093	0.014	6.221	GENCODE	intronic antisense	CTDSPL2
AC005682.5	ENST00000415611	0.043	9.356	0.035	6.189	GENCODE	intergenic	
RP11-94C24.3	ENST00000507161	0.016	10.685	0.009	6.188	GENCODE	bidirectional	RSAD1

AL592284.1	ENST00000426786	0.021	9.186	0.011	6.181	GENCODE	natural antisense	AL592284.2
RP4-794H19.4	ENST00000438195	0.013	13.509	0.011	6.180	GENCODE	intergenic	
GNPDA2	NR_073094	0.019	10.511	0.008	6.166	RefSeq	exon sense-overlapping	GNPDA2
LOC339535	NR_015407	0.029	39.175	0.010	6.165	RefSeq	intergenic	
AK000807	uc001rie.1	0.025	12.753	0.015	6.163	UCSC_knowngene	exon sense-overlapping	PPFIBP1
RP11-983P16.4	ENST00000552905	0.050	5.527	0.016	6.158	GENCODE	intronic antisense	EIF4B
TPT1-AS1	ENST00000520585	0.018	7.395	0.005	6.152	GENCODE	intergenic	
AK126380	uc001luy.1	0.029	5.234	0.012	6.124	UCSC_knowngene	intron sense-overlapping	MRPL23
PRR24	NR_037675	0.023	6.647	0.013	6.102	RefSeq	exon sense-overlapping	PRR24
LOC386758	NR_037161	0.007	30.215	0.013	6.091	RefSeq	bidirectional	ZNF582
BMS1P1	ENST00000374336	0.042	5.203	0.007	6.084	pseudogene		
XLOC_005939	TCONS_00012373	0.014	6.971	0.009	6.056	LincRNAs identified by Cabili et al	intergenic	
BC069782	uc001kfm.3	0.049	5.342	0.015	6.025	UCSC_knowngene	exon sense-overlapping	STAMBPL1
RP4-650F12.2	ENST00000411417	0.007	11.972	0.002	6.009	GENCODE	intron sense-overlapping	FPGT-TNNI3K
XLOC_004231	TCONS_00008717	0.025	48.982	0.002	6.003	LincRNAs identified by Cabili et al	intergenic	
	R66218	0.032	9.947	0.016	6.003	LincRNAs identified by Khalil et al	intergenic	
	BX093283	0.029	12.764	0.014	5.956	LincRNAs identified by Khalil et al	intergenic	
XLOC_012259	TCONS_00025441	0.012	7.145	0.015	5.931	LincRNAs identified by Cabili et al	intergenic	
XLOC_002561	TCONS_00004653	0.044	5.119	0.006	5.875	LincRNAs identified by Cabili et al	intergenic	
RP11-111F5.5	ENST00000377614	0.015	7.679	0.004	5.843	GENCODE	intergenic	
XLOC_006520	TCONS_00014352	0.021	9.673	0.013	5.825	LincRNAs identified by Cabili et al	intergenic	
AC093642.3	ENST00000451070	0.016	6.309	0.011	5.822	GENCODE	intergenic	
RP11-486A14.2	ENST00000552835	0.008	5.931	0.007	5.776	GENCODE	bidirectional	NUDT4
RP11-693N9.2	ENST00000534659	0.031	37.892	0.046	5.770	GENCODE	intergenic	
RP11-345M22.2	ENST00000566729	0.025	11.681	0.017	5.769	GENCODE	intergenic	
AC072062.1	ENST00000420134	0.007	9.840	0.002	5.740	GENCODE	intronic antisense	ABCA12
RP3-332B22.1	ENST00000418834	0.017	16.341	0.007	5.725	GENCODE	intergenic	
AF070581	uc004epb.3	0.016	7.038	0.004	5.713	UCSC_knowngene	intron sense-overlapping	PAK3
LOC646813	uc001nhf.1	0.044	6.771	0.032	5.708	UCSC_knowngene	intergenic	
RP13-977J11.2	ENST00000538731	0.013	6.973	0.011	5.683	GENCODE	intergenic	
RP11-345M22.2	ENST00000562921	0.046	5.719	0.004	5.680	GENCODE	intergenic	
RP11-493L12.4	ENST00000552063	0.025	12.619	0.013	5.592	GENCODE	intergenic	
RP11-125B21.2	ENST00000447278	0.021	9.739	0.007	5.567	GENCODE	intergenic	
RP11-33B1.1	ENST00000513147	0.016	5.663	0.009	5.566	GENCODE	intergenic	
AC092620.2	ENST00000449869	0.021	8.670	0.013	5.544	GENCODE	exon sense-overlapping	SPOPL
BX648501	uc003zmh.1	0.024	6.623	0.006	5.534	UCSC_knowngene	intronic antisense	C9orf92
ACAD9	NR_033426	0.021	5.142	0.011	5.513	RefSeq	exon sense-overlapping	ACAD9
PPWD1	NR_046350	0.025	6.152	0.014	5.490	RefSeq	exon sense-overlapping	PPWD1

RP11-983P16.4	ENST00000549388	0.019	10.170	0.028	5.484	GENCODE	natural antisense	EIF4B
TMEM161B-AS1	NR_039993	0.021	10.259	0.007	5.455	RefSeq	bidirectional	TMEM161B
SNHG7	NR_024543	0.020	9.125	0.004	5.431	RefSeq	natural antisense	FAM69B
RP11-693N9.2	ENST00000527637	0.010	12.781	0.019	5.417	GENCODE	intergenic	
RP11-791G16.2	ENST00000488045	0.035	10.162	0.032	5.374	GENCODE	exon sense-overlapping	TMEM150C
PPP4R1	NR_052003	0.019	5.223	0.006	5.372	RefSeq	exon sense-overlapping	PPP4R1
AC118138.2	ENST00000420642	0.022	5.363	0.026	5.369	GENCODE	intergenic	
BC063788	uc022agm.1	0.012	14.210	0.006	5.365	UCSC_knowngene	exon sense-overlapping	SRRM3
CR936688	uc003gpk.1	0.032	5.619	0.007	5.345	UCSC_knowngene	natural antisense	FAM184B
	AK025194	0.014	12.921	0.027	5.345	NRED	intergenic	
RP11-105C19.2	ENST00000567401	0.023	9.818	0.020	5.340	GENCODE	intergenic	
AC011747.7	ENST00000412712	0.023	7.968	0.015	5.299	GENCODE	bidirectional	ID2
RP11-14N7.2	ENST00000420597	0.017	5.596	0.004	5.276	GENCODE	intergenic	
RP11-161M6.5	ENST00000564390	0.022	15.130	0.004	5.274	GENCODE	intergenic	
XLOC_006409	TCONS_00014282	0.021	15.240	0.003	5.255	LincRNAs identified by Cabili et al	intergenic	
SNHG8	NR_034010	0.012	7.080	0.006	5.226	RefSeq	intergenic	
EEF1E1-MUTED	NR_037618	0.021	6.256	0.010	5.210	RefSeq	exon sense-overlapping	EEF1E1
RP11-299L17.3	ENST00000555001	0.014	12.848	0.011	5.192	GENCODE	intergenic	
CR627394	uc004edk.1	0.025	6.438	0.023	5.179	UCSC_knowngene	intergenic	
AC034220.3	ENST00000457998	0.027	5.319	0.010	5.179	GENCODE	bidirectional	SLC22A5
RP11-167J8.2	ENST00000527566	0.006	6.094	0.005	5.175	GENCODE	intergenic	
RP3-327A19.5	ENST00000455986	0.029	7.936	0.006	5.168	GENCODE	intergenic	
LOC100133331	NR_028327	0.036	5.283	0.003	5.154	RefSeq	intergenic	
LOC100131067	NR_034121	0.025	5.189	0.015	5.129	RefSeq	intronic antisense	CKMT2
SNHG8	NR_003584	0.023	10.757	0.011	5.127	RefSeq	intergenic	
RP11-227D13.1	ENST00000558061	0.030	6.931	0.023	5.102	GENCODE	bidirectional	FBN1
LOC283174	NR_024344	0.044	9.956	0.045	5.060	RefSeq	intergenic	
ZNF826P	uc010ecl.2	0.020	13.242	0.002	5.047	UCSC_knowngene	intergenic	
AY343892	uc003zfx.3	0.010	8.387	0.002	5.030	UCSC_knowngene	intergenic	
RP4-717I23.3	ENST00000457387	0.006	5.975	0.018	5.024	GENCODE	bidirectional	DR1

Table S4. The qPCR primers used in this study

Gene	Forword(5'-3')	Reverse(5'-3')
AC092214.10	GGTTGTGCATTATATCCAAGTGTGTA	GAATGGTTTTATTTGTACTTTGGTATGAGA
CYP3A5	AACTGAGTCCCACAAAGCTCTGT	GGTGGTTTCATAGCCAGCAAA
LEMD1	TGGAAAATAAGTCGCTGTTTGGT	GGTAGTGTTTTGGTTCTTTCCTGAA
PART1	CACCTCCTACAACTCCGAAGAAA	ACTTGAATGACTGCGGATGGT
RNF157-AS1	GAGCCGTCAGGTCCACATG	GTCATCCTATTGCTTCTGCTTTAGC
RP11-532F12.5	AAGGGCCTGCTGTCTTCCA	GCTAAAGATGCAAGATGGCTTCA
AC010680.1	GATGAAGGAAAAGCCCAAGAAA	AGAACCCTGTTGGCAAAGACA
ADAMTS9-AS1	TCCACAGCATTACAGCAGCAA	TTTGGCTCCAATTCTCAGGATT
ADAMTS9-AS2	CTCTGAAACTCCTGGCCTCAA	GCAATGGCTCATACCTATAGTTCCA
AK021537	AGATGCTGATGAGCCCCCTAT	GGTGCCAGCAGCTGCATT
AK125532	CCTGGAATTCAAGCTTCTACAAGGT	GGCAAGGCTTGAATGATGCT
LOC386758	AAGGACGTGGACACACAAGAAA	CACGTGTACAGCAAGGAACATACA
RP1-78O14.1	GGCTTTTGATCCAGCTGTTCTT	CCCCACTCCATCCTTCTCTCT
RP11-597D13.9	TCTCCAATCTTAACCAGGTTTGC	CCCTTCAGCCGCAGGAA
LEMD1-AS1	GCTCCAGCGCCCACTTT	CCATGTCTCTTCTCCAGCTCTACA
FAM198B	AGGGCAATGGGTCCACTCT	CTTCACGGTGCCACGGATA
ADAMTS9	ATTAGAGACCCTGAGCGAATACG	GAAGTGGACGTTCGTGGGAA
GAPDH	CCAGAACATCATCCCTGCCT	CCTGCTTCACCACCTTCTTG

Table S5A. The relation of mRNA and lncRNA of profile NO. 15

Gene1/lncRNA1	type 1	Gene2/lncRNA2	type 2	interaction
HNRNPH3	protein_coding	OBSL1	protein_coding	0.993
HNRNPH3	protein_coding	FHL5	protein_coding	0.997
HNRNPH3	protein_coding	ENST00000512246	noncoding	0.995
CNBP	protein_coding	ZNF323	protein_coding	0.994
CNBP	protein_coding	VWC2	protein_coding	0.993
CNBP	protein_coding	uc003lta.2	noncoding	0.993
CNBP	protein_coding	uc001vgk.2	noncoding	0.994
CNBP	protein_coding	TCONS_00022431	noncoding	0.991
CNBP	protein_coding	SERGEF	protein_coding	0.991
CNBP	protein_coding	POLR3GL	protein_coding	0.992
CNBP	protein_coding	NR_033919	noncoding	0.991
CNBP	protein_coding	MGARP	protein_coding	0.991
CNBP	protein_coding	GLT8D2	protein_coding	0.994
CNBP	protein_coding	ENST00000584201	noncoding	0.991
CNBP	protein_coding	ENST00000444672	noncoding	0.991
CNBP	protein_coding	ENST00000442116	noncoding	0.995
CNBP	protein_coding	EIF4A2	protein_coding	0.992
CNBP	protein_coding	CNRIP1	protein_coding	0.991
YTHDC1	protein_coding	VWC2	protein_coding	0.997
YTHDC1	protein_coding	EIF4A2	protein_coding	0.995
YTHDC1	protein_coding	EIF2D	protein_coding	0.992
PLEKHA5	protein_coding	uc001zfw.1	noncoding	0.992
PLEKHA5	protein_coding	OBSL1	protein_coding	0.990
PLEKHA5	protein_coding	KANSL1L	protein_coding	0.994
PLEKHA5	protein_coding	FHL5	protein_coding	0.992
PLEKHA5	protein_coding	ENST00000571724	noncoding	0.991
PLEKHA5	protein_coding	ENST00000512246	noncoding	0.992
PLEKHA5	protein_coding	ENST00000507571	noncoding	0.996
PLEKHA5	protein_coding	ENST00000435697	noncoding	0.993
PLEKHA5	protein_coding	CD242780	noncoding	0.990
ZC4H2	protein_coding	SULF2	protein_coding	0.995
ZC4H2	protein_coding	PPP3CB	protein_coding	0.997
ZC4H2	protein_coding	ZBTB20	protein_coding	0.994
ZC4H2	protein_coding	uc001zfw.1	noncoding	0.991
ZC4H2	protein_coding	RDX	protein_coding	0.993
ZC4H2	protein_coding	NR_027133	noncoding	0.990
ZC4H2	protein_coding	FEZ2	protein_coding	0.994
ZC4H2	protein_coding	FAM18B1	protein_coding	0.990
ZC4H2	protein_coding	FABP4	protein_coding	0.995
ZC4H2	protein_coding	ENST00000507571	noncoding	0.992
ZC4H2	protein_coding	ENST00000494740	noncoding	0.999
ZC4H2	protein_coding	ENST00000444375	noncoding	0.991

ZC4H2	protein_coding	CPQ	protein_coding	0.991
ZC4H2	protein_coding	CIRBP	protein_coding	0.991
TTC8	protein_coding	ENST00000564660	noncoding	0.998
TTC8	protein_coding	ENST00000453754	noncoding	0.991
CRBN	protein_coding	CD242780	noncoding	0.994
TRABD2A	protein_coding	NSA2	protein_coding	0.991
TRABD2A	protein_coding	NR_029389	noncoding	0.991
TRABD2A	protein_coding	ENST00000508742	noncoding	0.995
TRABD2A	protein_coding	CCT6B	protein_coding	0.997
VIT	protein_coding	uc003kkf.2	noncoding	0.992
VIT	protein_coding	uc003kke.2	noncoding	0.991
VIT	protein_coding	TCONS_00025350	noncoding	0.994
VIT	protein_coding	SPRR2F	protein_coding	0.993
VIT	protein_coding	SPRR2A	protein_coding	0.990
VIT	protein_coding	NGF	protein_coding	0.992
VIT	protein_coding	GPRASP1	protein_coding	0.991
VIT	protein_coding	ENST00000571724	noncoding	0.994
VIT	protein_coding	ENST00000562921	noncoding	0.990
VIT	protein_coding	ENST00000512585	noncoding	0.993
VIT	protein_coding	ENST00000493124	noncoding	0.997
VIT	protein_coding	ENST00000471990	noncoding	0.997
VIT	protein_coding	ENST00000435697	noncoding	0.991
VIT	protein_coding	ENST00000433460	noncoding	0.994
VIT	protein_coding	ENST00000429109	noncoding	0.991
VIT	protein_coding	ENST00000419627	noncoding	0.995
VIT	protein_coding	CNRIP1	protein_coding	0.993
VIT	protein_coding	CDH8	protein_coding	0.991
VIT	protein_coding	BEND5	protein_coding	0.994
VIT	protein_coding	B3GNT1	protein_coding	0.994
SLC25A27	protein_coding	PON3	protein_coding	0.990
SLC25A27	protein_coding	NR1H4	protein_coding	0.992
SLC25A27	protein_coding	NR_034004	noncoding	0.995
SLC25A27	protein_coding	NKAPL	protein_coding	0.993
SLC25A27	protein_coding	ENST00000495228	noncoding	0.992
SLC25A27	protein_coding	ENST00000422799	noncoding	0.992
SLC25A27	protein_coding	ENST00000416111	noncoding	0.992
SLC25A27	protein_coding	ENST00000412376	noncoding	0.991
GLT8D2	protein_coding	ENST00000562921	noncoding	0.993
GLT8D2	protein_coding	ENST00000451070	noncoding	0.993
GLT8D2	protein_coding	ENST00000444672	noncoding	0.997
GLT8D2	protein_coding	ENST00000442116	noncoding	0.996
GLT8D2	protein_coding	DOK6	protein_coding	0.993
GLT8D2	protein_coding	CDH8	protein_coding	0.992
CD46	protein_coding	ZNF599	protein_coding	0.992

CD46	protein_coding	uc003kke.2	noncoding	0.992
CD46	protein_coding	TCONS_00027009	noncoding	0.994
CD46	protein_coding	TCONS_00025350	noncoding	0.994
CD46	protein_coding	TCONS_00023799	noncoding	0.992
CD46	protein_coding	SPRR2F	protein_coding	0.991
CD46	protein_coding	SLC16A14	protein_coding	0.991
CD46	protein_coding	SERGEF	protein_coding	0.992
CD46	protein_coding	POLR3GL	protein_coding	0.991
CD46	protein_coding	PCGF6	protein_coding	0.991
CD46	protein_coding	MXRA7	protein_coding	0.994
CD46	protein_coding	GLT8D2	protein_coding	0.992
CD46	protein_coding	FAM156B	protein_coding	0.991
CD46	protein_coding	ENST00000562921	noncoding	0.993
CD46	protein_coding	ENST00000554749	noncoding	0.994
CD46	protein_coding	ENST00000512585	noncoding	0.991
CD46	protein_coding	ENST00000466225	noncoding	0.991
CD46	protein_coding	ENST00000444672	noncoding	0.990
CD46	protein_coding	ENST00000429109	noncoding	0.996
CD46	protein_coding	DOK6	protein_coding	0.994
CD46	protein_coding	CDH8	protein_coding	0.998
ZNF599	protein_coding	ZNF323	protein_coding	0.992
ZNF599	protein_coding	SERGEF	protein_coding	0.996
ZNF599	protein_coding	HFE	protein_coding	0.996
ZNF599	protein_coding	FAM156B	protein_coding	0.992
ZNF599	protein_coding	ENST00000466225	noncoding	0.994
ZNF599	protein_coding	CDH8	protein_coding	0.995
PCGF6	protein_coding	ENST00000554749	noncoding	0.994
PCGF6	protein_coding	ENST00000466225	noncoding	0.994
MAOA	protein_coding	ENST00000507571	noncoding	0.990
MAOA	protein_coding	ENST00000435697	noncoding	0.991
KANSL1L	protein_coding	ENST00000495228	noncoding	0.991
KANSL1L	protein_coding	DNAJC18	protein_coding	0.991
RDX	protein_coding	POLR3GL	protein_coding	0.990
RDX	protein_coding	NR_033919	noncoding	0.993
RDX	protein_coding	MGARP	protein_coding	0.994
RDX	protein_coding	ENST00000507571	noncoding	0.992
RDX	protein_coding	ENST00000494740	noncoding	0.992
RDX	protein_coding	ENST00000444672	noncoding	0.994
RDX	protein_coding	ENST00000442116	noncoding	0.996
RDX	protein_coding	DNAJB4	protein_coding	0.991
RDX	protein_coding	CIRBP	protein_coding	0.992
RDX	protein_coding	BEND5	protein_coding	0.992
RDX	protein_coding	B3GNT1	protein_coding	0.992
MGARP	protein_coding	HFE	protein_coding	0.991

MGARP	protein_coding	ENST00000444672	noncoding	0.993
MGARP	protein_coding	ENST00000442116	noncoding	0.996
MGARP	protein_coding	DNAJB4	protein_coding	0.992
MGARP	protein_coding	CDH8	protein_coding	0.990
MGARP	protein_coding	AMT	protein_coding	0.997
TDGF1	protein_coding	STAMBPL1	protein_coding	0.995
TDGF1	protein_coding	SETDB2	protein_coding	0.993
TDGF1	protein_coding	NTF3	protein_coding	0.992
TDGF1	protein_coding	NR_033919	noncoding	0.992
TDGF1	protein_coding	MAB21L1	protein_coding	0.994
TDGF1	protein_coding	ENST00000416111	noncoding	0.995
TDGF1	protein_coding	EEF1A1	protein_coding	0.991
TDGF1	protein_coding	CIRBP	protein_coding	0.996
SF1	protein_coding	NR_029389	noncoding	0.993
SF1	protein_coding	LIN52	protein_coding	0.991
SF1	protein_coding	FAM18B1	protein_coding	0.993
SF1	protein_coding	ENST00000451070	noncoding	0.991
SF1	protein_coding	ENST00000444672	noncoding	0.990
MAB21L1	protein_coding	KIF16B	protein_coding	0.993
MAB21L1	protein_coding	GPRASP1	protein_coding	0.992
MAB21L1	protein_coding	ENST00000493124	noncoding	0.993
MAB21L1	protein_coding	ENST00000471990	noncoding	0.993
MAB21L1	protein_coding	ENST00000453754	noncoding	0.995
MAB21L1	protein_coding	ENST00000416111	noncoding	0.996
MAB21L1	protein_coding	EEF1A1	protein_coding	0.990
MAB21L1	protein_coding	DNAJC18	protein_coding	0.990
MAB21L1	protein_coding	CNRIP1	protein_coding	0.995
MAB21L1	protein_coding	CIRBP	protein_coding	0.992
NSA2	protein_coding	GRIK2	protein_coding	0.994
NSA2	protein_coding	ENST00000569927	noncoding	0.998
NSA2	protein_coding	ENST00000512585	noncoding	0.991
NSA2	protein_coding	ENST00000506463	noncoding	0.993
NSA2	protein_coding	ENST00000451070	noncoding	0.990
NSA2	protein_coding	ENST00000413525	noncoding	0.993
NSA2	protein_coding	DYNC1I2	protein_coding	0.997
NSA2	protein_coding	ARSE	protein_coding	0.991
HNRNPA1L2	protein_coding	GPRASP1	protein_coding	0.994
HNRNPA1L2	protein_coding	ENST00000584201	noncoding	0.993
HNRNPA1L2	protein_coding	ENST00000571724	noncoding	0.993
HNRNPA1L2	protein_coding	ENST00000508718	noncoding	0.993
HNRNPA1L2	protein_coding	ENST00000435697	noncoding	0.992
SIRT4	protein_coding	ENST00000547777	noncoding	0.991
SIRT4	protein_coding	ENST00000512585	noncoding	0.994
SIRT4	protein_coding	ENST00000509363	noncoding	0.992

SIRT4	protein_coding	ENST00000429109	noncoding	0.992
ZNF181	protein_coding	TCONS_00001581	noncoding	0.990
ZNF181	protein_coding	SLC25A27	protein_coding	0.992
ZNF181	protein_coding	SLC16A14	protein_coding	0.990
ZNF181	protein_coding	OLFM1	protein_coding	0.990
ZNF181	protein_coding	NR_034004	noncoding	0.996
ZNF181	protein_coding	NR_026836	noncoding	0.992
ZNF181	protein_coding	ENST00000422799	noncoding	0.997
ZNF181	protein_coding	EID3	protein_coding	0.993
ZNF181	protein_coding	AF070541	noncoding	0.991
PRSS35	protein_coding	NSA2	protein_coding	0.993
PRSS35	protein_coding	NR_073094	noncoding	0.990
PRSS35	protein_coding	ENST00000569927	noncoding	0.994
PRSS35	protein_coding	ENST00000562501	noncoding	0.992
ZNF518B	protein_coding	TCONS_00001581	noncoding	0.993
ZNF518B	protein_coding	STAMBPL1	protein_coding	0.990
ZNF518B	protein_coding	SLC25A27	protein_coding	0.991
ZNF518B	protein_coding	PON3	protein_coding	0.993
ZBTB20	protein_coding	uc010ocf.2	noncoding	0.994
ZBTB20	protein_coding	HFM1	protein_coding	0.995
ZBTB20	protein_coding	FEZ2	protein_coding	0.997
ZBTB20	protein_coding	FABP4	protein_coding	0.990
ZBTB20	protein_coding	ENST00000494740	noncoding	0.996
ZBTB20	protein_coding	CPQ	protein_coding	0.996
ZBTB20	protein_coding	CD242780	noncoding	0.993
SPRR2F	protein_coding	SLC35A5	protein_coding	0.993
SPRR2F	protein_coding	SERHL2	protein_coding	0.992
SPRR2F	protein_coding	RDX	protein_coding	0.991
SPRR2F	protein_coding	POLR3GL	protein_coding	0.991
SPRR2F	protein_coding	NSA2	protein_coding	0.992
SPRR2F	protein_coding	NR_033919	noncoding	0.997
SPRR2F	protein_coding	NR_029389	noncoding	0.994
SPRR2F	protein_coding	GLT8D2	protein_coding	0.994
SPRR2F	protein_coding	ENST00000562921	noncoding	0.994
SPRR2F	protein_coding	ENST00000512585	noncoding	0.995
SPRR2F	protein_coding	ENST00000493124	noncoding	0.996
SPRR2F	protein_coding	ENST00000471990	noncoding	0.995
SPRR2F	protein_coding	ENST00000451070	noncoding	0.996
SPRR2F	protein_coding	ENST00000444672	noncoding	0.998
SPRR2F	protein_coding	ENST00000442116	noncoding	0.993
SPRR2F	protein_coding	ENST00000429109	noncoding	0.991
SPRR2F	protein_coding	DYNC1I2	protein_coding	0.991
SPRR2F	protein_coding	DOK6	protein_coding	0.993
SPRR2F	protein_coding	DNAJB4	protein_coding	0.992

SPRR2F	protein_coding	CNRIP1	protein_coding	0.992
SPRR2F	protein_coding	CDH8	protein_coding	0.993
SPRR2F	protein_coding	BEND5	protein_coding	0.994
SPRR2F	protein_coding	B3GNT1	protein_coding	0.993
SPRR2F	protein_coding	ARSE	protein_coding	0.990
MXRA7	protein_coding	ENST00000554749	noncoding	0.997
MXRA7	protein_coding	ENST00000512585	noncoding	0.990
MXRA7	protein_coding	ENST00000509363	noncoding	0.991
MXRA7	protein_coding	ENST00000429109	noncoding	0.997
MXRA7	protein_coding	EID3	protein_coding	0.995
MXRA7	protein_coding	DOK6	protein_coding	0.994
CNRIP1	protein_coding	CIRBP	protein_coding	0.992
CNRIP1	protein_coding	CD242780	noncoding	0.992
CNRIP1	protein_coding	BEND5	protein_coding	0.993
CNRIP1	protein_coding	B3GNT1	protein_coding	0.991
SPRR2A	protein_coding	SLC25A27	protein_coding	0.992
SPRR2A	protein_coding	NR1H4	protein_coding	0.992
SPRR2A	protein_coding	NR_034004	noncoding	0.992
SPRR2A	protein_coding	NGF	protein_coding	0.992
SPRR2A	protein_coding	GPRASP1	protein_coding	0.995
SPRR2A	protein_coding	ENST00000515376	noncoding	0.993
SPRR2A	protein_coding	ENST00000509363	noncoding	0.992
SPRR2A	protein_coding	ENST00000493124	noncoding	0.993
SPRR2A	protein_coding	ENST00000471990	noncoding	0.994
SPRR2A	protein_coding	ENST00000429109	noncoding	0.991
SPRR2A	protein_coding	ENST00000413645	noncoding	0.993
SPRR2A	protein_coding	ENST00000412376	noncoding	0.990
SPRR2A	protein_coding	C6orf225	protein_coding	0.991
SPRR2A	protein_coding	AKAP12	protein_coding	0.992
MMP21	protein_coding	ENST00000507571	noncoding	0.990
MMP21	protein_coding	DNAJB4	protein_coding	0.996
VEZT	protein_coding	uc003lta.2	noncoding	0.995
VEZT	protein_coding	TCONS_00022431	noncoding	0.991
VEZT	protein_coding	TCONS_00001581	noncoding	0.990
VEZT	protein_coding	NR_029389	noncoding	0.995
VEZT	protein_coding	GLT8D2	protein_coding	0.993
VEZT	protein_coding	FNDC4	protein_coding	0.993
VEZT	protein_coding	ENST00000415026	noncoding	0.992
VWC2	protein_coding	uc001vgk.2	noncoding	0.992
VWC2	protein_coding	TCONS_00022431	noncoding	0.990
VWC2	protein_coding	MAB21L1	protein_coding	0.990
VWC2	protein_coding	ENST00000584201	noncoding	0.992
VWC2	protein_coding	ENST00000508718	noncoding	0.991
VWC2	protein_coding	EIF4A2	protein_coding	0.998

VWC2	protein_coding	EIF2D	protein_coding	0.993
SETDB2	protein_coding	NR_027132	noncoding	0.990
SETDB2	protein_coding	ENST00000507571	noncoding	0.993
SETDB2	protein_coding	CIRBP	protein_coding	0.998
SETDB2	protein_coding	CD242780	noncoding	0.990
SETDB2	protein_coding	BEND5	protein_coding	0.991
SETDB2	protein_coding	B3GNT1	protein_coding	0.991
SERHL2	protein_coding	PRSS35	protein_coding	0.995
SERHL2	protein_coding	NSA2	protein_coding	0.996
SERHL2	protein_coding	NR_073094	noncoding	0.993
SERHL2	protein_coding	NR_029389	noncoding	0.990
SERHL2	protein_coding	MYL6B	protein_coding	0.990
SERHL2	protein_coding	GRIK2	protein_coding	0.991
SERHL2	protein_coding	ENST00000569927	noncoding	0.998
SERHL2	protein_coding	ENST00000562501	noncoding	0.994
SERHL2	protein_coding	ENST00000512585	noncoding	0.994
SERHL2	protein_coding	ENST00000506463	noncoding	0.995
SERHL2	protein_coding	ENST00000451070	noncoding	0.993
SERHL2	protein_coding	ENST00000413525	noncoding	0.994
SERHL2	protein_coding	DYNC1I2	protein_coding	0.993
SERHL2	protein_coding	DNAJC12	protein_coding	0.990
SERHL2	protein_coding	ARSE	protein_coding	0.991
FNDC4	protein_coding	EEF1A1	protein_coding	0.992
NGF	protein_coding	HNRNPA1L2	protein_coding	0.995
NGF	protein_coding	GPRASP1	protein_coding	0.994
NGF	protein_coding	ENST00000584201	noncoding	0.993
NGF	protein_coding	ENST00000571724	noncoding	0.994
NGF	protein_coding	ENST00000515376	noncoding	0.993
NGF	protein_coding	ENST00000493124	noncoding	0.991
NGF	protein_coding	ENST00000471990	noncoding	0.993
NGF	protein_coding	EIF2D	protein_coding	0.991
NGF	protein_coding	CNRIP1	protein_coding	0.990
BEND5	protein_coding	B3GNT1	protein_coding	0.999
GPRASP1	protein_coding	ENST00000571724	noncoding	0.995
GPRASP1	protein_coding	ENST00000567753	noncoding	0.994
GPRASP1	protein_coding	ENST00000508718	noncoding	0.992
GPRASP1	protein_coding	ENST00000495228	noncoding	0.991
GPRASP1	protein_coding	ENST00000493124	noncoding	0.993
GPRASP1	protein_coding	ENST00000471990	noncoding	0.994
GPRASP1	protein_coding	ENST00000435697	noncoding	0.994
GPRASP1	protein_coding	ENST00000419627	noncoding	0.992
GPRASP1	protein_coding	ENST00000413645	noncoding	0.998
GPRASP1	protein_coding	CNRIP1	protein_coding	0.995
GPRASP1	protein_coding	AKAP12	protein_coding	0.992

SULF2	protein_coding	ZBTB20	protein_coding	0.991
SULF2	protein_coding	WNT5B	protein_coding	0.994
SULF2	protein_coding	uc010ocf.2	noncoding	0.993
SULF2	protein_coding	LIN52	protein_coding	0.994
SULF2	protein_coding	FEZ2	protein_coding	0.993
SULF2	protein_coding	FAM18B1	protein_coding	0.995
SULF2	protein_coding	FABP4	protein_coding	0.991
SULF2	protein_coding	ENST00000508742	noncoding	0.991
SULF2	protein_coding	ENST00000494740	noncoding	0.996
SULF2	protein_coding	ENST00000444375	noncoding	0.992
SPRR2E	protein_coding	SPRR2A	protein_coding	0.995
SPRR2E	protein_coding	NR1H4	protein_coding	0.991
SPRR2E	protein_coding	NGF	protein_coding	0.997
SPRR2E	protein_coding	KIF16B	protein_coding	0.992
SPRR2E	protein_coding	HNRNPA1L2	protein_coding	0.992
SPRR2E	protein_coding	GPRASP1	protein_coding	0.994
SPRR2E	protein_coding	ENST00000515376	noncoding	0.997
SPRR2E	protein_coding	ENST00000509363	noncoding	0.991
SPRR2E	protein_coding	ENST00000471990	noncoding	0.990
SPRR2E	protein_coding	ENST00000413645	noncoding	0.992
SPRR2E	protein_coding	EIF2D	protein_coding	0.993
EIF4A2	protein_coding	EIF2D	protein_coding	0.993
DFNA5	protein_coding	TBC1D8B	protein_coding	0.991
DFNA5	protein_coding	SCARB1	protein_coding	0.991
DFNA5	protein_coding	AMT	protein_coding	0.992
OBSL1	protein_coding	FHL5	protein_coding	0.996
OBSL1	protein_coding	ENST00000584201	noncoding	0.991
OBSL1	protein_coding	ENST00000512246	noncoding	0.999
OBSL1	protein_coding	ENST00000508718	noncoding	0.991
POLR3GL	protein_coding	NUDT11	protein_coding	0.990
POLR3GL	protein_coding	NTF3	protein_coding	0.991
POLR3GL	protein_coding	NR_033919	noncoding	0.996
POLR3GL	protein_coding	MGARP	protein_coding	0.990
POLR3GL	protein_coding	MAB21L1	protein_coding	0.990
POLR3GL	protein_coding	GLT8D2	protein_coding	0.994
POLR3GL	protein_coding	ENST00000562921	noncoding	0.993
POLR3GL	protein_coding	ENST00000493124	noncoding	0.990
POLR3GL	protein_coding	ENST00000444672	noncoding	0.995
POLR3GL	protein_coding	ENST00000442116	noncoding	0.996
POLR3GL	protein_coding	DOK6	protein_coding	0.993
POLR3GL	protein_coding	CDH8	protein_coding	0.993
POLR3GL	protein_coding	BEND5	protein_coding	0.991
NTF3	protein_coding	NR_033919	noncoding	0.993
NTF3	protein_coding	GLT8D2	protein_coding	0.992

NTF3	protein_coding	EEF1A1	protein_coding	0.998
CIRBP	protein_coding	CD242780	noncoding	0.992
CIRBP	protein_coding	BEND5	protein_coding	0.994
CIRBP	protein_coding	B3GNT1	protein_coding	0.992
TDRD10	protein_coding	CNBP	protein_coding	0.991
TDRD10	protein_coding	CD46	protein_coding	0.996
TDRD10	protein_coding	VEZT	protein_coding	0.994
TDRD10	protein_coding	uc003lta.2	noncoding	0.991
TDRD10	protein_coding	uc003kke.2	noncoding	0.993
TDRD10	protein_coding	TCONS_00027009	noncoding	0.994
TDRD10	protein_coding	TCONS_00025350	noncoding	0.996
TDRD10	protein_coding	TCONS_00023799	noncoding	0.990
TDRD10	protein_coding	TCONS_00022431	noncoding	0.991
TDRD10	protein_coding	TCONS_00008647	noncoding	0.991
TDRD10	protein_coding	SPRR2F	protein_coding	0.995
TDRD10	protein_coding	NR_033919	noncoding	0.993
TDRD10	protein_coding	NR_029389	noncoding	0.997
TDRD10	protein_coding	GLT8D2	protein_coding	0.997
TDRD10	protein_coding	FAM156B	protein_coding	0.990
TDRD10	protein_coding	ENST00000562921	noncoding	0.993
TDRD10	protein_coding	ENST00000512585	noncoding	0.992
TDRD10	protein_coding	ENST00000493124	noncoding	0.992
TDRD10	protein_coding	ENST00000471990	noncoding	0.991
TDRD10	protein_coding	ENST00000451070	noncoding	0.991
TDRD10	protein_coding	ENST00000444672	noncoding	0.994
TDRD10	protein_coding	ENST00000442116	noncoding	0.991
TDRD10	protein_coding	ENST00000429109	noncoding	0.993
TDRD10	protein_coding	DOK6	protein_coding	0.993
TDRD10	protein_coding	CDH8	protein_coding	0.996
SPG7	protein_coding	OLFML3	protein_coding	0.996
SPG7	protein_coding	MGARP	protein_coding	0.990
SPG7	protein_coding	AMT	protein_coding	0.993
NUDT11	protein_coding	NR_034004	noncoding	0.993
NUDT11	protein_coding	BEND5	protein_coding	0.994
NUDT11	protein_coding	B3GNT1	protein_coding	0.992
NFIB	protein_coding	HFE	protein_coding	0.992
NFIB	protein_coding	ENST00000565166	noncoding	0.992
SERGEF	protein_coding	PHF10	protein_coding	0.990
SERGEF	protein_coding	NR_045029	noncoding	0.991
SERGEF	protein_coding	NR_040103	noncoding	0.993
SERGEF	protein_coding	MGARP	protein_coding	0.991
SERGEF	protein_coding	HFE	protein_coding	0.995
SERGEF	protein_coding	HARS2	protein_coding	0.991
SERGEF	protein_coding	FAM156B	protein_coding	0.995

SERGEF	protein_coding	CDH8	protein_coding	0.994
ZMAT1	protein_coding	NR_034004	noncoding	0.990
ZMAT1	protein_coding	NR_026836	noncoding	0.990
ZMAT1	protein_coding	ENST00000562501	noncoding	0.993
ZMAT1	protein_coding	DPP4	protein_coding	0.991
ZMAT1	protein_coding	DNAJC12	protein_coding	0.993
HFM1	protein_coding	ENST00000494740	noncoding	0.991
HFM1	protein_coding	ENST00000444375	noncoding	0.993
HFM1	protein_coding	CRBN	protein_coding	0.992
HFM1	protein_coding	CPQ	protein_coding	0.991
SLC16A14	protein_coding	SIRT4	protein_coding	0.992
SLC16A14	protein_coding	OLFM1	protein_coding	0.995
SLC16A14	protein_coding	NR_039993	noncoding	0.991
SLC16A14	protein_coding	NR_034004	noncoding	0.991
SLC16A14	protein_coding	NR_026836	noncoding	0.993
SLC16A14	protein_coding	MXRA7	protein_coding	0.995
SLC16A14	protein_coding	FNDC4	protein_coding	0.992
SLC16A14	protein_coding	ENST00000512585	noncoding	0.995
SLC16A14	protein_coding	ENST00000509363	noncoding	0.992
SLC16A14	protein_coding	ENST00000493124	noncoding	0.993
SLC16A14	protein_coding	ENST00000471990	noncoding	0.994
SLC16A14	protein_coding	ENST00000429109	noncoding	0.997
SLC16A14	protein_coding	EID3	protein_coding	0.997
SLC16A14	protein_coding	DOK6	protein_coding	0.995
SLC16A14	protein_coding	CDH8	protein_coding	0.991
PON3	protein_coding	NR1H4	protein_coding	0.990
PON3	protein_coding	ENST00000422799	noncoding	0.992
PON3	protein_coding	ENST00000412376	noncoding	0.991
FHL5	protein_coding	ENST00000584201	noncoding	0.990
FHL5	protein_coding	ENST00000512246	noncoding	0.998
FHL5	protein_coding	ENST00000507571	noncoding	0.992
METTL21C	protein_coding	ARSE	protein_coding	0.990
METTL21C	protein_coding	AMT	protein_coding	0.994
SLC35A5	protein_coding	SF1	protein_coding	0.991
SLC35A5	protein_coding	RDX	protein_coding	0.993
SLC35A5	protein_coding	NR_033919	noncoding	0.995
SLC35A5	protein_coding	ENST00000494740	noncoding	0.991
SLC35A5	protein_coding	ENST00000451070	noncoding	0.994
SLC35A5	protein_coding	ENST00000444672	noncoding	0.994
SLC35A5	protein_coding	CIRBP	protein_coding	0.992
SLC35A5	protein_coding	BEND5	protein_coding	0.991
CPQ	protein_coding	CD242780	noncoding	0.991
KIF16B	protein_coding	ENST00000453754	noncoding	0.992
KIF16B	protein_coding	AF070541	noncoding	0.992

MYL6B	protein_coding	ENST00000562501	noncoding	0.993
ZNF323	protein_coding	uc001vgk.2	noncoding	0.996
ZNF323	protein_coding	TCONS_00023799	noncoding	0.991
ZNF323	protein_coding	SERGEF	protein_coding	0.993
ZNF323	protein_coding	POLR3GL	protein_coding	0.992
ZNF323	protein_coding	NR_045634	noncoding	0.993
ZNF323	protein_coding	NR_045029	noncoding	0.992
ZNF323	protein_coding	MGARP	protein_coding	0.996
ZNF323	protein_coding	HFE	protein_coding	0.995
ZNF323	protein_coding	ENST00000584201	noncoding	0.990
ZNF323	protein_coding	ENST00000444672	noncoding	0.992
ZNF323	protein_coding	ENST00000442116	noncoding	0.993
ZNF323	protein_coding	DNAJB4	protein_coding	0.996
ZNF323	protein_coding	CDH8	protein_coding	0.992
OLFM1	protein_coding	NR_039993	noncoding	0.998
OLFM1	protein_coding	NR_026836	noncoding	0.997
OLFM1	protein_coding	MXRA7	protein_coding	0.998
OLFM1	protein_coding	ENST00000554749	noncoding	0.992
OLFM1	protein_coding	ENST00000509363	noncoding	0.992
OLFM1	protein_coding	ENST00000429109	noncoding	0.993
OLFM1	protein_coding	EID3	protein_coding	0.997
FEZ2	protein_coding	FABP4	protein_coding	0.992
FEZ2	protein_coding	ENST00000494740	noncoding	0.994
FEZ2	protein_coding	CPQ	protein_coding	0.994
WNT5B	protein_coding	LIN52	protein_coding	0.992
WNT5B	protein_coding	ENST00000508742	noncoding	0.990
LIN52	protein_coding	AMT	protein_coding	0.990
DNAJB4	protein_coding	CNRIP1	protein_coding	0.992
DNAJB4	protein_coding	CDH8	protein_coding	0.991
DNAJB4	protein_coding	BEND5	protein_coding	0.990
DOK6	protein_coding	CDH8	protein_coding	0.991
PPP3CB	protein_coding	uc001zfw.1	noncoding	0.990
PPP3CB	protein_coding	SPRR2F	protein_coding	0.992
PPP3CB	protein_coding	SLC35A5	protein_coding	0.991
PPP3CB	protein_coding	SETDB2	protein_coding	0.991
PPP3CB	protein_coding	RDX	protein_coding	0.998
PPP3CB	protein_coding	NR_033919	noncoding	0.993
PPP3CB	protein_coding	NR_027133	noncoding	0.992
PPP3CB	protein_coding	MMP21	protein_coding	0.992
PPP3CB	protein_coding	MGARP	protein_coding	0.991
PPP3CB	protein_coding	FABP4	protein_coding	0.990
PPP3CB	protein_coding	ENST00000507571	noncoding	0.996
PPP3CB	protein_coding	ENST00000494740	noncoding	0.996
PPP3CB	protein_coding	ENST00000444672	noncoding	0.993

PPP3CB	protein_coding	ENST00000442116	noncoding	0.995
PPP3CB	protein_coding	ENST00000435697	noncoding	0.992
PPP3CB	protein_coding	DNAJB4	protein_coding	0.991
PPP3CB	protein_coding	CNRIP1	protein_coding	0.994
PPP3CB	protein_coding	CIRBP	protein_coding	0.994
PPP3CB	protein_coding	BEND5	protein_coding	0.993
PPP3CB	protein_coding	B3GNT1	protein_coding	0.993
NR1H4	protein_coding	MAB21L1	protein_coding	0.992
NR1H4	protein_coding	KIF16B	protein_coding	0.996
NR1H4	protein_coding	GPRASP1	protein_coding	0.990
NR1H4	protein_coding	ENST00000564660	noncoding	0.991
NR1H4	protein_coding	ENST00000453754	noncoding	0.995
NR1H4	protein_coding	AKAP12	protein_coding	0.991
NR1H4	protein_coding	AF070541	noncoding	0.993
STAMBPL1	protein_coding	NTF3	protein_coding	0.991
STAMBPL1	protein_coding	MAB21L1	protein_coding	0.992
STAMBPL1	protein_coding	ENST00000453754	noncoding	0.991
STAMBPL1	protein_coding	ENST00000416111	noncoding	0.997
STAMBPL1	protein_coding	EEF1A1	protein_coding	0.994
ZNF385B	protein_coding	ZMAT1	protein_coding	0.992
ZNF385B	protein_coding	uc003kkf.2	noncoding	0.997
ZNF385B	protein_coding	uc003kke.2	noncoding	0.993
ZNF385B	protein_coding	TCONS_00025350	noncoding	0.994
ZNF385B	protein_coding	TCONS_00008647	noncoding	0.991
ZNF385B	protein_coding	TCONS_00001581	noncoding	0.993
ZNF385B	protein_coding	SPRR2F	protein_coding	0.992
ZNF385B	protein_coding	SLC16A14	protein_coding	0.990
ZNF385B	protein_coding	SERHL2	protein_coding	0.994
ZNF385B	protein_coding	NR_034004	noncoding	0.990
ZNF385B	protein_coding	NR_026836	noncoding	0.991
ZNF385B	protein_coding	MYL6B	protein_coding	0.994
ZNF385B	protein_coding	ENST00000562501	noncoding	0.996
ZNF385B	protein_coding	ENST00000512585	noncoding	0.997
ZNF385B	protein_coding	ENST00000493124	noncoding	0.993
ZNF385B	protein_coding	ENST00000471990	noncoding	0.992
ZNF385B	protein_coding	ENST00000429109	noncoding	0.994
ZNF385B	protein_coding	ENST00000413525	noncoding	0.991
ZNF385B	protein_coding	DOK6	protein_coding	0.991
ZNF385B	protein_coding	C6orf225	protein_coding	0.991
FAM156B	protein_coding	CDH8	protein_coding	0.992
TBC1D8B	protein_coding	SPG7	protein_coding	0.991
TBC1D8B	protein_coding	SCARB1	protein_coding	0.997
TBC1D8B	protein_coding	OLFML3	protein_coding	0.991
TBC1D8B	protein_coding	NR_045029	noncoding	0.997

TBC1D8B	protein_coding	NFIB	protein_coding	0.994
TBC1D8B	protein_coding	MGARP	protein_coding	0.992
TBC1D8B	protein_coding	AMT	protein_coding	0.994
GRIK2	protein_coding	ENST00000569927	noncoding	0.992
GRIK2	protein_coding	ENST00000562501	noncoding	0.992
GRIK2	protein_coding	ENST00000413525	noncoding	0.998
GRIK2	protein_coding	DYNC1I2	protein_coding	0.996
SCARB1	protein_coding	NR_045029	noncoding	0.997
SCARB1	protein_coding	NR_040103	noncoding	0.991
SCARB1	protein_coding	NR_024423	noncoding	0.992
SCARB1	protein_coding	NFIB	protein_coding	0.997
SCARB1	protein_coding	MGARP	protein_coding	0.991
SCARB1	protein_coding	HFE	protein_coding	0.990
SCARB1	protein_coding	ENST00000565166	noncoding	0.993
PHF10	protein_coding	FAM156B	protein_coding	0.993
PHF10	protein_coding	ENST00000565166	noncoding	0.991
FAM18B1	protein_coding	ENST00000494740	noncoding	0.992
FABP4	protein_coding	ENST00000494740	noncoding	0.995
FABP4	protein_coding	CPQ	protein_coding	0.991
ENST00000435697	noncoding	ENST00000419627	noncoding	0.993
ENST00000435697	noncoding	ENST00000413645	noncoding	0.991
ENST00000435697	noncoding	CNRIP1	protein_coding	0.997
NR_047522	noncoding	NR_027133	noncoding	0.991
NR_047522	noncoding	ENST00000526694	noncoding	0.991
NR_047522	noncoding	ENST00000415026	noncoding	0.996
NR_047522	noncoding	CNRIP1	protein_coding	0.990
NR_027133	noncoding	NR_027132	noncoding	0.998
NR_027133	noncoding	HFM1	protein_coding	0.991
NR_027133	noncoding	ENST00000562191	noncoding	0.999
NR_027133	noncoding	ENST00000494740	noncoding	0.991
NR_027133	noncoding	ENST00000444375	noncoding	0.993
NR_027133	noncoding	ENST00000415026	noncoding	0.991
NR_027133	noncoding	CRBN	protein_coding	0.993
NR_027133	noncoding	CNRIP1	protein_coding	0.990
NR_027133	noncoding	CIRBP	protein_coding	0.992
uc001vgk.2	noncoding	TCONS_00023304	noncoding	0.991
uc001vgk.2	noncoding	HFE	protein_coding	0.992
uc001vgk.2	noncoding	ENST00000584201	noncoding	0.995
uc001vgk.2	noncoding	EIF4A2	protein_coding	0.995
uc001vgk.2	noncoding	DNAJB4	protein_coding	0.991
NR_045634	noncoding	MMP21	protein_coding	0.991
NR_045634	noncoding	DNAJB4	protein_coding	0.996
NR_045634	noncoding	AMT	protein_coding	0.990
ENST00000567753	noncoding	ENST00000495228	noncoding	0.990

ENST00000567753	noncoding	ENST00000433460	noncoding	0.991
ENST00000567753	noncoding	ENST00000419627	noncoding	0.997
ENST00000567753	noncoding	ENST00000413645	noncoding	0.990
uc001zfw.1	noncoding	ENST00000571724	noncoding	0.991
uc001zfw.1	noncoding	ENST00000507571	noncoding	0.994
uc001zfw.1	noncoding	ENST00000435697	noncoding	0.992
uc001zfw.1	noncoding	CNRIP1	protein_coding	0.992
uc001zfw.1	noncoding	CIRBP	protein_coding	0.991
uc001zfw.1	noncoding	CD242780	noncoding	0.993
uc001zfw.1	noncoding	BEND5	protein_coding	0.993
uc001zfw.1	noncoding	B3GNT1	protein_coding	0.993
ENST00000495228	noncoding	DNAJC18	protein_coding	0.991
ENST00000495228	noncoding	CD242780	noncoding	0.992
ENST00000562501	noncoding	ENST00000512585	noncoding	0.992
ENST00000562501	noncoding	ENST00000413525	noncoding	0.996
ENST00000562501	noncoding	DPP4	protein_coding	0.994
ENST00000562191	noncoding	CRBN	protein_coding	0.991
ENST00000562191	noncoding	CNRIP1	protein_coding	0.991
ENST00000562191	noncoding	CIRBP	protein_coding	0.992
ENST00000508742	noncoding	CCT6B	protein_coding	0.994
uc003kkf.2	noncoding	uc003kke.2	noncoding	0.994
uc003kkf.2	noncoding	TCONS_00025895	noncoding	0.992
uc003kkf.2	noncoding	TCONS_00025350	noncoding	0.994
uc003kkf.2	noncoding	TCONS_00001581	noncoding	0.991
uc003kkf.2	noncoding	SPRR2F	protein_coding	0.993
uc003kkf.2	noncoding	SERHL2	protein_coding	0.991
uc003kkf.2	noncoding	MYL6B	protein_coding	0.990
uc003kkf.2	noncoding	ENST00000562501	noncoding	0.993
uc003kkf.2	noncoding	ENST00000512585	noncoding	0.997
uc003kkf.2	noncoding	ENST00000493124	noncoding	0.995
uc003kkf.2	noncoding	ENST00000471990	noncoding	0.994
uc003kkf.2	noncoding	ENST00000413525	noncoding	0.991
uc003kkf.2	noncoding	C6orf225	protein_coding	0.991
ENST00000453754	noncoding	ENST00000416111	noncoding	0.993
ENST00000453754	noncoding	DNAJC18	protein_coding	0.997
uc003hrz.1	noncoding	TCONS_00027009	noncoding	0.992
uc003hrz.1	noncoding	TCONS_00025350	noncoding	0.992
uc003hrz.1	noncoding	TCONS_00008647	noncoding	0.999
uc003hrz.1	noncoding	SLC16A14	protein_coding	0.992
uc003hrz.1	noncoding	SERHL2	protein_coding	0.995
uc003hrz.1	noncoding	NR_073094	noncoding	0.995
uc003hrz.1	noncoding	NR_033919	noncoding	0.990
uc003hrz.1	noncoding	NR_029389	noncoding	0.993
uc003hrz.1	noncoding	GLT8D2	protein_coding	0.991

uc003hrz.1	noncoding	ENST00000512585	noncoding	0.992
uc003hrz.1	noncoding	ENST00000506463	noncoding	0.992
uc003hrz.1	noncoding	ENST00000451070	noncoding	0.993
uc003hrz.1	noncoding	ENST00000444672	noncoding	0.991
uc003hrz.1	noncoding	DOK6	protein_coding	0.993
uc003hrz.1	noncoding	DNAJC12	protein_coding	0.994
ENST00000451070	noncoding	ENST00000444672	noncoding	0.998
ENST00000451070	noncoding	ARSE	protein_coding	0.995
ENST00000512585	noncoding	ENST00000493124	noncoding	0.995
ENST00000512585	noncoding	ENST00000471990	noncoding	0.995
ENST00000512585	noncoding	ENST00000451070	noncoding	0.991
ENST00000512585	noncoding	ENST00000444672	noncoding	0.990
ENST00000512585	noncoding	ENST00000429109	noncoding	0.995
ENST00000512585	noncoding	DOK6	protein_coding	0.992
ENST00000512585	noncoding	CDH8	protein_coding	0.991
NR_073094	noncoding	ENST00000569927	noncoding	0.993
NR_073094	noncoding	ENST00000506463	noncoding	0.992
ENST00000564660	noncoding	ENST00000453754	noncoding	0.992
ENST00000508718	noncoding	ENST00000435697	noncoding	0.994
ENST00000507571	noncoding	ENST00000435697	noncoding	0.996
ENST00000507571	noncoding	CNRIP1	protein_coding	0.994
ENST00000507571	noncoding	CIRBP	protein_coding	0.992
ENST00000507571	noncoding	BEND5	protein_coding	0.991
ENST00000507571	noncoding	B3GNT1	protein_coding	0.993
TCONS_00023799	noncoding	SPRR2F	protein_coding	0.991
TCONS_00023799	noncoding	SERGEF	protein_coding	0.991
TCONS_00023799	noncoding	NR_045634	noncoding	0.991
TCONS_00023799	noncoding	MGARP	protein_coding	0.994
TCONS_00023799	noncoding	METTL21C	protein_coding	0.996
TCONS_00023799	noncoding	ENST00000444672	noncoding	0.991
TCONS_00023799	noncoding	DNAJB4	protein_coding	0.990
TCONS_00023799	noncoding	CDH8	protein_coding	0.995
TCONS_00023799	noncoding	AMT	protein_coding	0.995
TCONS_00025895	noncoding	SERHL2	protein_coding	0.995
TCONS_00025895	noncoding	NSA2	protein_coding	0.994
TCONS_00025895	noncoding	GRIK2	protein_coding	0.992
TCONS_00025895	noncoding	ENST00000569927	noncoding	0.996
TCONS_00025895	noncoding	ENST00000562501	noncoding	0.991
TCONS_00025895	noncoding	ENST00000512585	noncoding	0.990
TCONS_00025895	noncoding	ENST00000506463	noncoding	0.993
TCONS_00025895	noncoding	ENST00000413525	noncoding	0.995
TCONS_00025895	noncoding	DYNC1I2	protein_coding	0.992
TCONS_00025895	noncoding	ARSE	protein_coding	0.994
ENST00000584201	noncoding	ENST00000571724	noncoding	0.991

ENST00000584201	noncoding	ENST00000508718	noncoding	0.992
ENST00000584201	noncoding	ENST00000507571	noncoding	0.991
ENST00000584201	noncoding	ENST00000435697	noncoding	0.994
ENST00000584201	noncoding	EIF4A2	protein_coding	0.991
ENST00000584201	noncoding	CNRIP1	protein_coding	0.991
ENST00000547777	noncoding	ENST00000509363	noncoding	0.994
ENST00000547777	noncoding	C6orf225	protein_coding	0.992
ENST00000429109	noncoding	EID3	protein_coding	0.991
ENST00000429109	noncoding	DOK6	protein_coding	0.997
ENST00000429109	noncoding	CDH8	protein_coding	0.993
ENST00000429109	noncoding	C6orf225	protein_coding	0.991
ENST00000442116	noncoding	DNAJB4	protein_coding	0.991
ENST00000442116	noncoding	CNRIP1	protein_coding	0.991
ENST00000571724	noncoding	ENST00000567753	noncoding	0.995
ENST00000571724	noncoding	ENST00000508718	noncoding	0.990
ENST00000571724	noncoding	ENST00000507571	noncoding	0.991
ENST00000571724	noncoding	ENST00000493124	noncoding	0.990
ENST00000571724	noncoding	ENST00000492209	noncoding	0.995
ENST00000571724	noncoding	ENST00000471990	noncoding	0.990
ENST00000571724	noncoding	ENST00000435697	noncoding	0.995
ENST00000571724	noncoding	ENST00000419627	noncoding	0.995
ENST00000571724	noncoding	CNRIP1	protein_coding	0.992
NR_027132	noncoding	HFM1	protein_coding	0.993
NR_027132	noncoding	ENST00000562191	noncoding	0.998
NR_027132	noncoding	ENST00000416111	noncoding	0.993
NR_027132	noncoding	CRBN	protein_coding	0.997
NR_027132	noncoding	CNRIP1	protein_coding	0.992
NR_027132	noncoding	CIRBP	protein_coding	0.994
NR_027132	noncoding	CD242780	noncoding	0.994
TCONS_00008647	noncoding	SPRR2F	protein_coding	0.992
TCONS_00008647	noncoding	SLC16A14	protein_coding	0.990
TCONS_00008647	noncoding	SERHL2	protein_coding	0.997
TCONS_00008647	noncoding	PRSS35	protein_coding	0.992
TCONS_00008647	noncoding	NSA2	protein_coding	0.992
TCONS_00008647	noncoding	NR_073094	noncoding	0.996
TCONS_00008647	noncoding	NR_029389	noncoding	0.994
TCONS_00008647	noncoding	GLT8D2	protein_coding	0.991
TCONS_00008647	noncoding	ENST00000569927	noncoding	0.994
TCONS_00008647	noncoding	ENST00000512585	noncoding	0.993
TCONS_00008647	noncoding	ENST00000506463	noncoding	0.994
TCONS_00008647	noncoding	ENST00000451070	noncoding	0.994
TCONS_00008647	noncoding	ENST00000444672	noncoding	0.991
TCONS_00008647	noncoding	DOK6	protein_coding	0.992
TCONS_00008647	noncoding	DNAJC12	protein_coding	0.992

ENST00000413525	noncoding	DYNC1I2	protein_coding	0.993
ENST00000444672	noncoding	ENST00000442116	noncoding	0.997
ENST00000444672	noncoding	DOK6	protein_coding	0.991
ENST00000444672	noncoding	DNAJB4	protein_coding	0.993
ENST00000444672	noncoding	CDH8	protein_coding	0.993
ENST00000444672	noncoding	BEND5	protein_coding	0.993
ENST00000444672	noncoding	B3GNT1	protein_coding	0.991
ENST00000444672	noncoding	ARSE	protein_coding	0.991
ENST00000562921	noncoding	ENST00000493124	noncoding	0.992
ENST00000562921	noncoding	ENST00000444672	noncoding	0.992
ENST00000562921	noncoding	ENST00000442116	noncoding	0.990
ENST00000562921	noncoding	ENST00000429109	noncoding	0.992
ENST00000562921	noncoding	DOK6	protein_coding	0.994
ENST00000562921	noncoding	CDH8	protein_coding	0.992
ENST00000562921	noncoding	B3GNT1	protein_coding	0.990
NR_040103	noncoding	NFIB	protein_coding	0.996
NR_040103	noncoding	HFE	protein_coding	0.993
NR_040103	noncoding	ENST00000565166	noncoding	0.990
TCONS_00001581	noncoding	SPRR2A	protein_coding	0.993
TCONS_00001581	noncoding	SLC16A14	protein_coding	0.994
TCONS_00001581	noncoding	PON3	protein_coding	0.993
TCONS_00001581	noncoding	NR_034004	noncoding	0.990
TCONS_00001581	noncoding	NR_026836	noncoding	0.992
TCONS_00001581	noncoding	FNDC4	protein_coding	0.991
TCONS_00001581	noncoding	ENST00000512585	noncoding	0.991
TCONS_00001581	noncoding	ENST00000493124	noncoding	0.994
TCONS_00001581	noncoding	ENST00000471990	noncoding	0.993
TCONS_00001581	noncoding	ENST00000429109	noncoding	0.995
TCONS_00001581	noncoding	DOK6	protein_coding	0.995
ENST00000494740	noncoding	ENST00000444375	noncoding	0.993
ENST00000494740	noncoding	CPQ	protein_coding	0.991
TCONS_00023304	noncoding	ENST00000584201	noncoding	0.991
ENST00000569927	noncoding	ENST00000562501	noncoding	0.992
ENST00000569927	noncoding	ENST00000506463	noncoding	0.995
ENST00000569927	noncoding	ENST00000413525	noncoding	0.995
ENST00000569927	noncoding	DYNC1I2	protein_coding	0.995
ENST00000569927	noncoding	ARSE	protein_coding	0.992
ENST00000416111	noncoding	DNAJC18	protein_coding	0.993
ENST00000416111	noncoding	CRBN	protein_coding	0.992
ENST00000416111	noncoding	CNRIP1	protein_coding	0.992
ENST00000416111	noncoding	CIRBP	protein_coding	0.995
ENST00000416111	noncoding	CD242780	noncoding	0.990
NR_034004	noncoding	ENST00000493124	noncoding	0.990
NR_034004	noncoding	ENST00000429109	noncoding	0.990

NR_034004	noncoding	ENST00000422799	noncoding	0.991
NR_034004	noncoding	DOK6	protein_coding	0.990
ENST00000493124	noncoding	ENST00000471990	noncoding	1.000
ENST00000493124	noncoding	ENST00000444672	noncoding	0.992
ENST00000493124	noncoding	ENST00000435697	noncoding	0.991
ENST00000493124	noncoding	ENST00000429109	noncoding	0.995
ENST00000493124	noncoding	ENST00000413645	noncoding	0.991
ENST00000493124	noncoding	DOK6	protein_coding	0.994
ENST00000493124	noncoding	DNAJB4	protein_coding	0.990
ENST00000493124	noncoding	CNRIP1	protein_coding	0.997
ENST00000493124	noncoding	CDH8	protein_coding	0.992
ENST00000493124	noncoding	BEND5	protein_coding	0.995
ENST00000493124	noncoding	B3GNT1	protein_coding	0.992
ENST00000419627	noncoding	CNRIP1	protein_coding	0.990
TCONS_00027009	noncoding	TCONS_00023799	noncoding	0.991
TCONS_00027009	noncoding	TCONS_00008647	noncoding	0.993
TCONS_00027009	noncoding	NR_073094	noncoding	0.993
TCONS_00027009	noncoding	NR_029389	noncoding	0.994
TCONS_00027009	noncoding	METTL21C	protein_coding	0.993
TCONS_00027009	noncoding	CDH8	protein_coding	0.992
NR_024423	noncoding	NFIB	protein_coding	0.992
NR_024423	noncoding	ENST00000584201	noncoding	0.994
ENST00000422799	noncoding	ENST00000412376	noncoding	0.992
ENST00000422799	noncoding	AF070541	noncoding	0.992
TCONS_00022431	noncoding	TCONS_00001581	noncoding	0.993
TCONS_00022431	noncoding	GPRASP1	protein_coding	0.991
TCONS_00022431	noncoding	FNDC4	protein_coding	0.991
TCONS_00022431	noncoding	ENST00000493124	noncoding	0.990
TCONS_00022431	noncoding	ENST00000415026	noncoding	0.990
TCONS_00022431	noncoding	ENST00000413645	noncoding	0.992
TCONS_00022431	noncoding	EIF2D	protein_coding	0.992
TCONS_00022431	noncoding	CNRIP1	protein_coding	0.991
ENST00000471990	noncoding	ENST00000444672	noncoding	0.991
ENST00000471990	noncoding	ENST00000435697	noncoding	0.990
ENST00000471990	noncoding	ENST00000429109	noncoding	0.995
ENST00000471990	noncoding	ENST00000413645	noncoding	0.992
ENST00000471990	noncoding	DOK6	protein_coding	0.993
ENST00000471990	noncoding	DNAJB4	protein_coding	0.991
ENST00000471990	noncoding	CNRIP1	protein_coding	0.996
ENST00000471990	noncoding	CDH8	protein_coding	0.992
ENST00000471990	noncoding	BEND5	protein_coding	0.993
ENST00000471990	noncoding	B3GNT1	protein_coding	0.990
ENST00000526694	noncoding	ENST00000444375	noncoding	0.994
ENST00000526694	noncoding	ENST00000415026	noncoding	0.991

NR_045029	noncoding	NR_040103	noncoding	0.991
NR_045029	noncoding	NFIB	protein_coding	0.994
NR_045029	noncoding	MGARP	protein_coding	0.998
NR_045029	noncoding	HFE	protein_coding	0.992
NR_045029	noncoding	ENST00000565166	noncoding	0.993
NR_045029	noncoding	AMT	protein_coding	0.995
TCONS_00025350	noncoding	TCONS_00008647	noncoding	0.994
TCONS_00025350	noncoding	TCONS_00001581	noncoding	0.991
TCONS_00025350	noncoding	SPRR2F	protein_coding	0.999
TCONS_00025350	noncoding	SLC35A5	protein_coding	0.991
TCONS_00025350	noncoding	SLC16A14	protein_coding	0.991
TCONS_00025350	noncoding	SERHL2	protein_coding	0.992
TCONS_00025350	noncoding	POLR3GL	protein_coding	0.992
TCONS_00025350	noncoding	NSA2	protein_coding	0.992
TCONS_00025350	noncoding	NR_033919	noncoding	0.997
TCONS_00025350	noncoding	NR_029389	noncoding	0.993
TCONS_00025350	noncoding	GLT8D2	protein_coding	0.994
TCONS_00025350	noncoding	ENST00000562921	noncoding	0.996
TCONS_00025350	noncoding	ENST00000512585	noncoding	0.996
TCONS_00025350	noncoding	ENST00000493124	noncoding	0.997
TCONS_00025350	noncoding	ENST00000471990	noncoding	0.995
TCONS_00025350	noncoding	ENST00000451070	noncoding	0.994
TCONS_00025350	noncoding	ENST00000444672	noncoding	0.996
TCONS_00025350	noncoding	ENST00000442116	noncoding	0.991
TCONS_00025350	noncoding	ENST00000429109	noncoding	0.995
TCONS_00025350	noncoding	DOK6	protein_coding	0.996
TCONS_00025350	noncoding	CNRIP1	protein_coding	0.991
TCONS_00025350	noncoding	CDH8	protein_coding	0.994
TCONS_00025350	noncoding	BEND5	protein_coding	0.994
TCONS_00025350	noncoding	B3GNT1	protein_coding	0.992
NR_033919	noncoding	NR_029389	noncoding	0.993
NR_033919	noncoding	MAB21L1	protein_coding	0.992
NR_033919	noncoding	GLT8D2	protein_coding	0.997
NR_033919	noncoding	ENST00000562921	noncoding	0.994
NR_033919	noncoding	ENST00000493124	noncoding	0.995
NR_033919	noncoding	ENST00000471990	noncoding	0.993
NR_033919	noncoding	ENST00000451070	noncoding	0.994
NR_033919	noncoding	ENST00000444672	noncoding	0.998
NR_033919	noncoding	ENST00000442116	noncoding	0.996
NR_033919	noncoding	DOK6	protein_coding	0.995
NR_033919	noncoding	CNRIP1	protein_coding	0.993
NR_033919	noncoding	CIRBP	protein_coding	0.992
NR_033919	noncoding	CDH8	protein_coding	0.990
NR_033919	noncoding	BEND5	protein_coding	0.994

NR_033919	noncoding	B3GNT1	protein_coding	0.992
uc010ocf.2	noncoding	uc003kkf.2	noncoding	0.991
uc010ocf.2	noncoding	TRABD2A	protein_coding	0.993
uc010ocf.2	noncoding	TCONS_00025895	noncoding	0.991
uc010ocf.2	noncoding	SPRR2F	protein_coding	0.992
uc010ocf.2	noncoding	GRIK2	protein_coding	0.990
uc010ocf.2	noncoding	FEZ2	protein_coding	0.993
uc010ocf.2	noncoding	FAM18B1	protein_coding	0.994
uc010ocf.2	noncoding	ENST00000508742	noncoding	0.995
uc010ocf.2	noncoding	ENST00000494740	noncoding	0.993
uc010ocf.2	noncoding	DYNC1I2	protein_coding	0.993
NR_026836	noncoding	MXRA7	protein_coding	0.993
NR_026836	noncoding	ENST00000509363	noncoding	0.991
NR_026836	noncoding	ENST00000429109	noncoding	0.991
NR_026836	noncoding	EID3	protein_coding	0.994
NR_026836	noncoding	DNAJC12	protein_coding	0.993
ENST00000433460	noncoding	ENST00000419627	noncoding	0.994
ENST00000433460	noncoding	C6orf225	protein_coding	0.997
ENST00000554749	noncoding	ENST00000466225	noncoding	0.994
ENST00000554749	noncoding	ENST00000429109	noncoding	0.992
ENST00000554749	noncoding	EID3	protein_coding	0.990
uc003lta.2	noncoding	TCONS_00022431	noncoding	0.997
uc003lta.2	noncoding	TCONS_00001581	noncoding	0.993
uc003lta.2	noncoding	NR_047522	noncoding	0.992
uc003lta.2	noncoding	NR_033919	noncoding	0.991
uc003lta.2	noncoding	NR_027133	noncoding	0.992
uc003lta.2	noncoding	NR_027132	noncoding	0.992
uc003lta.2	noncoding	MAB21L1	protein_coding	0.992
uc003lta.2	noncoding	GLT8D2	protein_coding	0.992
uc003lta.2	noncoding	FNDC4	protein_coding	0.994
uc003lta.2	noncoding	ENST00000562191	noncoding	0.993
uc003lta.2	noncoding	ENST00000493124	noncoding	0.992
uc003lta.2	noncoding	ENST00000471990	noncoding	0.991
uc003lta.2	noncoding	ENST00000415026	noncoding	0.994
uc003lta.2	noncoding	ENST00000413645	noncoding	0.992
uc003lta.2	noncoding	CNRIP1	protein_coding	0.994
ENST00000509363	noncoding	ENST00000429109	noncoding	0.992
ENST00000509363	noncoding	EID3	protein_coding	0.991
ENST00000556899	noncoding	ENST00000495228	noncoding	0.995
ENST00000556899	noncoding	AKAP12	protein_coding	0.995
NR_029389	noncoding	GLT8D2	protein_coding	0.997
NR_029389	noncoding	ENST00000506463	noncoding	0.990
NR_029389	noncoding	ENST00000451070	noncoding	0.996
NR_029389	noncoding	ENST00000444672	noncoding	0.996

NR_029389	noncoding	ENST00000442116	noncoding	0.991
NR_029389	noncoding	CDH8	protein_coding	0.991
NR_029389	noncoding	ARSE	protein_coding	0.992
NR_039993	noncoding	NR_026836	noncoding	0.995
NR_039993	noncoding	MXRA7	protein_coding	0.998
NR_039993	noncoding	ENST00000554749	noncoding	0.993
NR_039993	noncoding	ENST00000429109	noncoding	0.993
NR_039993	noncoding	EID3	protein_coding	0.992
ENST00000506463	noncoding	ENST00000451070	noncoding	0.996
ENST00000506463	noncoding	ENST00000444672	noncoding	0.990
ENST00000506463	noncoding	ARSE	protein_coding	0.997
uc003kke.2	noncoding	uc003hrz.1	noncoding	0.992
uc003kke.2	noncoding	TCONS_00027009	noncoding	0.992
uc003kke.2	noncoding	TCONS_00025895	noncoding	0.991
uc003kke.2	noncoding	TCONS_00025350	noncoding	0.995
uc003kke.2	noncoding	TCONS_00008647	noncoding	0.993
uc003kke.2	noncoding	SPRR2F	protein_coding	0.994
uc003kke.2	noncoding	SLC16A14	protein_coding	0.993
uc003kke.2	noncoding	SIRT4	protein_coding	0.992
uc003kke.2	noncoding	SERHL2	protein_coding	0.993
uc003kke.2	noncoding	NSA2	protein_coding	0.992
uc003kke.2	noncoding	NR_073094	noncoding	0.992
uc003kke.2	noncoding	NR_029389	noncoding	0.990
uc003kke.2	noncoding	MXRA7	protein_coding	0.990
uc003kke.2	noncoding	ENST00000569927	noncoding	0.990
uc003kke.2	noncoding	ENST00000512585	noncoding	0.999
uc003kke.2	noncoding	ENST00000493124	noncoding	0.992
uc003kke.2	noncoding	ENST00000471990	noncoding	0.993
uc003kke.2	noncoding	ENST00000451070	noncoding	0.991
uc003kke.2	noncoding	ENST00000429109	noncoding	0.994
uc003kke.2	noncoding	DOK6	protein_coding	0.990
uc003kke.2	noncoding	CDH8	protein_coding	0.994
ENST00000413645	noncoding	EIF2D	protein_coding	0.991
ENST00000413645	noncoding	CNRIP1	protein_coding	0.994
ENST00000413645	noncoding	AKAP12	protein_coding	0.992

Interaction: the values of interation coeffient indicates the degree of two gene co-expression. The larger of absolute value, the stronger of co-expression.Positive represent a positive regulationand negetive indicate a negetive regulation.

Table S5B. The property of mRNA and lncRNA of profile NO.15

Genesymbol/lncRNA	clusteringCoefficient	degree	type
SPRR2F	0.368	35	protein_coding
ENST00000493124	0.369	32	noncoding
TCONS_00025350	0.452	31	noncoding
ENST00000444672	0.385	31	noncoding
CNRIP1	0.271	31	protein_coding
ENST00000471990	0.384	29	noncoding
NR_033919	0.413	27	noncoding
CDH8	0.434	26	protein_coding
uc003kke.2	0.431	26	noncoding
ENST00000429109	0.397	26	noncoding
ENST00000512585	0.467	25	noncoding
TDRD10	0.447	25	protein_coding
SERHL2	0.399	23	protein_coding
SLC16A14	0.391	23	protein_coding
DOK6	0.580	22	protein_coding
CD46	0.381	22	protein_coding
TCONS_00008647	0.467	21	noncoding
PPP3CB	0.367	21	protein_coding
ZNF385B	0.437	20	protein_coding
VIT	0.432	20	protein_coding
GLT8D2	0.526	19	protein_coding
BEND5	0.491	19	protein_coding
NR_029389	0.450	19	noncoding
POLR3GL	0.450	19	protein_coding
GPRASP1	0.351	19	protein_coding
TCONS_00001581	0.345	19	noncoding
CIRBP	0.386	18	protein_coding
uc003lta.2	0.320	18	noncoding
ENST00000451070	0.574	17	noncoding
B3GNT1	0.507	17	protein_coding
MGARP	0.346	17	protein_coding
SPRR2A	0.287	17	protein_coding
MAB21L1	0.250	17	protein_coding
ENST00000442116	0.558	16	noncoding
uc003hrz.1	0.517	16	noncoding
NSA2	0.458	16	protein_coding
uc003kkf.2	0.450	16	noncoding
DNAJB4	0.417	16	protein_coding
ENST00000571724	0.383	16	noncoding
ENST00000435697	0.375	16	noncoding
ENST00000507571	0.358	16	noncoding
CNBP	0.350	16	protein_coding

ENST00000584201	0.233	16	noncoding
ENST00000562921	0.724	15	noncoding
RDX	0.514	15	protein_coding
ZNF323	0.381	15	protein_coding
ENST00000494740	0.385	14	noncoding
ZC4H2	0.374	14	protein_coding
ENST00000569927	0.538	13	noncoding
TCONS_00025895	0.526	13	noncoding
TCONS_00023799	0.423	13	noncoding
TCONS_00022431	0.346	13	noncoding
NR_027133	0.346	13	noncoding
SERGEF	0.321	13	protein_coding
MXRA7	0.591	12	protein_coding
NGF	0.470	12	protein_coding
NR_026836	0.470	12	noncoding
ENST00000413645	0.424	12	noncoding
ENST00000562501	0.409	12	noncoding
NR_034004	0.379	12	noncoding
uc010ocf.2	0.242	12	noncoding
ENST00000506463	0.618	11	noncoding
SLC35A5	0.564	11	protein_coding
uc001zfw.1	0.545	11	noncoding
SULF2	0.382	11	protein_coding
SPRR2E	0.345	11	protein_coding
ENST00000416111	0.327	11	noncoding
CD242780	0.255	11	noncoding
NR1H4	0.255	11	protein_coding
SLC25A27	0.200	11	protein_coding
ARSE	0.533	10	protein_coding
TCONS_00027009	0.489	10	noncoding
NR_045029	0.489	10	noncoding
NR_027132	0.489	10	noncoding
ENST00000509363	0.444	10	noncoding
EID3	0.750	9	protein_coding
OLFM1	0.750	9	protein_coding
ENST00000413525	0.694	9	noncoding
ZBTB20	0.556	9	protein_coding
HFE	0.472	9	protein_coding
SCARB1	0.444	9	protein_coding
ZNF181	0.389	9	protein_coding
VWC2	0.333	9	protein_coding
PLEKHA5	0.306	9	protein_coding
AMT	0.250	9	protein_coding
GRIK2	0.750	8	protein_coding

DYNC1I2	0.714	8	protein_coding
NR_073094	0.679	8	noncoding
ENST00000554749	0.536	8	noncoding
SETDB2	0.500	8	protein_coding
VEZT	0.500	8	protein_coding
TDGF1	0.464	8	protein_coding
TBC1D8B	0.429	8	protein_coding
uc001vgk.2	0.393	8	noncoding
ENST00000453754	0.357	8	noncoding
NR_039993	0.905	7	noncoding
FEZ2	0.810	7	protein_coding
FABP4	0.762	7	protein_coding
HNRNPA1L2	0.667	7	protein_coding
ENST00000419627	0.667	7	noncoding
CPQ	0.619	7	protein_coding
NFIB	0.571	7	protein_coding
ENST00000508718	0.524	7	noncoding
ZNF599	0.524	7	protein_coding
HFM1	0.429	7	protein_coding
STAMBPL1	0.429	7	protein_coding
EIF2D	0.333	7	protein_coding
ENST00000495228	0.095	7	noncoding
NR_040103	0.733	6	noncoding
PRSS35	0.733	6	protein_coding
ENST00000562191	0.733	6	noncoding
EIF4A2	0.600	6	protein_coding
FAM156B	0.600	6	protein_coding
SIRT4	0.600	6	protein_coding
CRBN	0.533	6	protein_coding
FHL5	0.533	6	protein_coding
ENST00000444375	0.467	6	noncoding
OBSL1	0.467	6	protein_coding
NTF3	0.467	6	protein_coding
ENST00000415026	0.467	6	noncoding
FNDC4	0.467	6	protein_coding
ENST00000422799	0.467	6	noncoding
PON3	0.400	6	protein_coding
ENST00000567753	0.400	6	noncoding
ZMAT1	0.333	6	protein_coding
SF1	0.333	6	protein_coding
C6orf225	0.200	6	protein_coding
NR_047522	0.600	5	noncoding
ENST00000565166	0.600	5	noncoding
FAM18B1	0.500	5	protein_coding

AKAP12	0.500	5	protein_coding
EEF1A1	0.500	5	protein_coding
KIF16B	0.500	5	protein_coding
NR_045634	0.500	5	noncoding
ENST00000508742	0.400	5	noncoding
DNAJC18	0.400	5	protein_coding
DNAJC12	0.400	5	protein_coding
TRABD2A	0.200	5	protein_coding
MYL6B	1.000	4	protein_coding
ENST00000512246	0.833	4	noncoding
ENST00000412376	0.667	4	noncoding
ENST00000466225	0.667	4	noncoding
SPG7	0.667	4	protein_coding
MMP21	0.500	4	protein_coding
ENST00000433460	0.333	4	noncoding
METTL21C	0.333	4	protein_coding
NUDT11	0.333	4	protein_coding
ZNF518B	0.333	4	protein_coding
AF070541	0.333	4	noncoding
LIN52	0.167	4	protein_coding
ENST00000515376	1.000	3	noncoding
YTHDC1	1.000	3	protein_coding
HNRNPH3	1.000	3	protein_coding
PCGF6	1.000	3	protein_coding
WNT5B	0.667	3	protein_coding
ENST00000564660	0.667	3	noncoding
DFNA5	0.667	3	protein_coding
ENST00000547777	0.333	3	noncoding
NR_024423	0.333	3	noncoding
ENST00000526694	0.333	3	noncoding
KANSL1L	0.333	3	protein_coding
PHF10	0.333	3	protein_coding
OLFML3	1.000	2	protein_coding
TTC8	1.000	2	protein_coding
TCONS_00023304	1.000	2	noncoding
MAOA	1.000	2	protein_coding
DPP4	1.000	2	protein_coding
CCT6B	1.000	2	protein_coding
ENST00000556899	0.000	2	noncoding
ENST00000492209	0.000	1	noncoding
HARS2	0.000	1	protein_coding
NKAPL	0.000	1	protein_coding

Clustering Coefficient: The greater of clustering coefficient, the closer relationship between genes of co-expression; **Degree:** It indicates the number of the gene interact with this gene in this co-

expression networks.

Table S6A. The relation of mRNA and lncRNA of profile NO. 9

Gene1/lncRNA1	type 1	Gene2/lncRNA2	type 2	interaction
HNRNPH3	protein_coding	FERMT2	protein_coding	0.992
HNRNPH3	protein_coding	uc022atk.1	noncoding	0.988
HNRNPH3	protein_coding	TNMD	protein_coding	0.994
HNRNPH3	protein_coding	STT3B	protein_coding	0.987
HNRNPH3	protein_coding	STAR	protein_coding	0.982
HNRNPH3	protein_coding	RHOB	protein_coding	0.982
HNRNPH3	protein_coding	PMP22	protein_coding	0.980
HNRNPH3	protein_coding	OAT	protein_coding	0.989
HNRNPH3	protein_coding	NR_046473	noncoding	0.990
HNRNPH3	protein_coding	NR_046464	noncoding	0.988
HNRNPH3	protein_coding	NR_027158	noncoding	0.981
HNRNPH3	protein_coding	HADHA	protein_coding	0.982
HNRNPH3	protein_coding	HAAO	protein_coding	0.991
HNRNPH3	protein_coding	ESD	protein_coding	0.986
HNRNPH3	protein_coding	EPHX1	protein_coding	0.984
HNRNPH3	protein_coding	ENST00000564371	noncoding	0.990
HNRNPH3	protein_coding	ENST00000520714	noncoding	0.983
HNRNPH3	protein_coding	ENST00000436013	noncoding	0.990
HNRNPH3	protein_coding	DCTN4	protein_coding	0.981
HNRNPH3	protein_coding	COL6A2	protein_coding	0.984
HNRNPH3	protein_coding	CD302	protein_coding	0.993
HNRNPH3	protein_coding	C10orf82	protein_coding	0.988
HNRNPH3	protein_coding	ANGPTL5	protein_coding	0.988
PPP1R8	protein_coding	CLMP	protein_coding	0.986
PPP1R8	protein_coding	ALDH1L1	protein_coding	0.992
FBXO38	protein_coding	CAMLG	protein_coding	0.982
FBXO38	protein_coding	ENST00000578315	noncoding	0.981
FBXO38	protein_coding	ENST00000568835	noncoding	0.988
FBXO38	protein_coding	ENST00000564371	noncoding	0.988
FBXO38	protein_coding	ENST00000549527	noncoding	0.983
FBXO38	protein_coding	ENST00000510244	noncoding	0.989
FBXO38	protein_coding	CPXM1	protein_coding	0.986
FBXO38	protein_coding	CLMP	protein_coding	0.981
FBXO38	protein_coding	CD302	protein_coding	0.984
FBXO38	protein_coding	ALDH1L1	protein_coding	0.984
FBXO38	protein_coding	ACAD9	protein_coding	0.987
ZFP82	protein_coding	CAMLG	protein_coding	0.986
ZFP82	protein_coding	TNMD	protein_coding	0.982
ZFP82	protein_coding	SUPT16H	protein_coding	0.984
ZFP82	protein_coding	RHOB	protein_coding	0.982
ZFP82	protein_coding	OAT	protein_coding	0.982
ZFP82	protein_coding	NR_038402	noncoding	0.987

ZFP82	protein_coding	IFT88	protein_coding	0.993
ZFP82	protein_coding	HADHA	protein_coding	0.984
ZFP82	protein_coding	HAAO	protein_coding	0.987
ZFP82	protein_coding	EPHX1	protein_coding	0.987
ZFP82	protein_coding	ENST00000578315	noncoding	0.981
ZFP82	protein_coding	ENST00000568835	noncoding	0.983
ZFP82	protein_coding	ENST00000564371	noncoding	0.983
ZFP82	protein_coding	ENST00000436013	noncoding	0.985
ZFP82	protein_coding	CD302	protein_coding	0.988
ZFP82	protein_coding	C10orf82	protein_coding	0.990
OAT	protein_coding	NR_046464	noncoding	0.983
OAT	protein_coding	NR_045021	noncoding	0.987
OAT	protein_coding	NR_038402	noncoding	0.989
OAT	protein_coding	NR_027158	noncoding	0.982
OAT	protein_coding	HADHA	protein_coding	0.980
OAT	protein_coding	HAAO	protein_coding	0.984
OAT	protein_coding	FBXO38	protein_coding	0.991
OAT	protein_coding	EPHX1	protein_coding	0.981
OAT	protein_coding	ENST00000564371	noncoding	0.991
OAT	protein_coding	ENST00000549527	noncoding	0.982
OAT	protein_coding	ENST00000484836	noncoding	0.981
OAT	protein_coding	CPXM1	protein_coding	0.983
OAT	protein_coding	CLMP	protein_coding	0.984
OAT	protein_coding	CD302	protein_coding	0.996
OAT	protein_coding	C10orf82	protein_coding	0.985
OAT	protein_coding	BAG2	protein_coding	0.982
ASH2L	protein_coding	COL6A2	protein_coding	0.988
ASH2L	protein_coding	CD302	protein_coding	0.982
OCRL	protein_coding	TAB2	protein_coding	0.988
OCRL	protein_coding	PPP1R8	protein_coding	0.994
OCRL	protein_coding	PMP22	protein_coding	0.984
OCRL	protein_coding	NR_046464	noncoding	0.983
OCRL	protein_coding	NR_015447	noncoding	0.986
OCRL	protein_coding	ENST00000564371	noncoding	0.984
OCRL	protein_coding	COL6A2	protein_coding	0.984
OCRL	protein_coding	BAG2	protein_coding	0.987
OCRL	protein_coding	ALDH1L1	protein_coding	0.988
CNBP	protein_coding	uc022atk.1	noncoding	0.985
CNBP	protein_coding	SUPT16H	protein_coding	0.980
CNBP	protein_coding	STT3B	protein_coding	0.995
CNBP	protein_coding	STAR	protein_coding	0.980
CNBP	protein_coding	NR_045021	noncoding	0.981
CNBP	protein_coding	NR_027158	noncoding	0.991
CNBP	protein_coding	MAF	protein_coding	0.982

CNBP	protein_coding	HADHA	protein_coding	0.981
CNBP	protein_coding	HAAO	protein_coding	0.988
CNBP	protein_coding	EPHX1	protein_coding	0.988
CNBP	protein_coding	ENST00000484836	noncoding	0.989
CNBP	protein_coding	CPXM1	protein_coding	0.981
CNBP	protein_coding	C10orf82	protein_coding	0.983
HADHA	protein_coding	HAAO	protein_coding	0.986
HADHA	protein_coding	EPHX1	protein_coding	0.990
HADHA	protein_coding	ENST00000564371	noncoding	0.983
HADHA	protein_coding	ENST00000551107	noncoding	0.987
HADHA	protein_coding	ENST00000484836	noncoding	0.989
HADHA	protein_coding	ENST00000436013	noncoding	0.986
HADHA	protein_coding	CD302	protein_coding	0.986
HADHA	protein_coding	C10orf82	protein_coding	0.995
HADHA	protein_coding	ANGPTL5	protein_coding	0.982
HAAO	protein_coding	EPHX1	protein_coding	0.996
HAAO	protein_coding	ENST00000436013	noncoding	0.989
HAAO	protein_coding	CD302	protein_coding	0.987
HAAO	protein_coding	C10orf82	protein_coding	0.994
HAAO	protein_coding	ANGPTL5	protein_coding	0.984
TAB2	protein_coding	SYNGR1	protein_coding	0.989
TAB2	protein_coding	SUPT16H	protein_coding	0.987
TAB2	protein_coding	RHOB	protein_coding	0.993
TAB2	protein_coding	RABGAP1L	protein_coding	0.984
TAB2	protein_coding	PPP1R8	protein_coding	0.990
TAB2	protein_coding	PMP22	protein_coding	0.990
TAB2	protein_coding	OAT	protein_coding	0.994
TAB2	protein_coding	NR_046464	noncoding	0.982
TAB2	protein_coding	NR_045021	noncoding	0.988
TAB2	protein_coding	NR_038402	noncoding	0.982
TAB2	protein_coding	LRRTM3	protein_coding	0.982
TAB2	protein_coding	FBXO38	protein_coding	0.992
TAB2	protein_coding	ENST00000564371	noncoding	0.989
TAB2	protein_coding	ENST00000549527	noncoding	0.985
TAB2	protein_coding	ENST00000484836	noncoding	0.980
TAB2	protein_coding	CPXM1	protein_coding	0.985
TAB2	protein_coding	CLMP	protein_coding	0.992
TAB2	protein_coding	CD302	protein_coding	0.988
TAB2	protein_coding	BAG2	protein_coding	0.985
TAB2	protein_coding	ALDH1L1	protein_coding	0.989
STAR	protein_coding	NR_046473	noncoding	0.994
STAR	protein_coding	NR_027158	noncoding	0.986
STAR	protein_coding	MAF	protein_coding	0.988
STAR	protein_coding	HADHA	protein_coding	0.985

STAR	protein_coding	HAAO	protein_coding	0.985
STAR	protein_coding	C10orf82	protein_coding	0.984
STAR	protein_coding	ABI1	protein_coding	0.981
RHOB	protein_coding	RABGAP1L	protein_coding	0.986
RHOB	protein_coding	PMP22	protein_coding	0.984
RHOB	protein_coding	OAT	protein_coding	0.995
RHOB	protein_coding	NR_049782	noncoding	0.983
RHOB	protein_coding	NR_046464	noncoding	0.983
RHOB	protein_coding	NR_045021	noncoding	0.996
RHOB	protein_coding	NR_038402	noncoding	0.981
RHOB	protein_coding	NR_027158	noncoding	0.988
RHOB	protein_coding	HADHA	protein_coding	0.987
RHOB	protein_coding	FBXO38	protein_coding	0.992
RHOB	protein_coding	ENST00000564371	noncoding	0.992
RHOB	protein_coding	ENST00000551107	noncoding	0.986
RHOB	protein_coding	ENST00000549527	noncoding	0.993
RHOB	protein_coding	ENST00000484836	noncoding	0.989
RHOB	protein_coding	CPXM1	protein_coding	0.989
RHOB	protein_coding	CLMP	protein_coding	0.992
RHOB	protein_coding	CDIPT	protein_coding	0.986
RHOB	protein_coding	CD302	protein_coding	0.996
RHOB	protein_coding	C10orf82	protein_coding	0.986
RHOB	protein_coding	BAG2	protein_coding	0.984
RHOB	protein_coding	ALDH1L1	protein_coding	0.983
RHOB	protein_coding	ACAD9	protein_coding	0.987
OS9	protein_coding	OCRL	protein_coding	0.991
OS9	protein_coding	ZNF232	protein_coding	0.988
OS9	protein_coding	TAB2	protein_coding	0.985
OS9	protein_coding	SYNGR1	protein_coding	0.982
OS9	protein_coding	SUPT16H	protein_coding	0.980
OS9	protein_coding	RHOB	protein_coding	0.983
OS9	protein_coding	PPP1R8	protein_coding	0.985
OS9	protein_coding	PMP22	protein_coding	0.988
OS9	protein_coding	OAT	protein_coding	0.981
OS9	protein_coding	KLHL20	protein_coding	0.983
OS9	protein_coding	FBXO38	protein_coding	0.988
OS9	protein_coding	ENST00000578315	noncoding	0.981
OS9	protein_coding	ENST00000564371	noncoding	0.983
OS9	protein_coding	ENST00000549527	noncoding	0.982
OS9	protein_coding	ENST00000510244	noncoding	0.980
OS9	protein_coding	CD302	protein_coding	0.981
OS9	protein_coding	BAG2	protein_coding	0.988
OS9	protein_coding	ACAD9	protein_coding	0.989
ZNF232	protein_coding	TAB2	protein_coding	0.991

ZNF232	protein_coding	SYNGR1	protein_coding	0.985
ZNF232	protein_coding	SUPT16H	protein_coding	0.988
ZNF232	protein_coding	RHOB	protein_coding	0.995
ZNF232	protein_coding	PMP22	protein_coding	0.987
ZNF232	protein_coding	OAT	protein_coding	0.990
ZNF232	protein_coding	NR_049782	noncoding	0.988
ZNF232	protein_coding	NR_045021	noncoding	0.988
ZNF232	protein_coding	FBXO38	protein_coding	0.990
ZNF232	protein_coding	ENST00000564371	noncoding	0.981
ZNF232	protein_coding	ENST00000549527	noncoding	0.997
ZNF232	protein_coding	CLMP	protein_coding	0.987
ZNF232	protein_coding	CDIPT	protein_coding	0.981
ZNF232	protein_coding	CD302	protein_coding	0.989
ZNF232	protein_coding	BAG2	protein_coding	0.991
ZNF232	protein_coding	ACAD9	protein_coding	0.986
KLHL20	protein_coding	GSTM3	protein_coding	0.980
KLHL20	protein_coding	FBXO38	protein_coding	0.981
KLHL20	protein_coding	ENST00000578315	noncoding	0.991
KLHL20	protein_coding	ENST00000568835	noncoding	0.988
KLHL20	protein_coding	ENST00000510244	noncoding	0.996
PRPF31	protein_coding	ENST00000412951	noncoding	0.985
LRRTM3	protein_coding	GSTM3	protein_coding	0.989
LRRTM3	protein_coding	FBXO38	protein_coding	0.982
LRRTM3	protein_coding	ALDH1L1	protein_coding	0.985
CDIPT	protein_coding	ABI1	protein_coding	0.990
COL6A2	protein_coding	CD302	protein_coding	0.985
COL6A2	protein_coding	BAG2	protein_coding	0.989
VPS51	protein_coding	CAMLG	protein_coding	0.991
VPS51	protein_coding	EPHX1	protein_coding	0.987
VPS51	protein_coding	ENST00000436013	noncoding	0.981
FERMT2	protein_coding	ZNF232	protein_coding	0.982
FERMT2	protein_coding	TNMD	protein_coding	0.982
FERMT2	protein_coding	TAB2	protein_coding	0.981
FERMT2	protein_coding	SUPT16H	protein_coding	0.982
FERMT2	protein_coding	RHOB	protein_coding	0.987
FERMT2	protein_coding	PRPF31	protein_coding	0.981
FERMT2	protein_coding	PMP22	protein_coding	0.986
FERMT2	protein_coding	OAT	protein_coding	0.989
FERMT2	protein_coding	NR_046473	noncoding	0.982
FERMT2	protein_coding	NR_046464	noncoding	0.992
FERMT2	protein_coding	HAAO	protein_coding	0.982
FERMT2	protein_coding	ENST00000564371	noncoding	0.985
FERMT2	protein_coding	ENST00000520714	noncoding	0.985
FERMT2	protein_coding	DCTN4	protein_coding	0.981

FERMT2	protein_coding	COL6A2	protein_coding	0.985
FERMT2	protein_coding	CD302	protein_coding	0.996
FERMT2	protein_coding	C10orf82	protein_coding	0.983
FERMT2	protein_coding	BAG2	protein_coding	0.983
STT3B	protein_coding	STAR	protein_coding	0.990
STT3B	protein_coding	RHOB	protein_coding	0.983
STT3B	protein_coding	OAT	protein_coding	0.980
STT3B	protein_coding	NR_046473	noncoding	0.991
STT3B	protein_coding	NR_045021	noncoding	0.987
STT3B	protein_coding	NR_027158	noncoding	0.996
STT3B	protein_coding	MAF	protein_coding	0.988
STT3B	protein_coding	HADHA	protein_coding	0.991
STT3B	protein_coding	HAAO	protein_coding	0.991
STT3B	protein_coding	EPHX1	protein_coding	0.992
STT3B	protein_coding	ENST00000564371	noncoding	0.984
STT3B	protein_coding	ENST00000551107	noncoding	0.985
STT3B	protein_coding	ENST00000484836	noncoding	0.993
STT3B	protein_coding	ENST00000436013	noncoding	0.989
STT3B	protein_coding	CPXM1	protein_coding	0.982
STT3B	protein_coding	CD302	protein_coding	0.984
STT3B	protein_coding	C10orf82	protein_coding	0.989
STT3B	protein_coding	ANGPTL5	protein_coding	0.981
RABGAP1L	protein_coding	CAMLG	protein_coding	0.991
RABGAP1L	protein_coding	OAT	protein_coding	0.983
RABGAP1L	protein_coding	NR_045021	noncoding	0.988
RABGAP1L	protein_coding	NR_038402	noncoding	0.982
RABGAP1L	protein_coding	LRRTM3	protein_coding	0.980
RABGAP1L	protein_coding	GSTM3	protein_coding	0.990
RABGAP1L	protein_coding	FBXO38	protein_coding	0.992
RABGAP1L	protein_coding	ENST00000568835	noncoding	0.983
RABGAP1L	protein_coding	ENST00000564371	noncoding	0.985
RABGAP1L	protein_coding	ENST00000484836	noncoding	0.985
RABGAP1L	protein_coding	CPXM1	protein_coding	0.995
RABGAP1L	protein_coding	CLMP	protein_coding	0.981
RABGAP1L	protein_coding	ALDH1L1	protein_coding	0.984
PMP22	protein_coding	OAT	protein_coding	0.993
PMP22	protein_coding	NR_038402	noncoding	0.986
PMP22	protein_coding	FBXO38	protein_coding	0.984
PMP22	protein_coding	ENST00000564371	noncoding	0.981
PMP22	protein_coding	COL6A2	protein_coding	0.985
PMP22	protein_coding	CD302	protein_coding	0.990
PMP22	protein_coding	BAG2	protein_coding	0.989
C10orf82	protein_coding	ANGPTL5	protein_coding	0.981
EPHX1	protein_coding	CAMLG	protein_coding	0.989

EPHX1	protein_coding	ENST00000436013	noncoding	0.991
EPHX1	protein_coding	CD302	protein_coding	0.981
EPHX1	protein_coding	C10orf82	protein_coding	0.993
EPHX1	protein_coding	ANGPTL5	protein_coding	0.986
ESD	protein_coding	COL6A2	protein_coding	0.990
GSTM3	protein_coding	CAMLG	protein_coding	0.985
GSTM3	protein_coding	FBXO38	protein_coding	0.989
GSTM3	protein_coding	ENST00000568835	noncoding	0.989
GSTM3	protein_coding	ENST00000564371	noncoding	0.980
GSTM3	protein_coding	ENST00000510244	noncoding	0.985
MAF	protein_coding	ENST00000520714	noncoding	0.990
MAF	protein_coding	ENST00000484836	noncoding	0.993
MAF	protein_coding	DCTN4	protein_coding	0.987
MAF	protein_coding	CLMP	protein_coding	0.986
MAF	protein_coding	CDIPT	protein_coding	0.989
MAF	protein_coding	ABI1	protein_coding	0.987
RAB3GAP1	protein_coding	OS9	protein_coding	0.990
RAB3GAP1	protein_coding	OCRL	protein_coding	0.983
RAB3GAP1	protein_coding	FERMT2	protein_coding	0.986
RAB3GAP1	protein_coding	ZNF232	protein_coding	0.992
RAB3GAP1	protein_coding	TAB2	protein_coding	0.990
RAB3GAP1	protein_coding	RHOB	protein_coding	0.986
RAB3GAP1	protein_coding	PMP22	protein_coding	0.998
RAB3GAP1	protein_coding	OAT	protein_coding	0.991
RAB3GAP1	protein_coding	FBXO38	protein_coding	0.985
RAB3GAP1	protein_coding	ENST00000549527	noncoding	0.982
RAB3GAP1	protein_coding	COL6A2	protein_coding	0.980
RAB3GAP1	protein_coding	CD302	protein_coding	0.988
RAB3GAP1	protein_coding	BAG2	protein_coding	0.992
SUPT16H	protein_coding	STT3B	protein_coding	0.986
SUPT16H	protein_coding	RHOB	protein_coding	0.997
SUPT16H	protein_coding	RABGAP1L	protein_coding	0.990
SUPT16H	protein_coding	OAT	protein_coding	0.989
SUPT16H	protein_coding	NR_045021	noncoding	0.994
SUPT16H	protein_coding	NR_038402	noncoding	0.980
SUPT16H	protein_coding	NR_027158	noncoding	0.987
SUPT16H	protein_coding	HADHA	protein_coding	0.987
SUPT16H	protein_coding	FBXO38	protein_coding	0.991
SUPT16H	protein_coding	ENST00000568835	noncoding	0.981
SUPT16H	protein_coding	ENST00000564371	noncoding	0.995
SUPT16H	protein_coding	ENST00000551107	noncoding	0.994
SUPT16H	protein_coding	ENST00000549527	noncoding	0.983
SUPT16H	protein_coding	ENST00000484836	noncoding	0.990
SUPT16H	protein_coding	ENST00000412951	noncoding	0.985

SUPT16H	protein_coding	CPXM1	protein_coding	0.988
SUPT16H	protein_coding	CLMP	protein_coding	0.989
SUPT16H	protein_coding	CD302	protein_coding	0.991
SUPT16H	protein_coding	C10orf82	protein_coding	0.982
SUPT16H	protein_coding	ALDH1L1	protein_coding	0.988
SUPT16H	protein_coding	ACAD9	protein_coding	0.990
CPXM1	protein_coding	CAMLG	protein_coding	0.983
CPXM1	protein_coding	CLMP	protein_coding	0.989
PLEKHA5	protein_coding	CAMLG	protein_coding	0.982
PLEKHA5	protein_coding	HNRNPH3	protein_coding	0.985
PLEKHA5	protein_coding	ZFP82	protein_coding	0.985
PLEKHA5	protein_coding	VPS51	protein_coding	0.982
PLEKHA5	protein_coding	TNMD	protein_coding	0.989
PLEKHA5	protein_coding	OAT	protein_coding	0.981
PLEKHA5	protein_coding	NR_038402	noncoding	0.987
PLEKHA5	protein_coding	IFT88	protein_coding	0.980
PLEKHA5	protein_coding	HAAO	protein_coding	0.988
PLEKHA5	protein_coding	EPHX1	protein_coding	0.987
PLEKHA5	protein_coding	ENST00000564371	noncoding	0.982
PLEKHA5	protein_coding	ENST00000436013	noncoding	0.988
SYNGR1	protein_coding	SUPT16H	protein_coding	0.992
SYNGR1	protein_coding	RHOB	protein_coding	0.993
SYNGR1	protein_coding	RABGAP1L	protein_coding	0.989
SYNGR1	protein_coding	PPP1R8	protein_coding	0.980
SYNGR1	protein_coding	OAT	protein_coding	0.987
SYNGR1	protein_coding	NR_045021	noncoding	0.992
SYNGR1	protein_coding	NR_038402	noncoding	0.981
SYNGR1	protein_coding	HADHA	protein_coding	0.985
SYNGR1	protein_coding	FBXO38	protein_coding	0.989
SYNGR1	protein_coding	ENST00000564371	noncoding	0.992
SYNGR1	protein_coding	ENST00000551107	noncoding	0.989
SYNGR1	protein_coding	ENST00000549527	noncoding	0.987
SYNGR1	protein_coding	ENST00000510244	noncoding	0.980
SYNGR1	protein_coding	ENST00000484836	noncoding	0.983
SYNGR1	protein_coding	CPXM1	protein_coding	0.983
SYNGR1	protein_coding	CLMP	protein_coding	0.986
SYNGR1	protein_coding	CD302	protein_coding	0.986
SYNGR1	protein_coding	ALDH1L1	protein_coding	0.989
SYNGR1	protein_coding	ACAD9	protein_coding	0.986
IFT88	protein_coding	ENST00000436013	noncoding	0.986
IFT88	protein_coding	C10orf82	protein_coding	0.981
ZC4H2	protein_coding	CAMLG	protein_coding	0.991
ZC4H2	protein_coding	PLEKHA5	protein_coding	0.981
ZC4H2	protein_coding	ZNF232	protein_coding	0.985

ZC4H2	protein_coding	ZFP82	protein_coding	0.986
ZC4H2	protein_coding	TAB2	protein_coding	0.982
ZC4H2	protein_coding	SUPT16H	protein_coding	0.988
ZC4H2	protein_coding	RHOB	protein_coding	0.989
ZC4H2	protein_coding	RABGAP1L	protein_coding	0.989
ZC4H2	protein_coding	OAT	protein_coding	0.989
ZC4H2	protein_coding	NR_045021	noncoding	0.981
ZC4H2	protein_coding	NR_038402	noncoding	0.990
ZC4H2	protein_coding	GSTM3	protein_coding	0.982
ZC4H2	protein_coding	FBXO38	protein_coding	0.994
ZC4H2	protein_coding	EPHX1	protein_coding	0.980
ZC4H2	protein_coding	ENST00000568835	noncoding	0.990
ZC4H2	protein_coding	ENST00000564371	noncoding	0.982
ZC4H2	protein_coding	ENST00000510244	noncoding	0.983
ZC4H2	protein_coding	CPXM1	protein_coding	0.986
ZC4H2	protein_coding	CD302	protein_coding	0.985
ALDH1L1	protein_coding	ACAD9	protein_coding	0.984
CLMP	protein_coding	CDIPT	protein_coding	0.993
CLMP	protein_coding	CD302	protein_coding	0.982
CLMP	protein_coding	ALDH1L1	protein_coding	0.988
CD302	protein_coding	C10orf82	protein_coding	0.992
CD302	protein_coding	BAG2	protein_coding	0.985
TNMD	protein_coding	OAT	protein_coding	0.982
TNMD	protein_coding	NR_038402	noncoding	0.985
TNMD	protein_coding	HAAO	protein_coding	0.982
TNMD	protein_coding	ESD	protein_coding	0.988
TNMD	protein_coding	ENST00000564371	noncoding	0.987
TNMD	protein_coding	ENST00000436013	noncoding	0.989
TNMD	protein_coding	COL6A2	protein_coding	0.982
TNMD	protein_coding	CD302	protein_coding	0.985
TNMD	protein_coding	ANGPTL5	protein_coding	0.988
NR_049782	noncoding	ENST00000549527	noncoding	0.986
NR_049782	noncoding	CLMP	protein_coding	0.981
NR_049782	noncoding	CDIPT	protein_coding	0.988
NR_049782	noncoding	BAG2	protein_coding	0.983
NR_049782	noncoding	ABI1	protein_coding	0.982
ENST00000436013	noncoding	CD302	protein_coding	0.981
ENST00000436013	noncoding	C10orf82	protein_coding	0.985
ENST00000436013	noncoding	ANGPTL5	protein_coding	0.997
ENST00000549527	noncoding	CLMP	protein_coding	0.984
ENST00000549527	noncoding	CDIPT	protein_coding	0.981
ENST00000549527	noncoding	CD302	protein_coding	0.985
ENST00000549527	noncoding	BAG2	protein_coding	0.989
ENST00000549527	noncoding	ACAD9	protein_coding	0.980

uc022atk.1	noncoding	SUPT16H	protein_coding	0.986
uc022atk.1	noncoding	STT3B	protein_coding	0.996
uc022atk.1	noncoding	STAR	protein_coding	0.988
uc022atk.1	noncoding	NR_046473	noncoding	0.994
uc022atk.1	noncoding	NR_045021	noncoding	0.984
uc022atk.1	noncoding	NR_027158	noncoding	0.992
uc022atk.1	noncoding	MAF	protein_coding	0.987
uc022atk.1	noncoding	HADHA	protein_coding	0.989
uc022atk.1	noncoding	HAAO	protein_coding	0.986
uc022atk.1	noncoding	EPHX1	protein_coding	0.986
uc022atk.1	noncoding	ENST00000564371	noncoding	0.989
uc022atk.1	noncoding	ENST00000551107	noncoding	0.991
uc022atk.1	noncoding	ENST00000484836	noncoding	0.991
uc022atk.1	noncoding	ENST00000436013	noncoding	0.994
uc022atk.1	noncoding	DCTN4	protein_coding	0.981
uc022atk.1	noncoding	CD302	protein_coding	0.981
uc022atk.1	noncoding	C10orf82	protein_coding	0.983
uc022atk.1	noncoding	ANGPTL5	protein_coding	0.988
ENST00000484836	noncoding	CPXM1	protein_coding	0.993
ENST00000484836	noncoding	CLMP	protein_coding	0.992
ENST00000484836	noncoding	CDIPT	protein_coding	0.987
ENST00000484836	noncoding	CD302	protein_coding	0.981
ENST00000484836	noncoding	ALDH1L1	protein_coding	0.980
uc010oay.1	noncoding	STAR	protein_coding	0.985
uc010oay.1	noncoding	NR_049782	noncoding	0.982
uc010oay.1	noncoding	NR_046464	noncoding	0.982
uc010oay.1	noncoding	NR_027158	noncoding	0.981
uc010oay.1	noncoding	MAF	protein_coding	0.984
uc010oay.1	noncoding	CDIPT	protein_coding	0.988
uc010oay.1	noncoding	ABI1	protein_coding	0.996
ENST00000568835	noncoding	CAMLG	protein_coding	0.986
ENST00000568835	noncoding	ENST00000510244	noncoding	0.991
NR_046473	noncoding	NR_046464	noncoding	0.987
NR_046473	noncoding	NR_027158	noncoding	0.991
NR_046473	noncoding	MAF	protein_coding	0.993
NR_046473	noncoding	HADHA	protein_coding	0.982
NR_046473	noncoding	HAAO	protein_coding	0.982
NR_046473	noncoding	ENST00000564371	noncoding	0.980
NR_046473	noncoding	ENST00000551107	noncoding	0.980
NR_046473	noncoding	ENST00000520714	noncoding	0.992
NR_046473	noncoding	ENST00000484836	noncoding	0.985
NR_046473	noncoding	ENST00000436013	noncoding	0.983
NR_046473	noncoding	DCTN4	protein_coding	0.992
NR_046473	noncoding	ANGPTL5	protein_coding	0.981

NR_045021	noncoding	NR_027158	noncoding	0.994
NR_045021	noncoding	MAF	protein_coding	0.986
NR_045021	noncoding	HADHA	protein_coding	0.991
NR_045021	noncoding	FBXO38	protein_coding	0.984
NR_045021	noncoding	ENST00000564371	noncoding	0.987
NR_045021	noncoding	ENST00000551107	noncoding	0.987
NR_045021	noncoding	ENST00000549527	noncoding	0.988
NR_045021	noncoding	ENST00000484836	noncoding	0.996
NR_045021	noncoding	CPXM1	protein_coding	0.994
NR_045021	noncoding	CLMP	protein_coding	0.995
NR_045021	noncoding	CDIPT	protein_coding	0.989
NR_045021	noncoding	CD302	protein_coding	0.987
NR_045021	noncoding	C10orf82	protein_coding	0.984
NR_045021	noncoding	ALDH1L1	protein_coding	0.982
ENST00000520714	noncoding	DCTN4	protein_coding	0.996
ENST00000520714	noncoding	CLMP	protein_coding	0.981
ENST00000520714	noncoding	CDIPT	protein_coding	0.983
NR_027158	noncoding	MAF	protein_coding	0.996
NR_027158	noncoding	HADHA	protein_coding	0.989
NR_027158	noncoding	HAAO	protein_coding	0.982
NR_027158	noncoding	EPHX1	protein_coding	0.982
NR_027158	noncoding	ENST00000564371	noncoding	0.982
NR_027158	noncoding	ENST00000551107	noncoding	0.983
NR_027158	noncoding	ENST00000520714	noncoding	0.982
NR_027158	noncoding	ENST00000484836	noncoding	0.999
NR_027158	noncoding	CPXM1	protein_coding	0.988
NR_027158	noncoding	CLMP	protein_coding	0.990
NR_027158	noncoding	CDIPT	protein_coding	0.989
NR_027158	noncoding	CD302	protein_coding	0.983
NR_027158	noncoding	C10orf82	protein_coding	0.984
NR_027158	noncoding	ABI1	protein_coding	0.984
NR_046464	noncoding	MAF	protein_coding	0.983
NR_046464	noncoding	ESD	protein_coding	0.981
NR_046464	noncoding	ENST00000564371	noncoding	0.981
NR_046464	noncoding	ENST00000520714	noncoding	0.995
NR_046464	noncoding	DCTN4	protein_coding	0.993
NR_046464	noncoding	COL6A2	protein_coding	0.990
NR_046464	noncoding	CLMP	protein_coding	0.983
NR_046464	noncoding	CDIPT	protein_coding	0.985
NR_046464	noncoding	CD302	protein_coding	0.988
NR_046464	noncoding	BAG2	protein_coding	0.986
ENST00000564371	noncoding	ENST00000551107	noncoding	0.994
ENST00000564371	noncoding	ENST00000484836	noncoding	0.984
ENST00000564371	noncoding	ENST00000436013	noncoding	0.988

ENST00000564371	noncoding	ENST00000412951	noncoding	0.981
ENST00000564371	noncoding	CLMP	protein_coding	0.984
ENST00000564371	noncoding	CD302	protein_coding	0.992
ENST00000564371	noncoding	C10orf82	protein_coding	0.980
ENST00000564371	noncoding	ANGPTL5	protein_coding	0.980
ENST00000564371	noncoding	ALDH1L1	protein_coding	0.992
ENST00000564371	noncoding	ACAD9	protein_coding	0.985
NR_038402	noncoding	CAMLG	protein_coding	0.987
NR_038402	noncoding	KLHL20	protein_coding	0.987
NR_038402	noncoding	GSTM3	protein_coding	0.990
NR_038402	noncoding	FBXO38	protein_coding	0.990
NR_038402	noncoding	ENST00000568835	noncoding	0.989
NR_038402	noncoding	ENST00000564371	noncoding	0.987
NR_038402	noncoding	ENST00000510244	noncoding	0.988
NR_038402	noncoding	CD302	protein_coding	0.984
ENST00000578315	noncoding	ENST00000568835	noncoding	0.991
ENST00000578315	noncoding	ENST00000510244	noncoding	0.985
ENST00000412951	noncoding	DCTN4	protein_coding	0.982
ENST00000412951	noncoding	CLMP	protein_coding	0.981
ENST00000412951	noncoding	AGPAT4	protein_coding	0.983
ENST00000551107	noncoding	ENST00000484836	noncoding	0.986
ENST00000551107	noncoding	ENST00000436013	noncoding	0.985
ENST00000551107	noncoding	ENST00000412951	noncoding	0.989
ENST00000551107	noncoding	CLMP	protein_coding	0.980
ENST00000551107	noncoding	CD302	protein_coding	0.984
ENST00000551107	noncoding	ALDH1L1	protein_coding	0.986
ENST00000551107	noncoding	ACAD9	protein_coding	0.982

Interaction: the values of interation coeffient indicates the degree of two gene co-expression. The larger of absolute value, the stronger of co-expression.Positive represent a positive regulationand negetive indicate a negetive regulation.

Table S6B. The property of mRNA and lncRNA of profile NO.9

Genesymbol/lncRNA	clusteringCoefficient	degree	type
ENST00000564371	0.395	36	noncoding
CD302	0.431	34	protein_coding
RHOB	0.456	33	protein_coding
OAT	0.468	32	protein_coding
SUPT16H	0.483	30	protein_coding
FBXO38	0.467	27	protein_coding
TAB2	0.529	26	protein_coding
NR_045021	0.577	25	noncoding
NR_027158	0.500	25	noncoding
CLMP	0.496	24	protein_coding
HNRNPH3	0.464	24	protein_coding
HADHA	0.628	22	protein_coding
STT3B	0.615	22	protein_coding
SYNGR1	0.597	22	protein_coding
ENST00000484836	0.614	21	noncoding
ZNF232	0.626	20	protein_coding
uc022atk.1	0.611	20	noncoding
C10orf82	0.605	20	protein_coding
FERMT2	0.447	20	protein_coding
ZC4H2	0.556	19	protein_coding
NR_038402	0.526	19	noncoding
OS9	0.520	19	protein_coding
RABGAP1L	0.634	18	protein_coding
HAAO	0.614	18	protein_coding
ZFP82	0.484	18	protein_coding
NR_046464	0.399	18	noncoding
ENST00000551107	0.632	17	noncoding
ENST00000436013	0.574	17	noncoding
EPHX1	0.559	17	protein_coding
NR_046473	0.544	17	noncoding
ENST00000549527	0.683	16	noncoding
PMP22	0.686	15	protein_coding
CPXM1	0.676	15	protein_coding
ALDH1L1	0.629	15	protein_coding
MAF	0.486	15	protein_coding
BAG2	0.670	14	protein_coding
RAB3GAP1	0.756	13	protein_coding
CNBP	0.705	13	protein_coding
TNMD	0.551	13	protein_coding
PLEKHA5	0.526	13	protein_coding
CDIPT	0.513	13	protein_coding
STAR	0.652	12	protein_coding

ANGPTL5	0.818	11	protein_coding
ENST00000568835	0.636	11	noncoding
CAMLG	0.527	11	protein_coding
COL6A2	0.509	11	protein_coding
OCRL	0.455	11	protein_coding
ACAD9	0.822	10	protein_coding
GSTM3	0.644	10	protein_coding
ENST00000520714	0.639	9	noncoding
ENST00000510244	0.639	9	noncoding
DCTN4	0.571	8	protein_coding
NR_049782	0.571	8	noncoding
KLHL20	0.762	7	protein_coding
uc010oay.1	0.619	7	noncoding
ENST00000412951	0.286	7	noncoding
ABI1	0.733	6	protein_coding
PPP1R8	0.733	6	protein_coding
ENST00000578315	0.667	6	noncoding
LRRTM3	0.800	5	protein_coding
ESD	0.833	4	protein_coding
VPS51	0.833	4	protein_coding
IFT88	0.833	4	protein_coding
ASH2L	1.000	2	protein_coding
PRPF31	0.000	2	protein_coding
NR_015447	0.000	1	noncoding
AGPAT4	0.000	1	protein_coding

Clustering Coefficient: The greater of clustering coefficient, the closer relationship between genes of co-expression; **Degree:** It indicates the number of the gene interact with this gene in this co-expression networks.
